# Metal Propionate Solutions for High-Throughput Liquid-Assisted Manufacturing of Superconducting REBa_2_Cu_3_O_7-δ_ (RE = Y, Gd, Sm, and Yb) Films

**DOI:** 10.1021/acsami.4c11685

**Published:** 2024-09-26

**Authors:** Lavinia Saltarelli, Daniel Sanchez-Rodriguez, Kapil Gupta, Aiswarya Kethamkuzhi, Jordi Farjas, Elies Molins, Ramón Yañez, Susagna Ricart, Xavier Obradors, Teresa Puig

**Affiliations:** † Institut de Ciència de Materials de Barcelona, ICMAB-CSIC, Campus de la UAB, Bellaterra, Catalonia 08193, Spain; ‡ GRMT, Department of Physics, University of Girona, Girona, Catalonia E17071, Spain; § Departament de Química, Facultat de Ciències, Universitat Autònoma de Barcelona, Cerdanyola del Vallès, Catalonia 08193, Spain

**Keywords:** metal propionates, thermogravimetric analysis, microstructure, chemical solution deposition, superconducting materials, transient liquid assisted growth

## Abstract

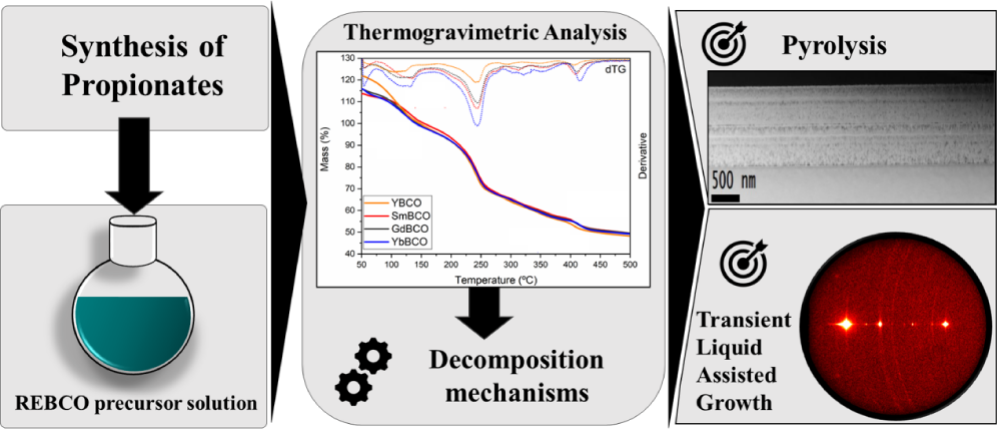

The cost-effective synthesis of a series of metal propionate powders (copper, yttrium, barium, samarium, gadolinium, and ytterbium) is developed through single chemical reactions resulting in five novel crystalline forms. These complexes are valuable precursors for the preparation of epitaxial REBa_2_Cu_3_O_7-δ_ (REBCO) superconducting films (here, RE = Y, Sm, Gd, and Yb) through the innovative transient liquid-assisted growth (TLAG) process based on chemical solution deposition (CSD). TLAG-CSD shows impressive results with YBa_2_Cu_3_O_7-δ_ (YBCO), obtaining critical current densities of 2.6 MA/cm^2^ (77 K) on 500 nm films at unprecedented growth rates (50–2000 nm/s), boosting unprecedented high-throughput industrial production. With a cardinal concern on designing the pyrolysis toward optimal nanocrystalline films for TLAG, an analysis of the thermal behavior of the synthesized precursors is essential. Decomposition pathways for each metal propionate are established, and compatibility with TLAG-CSD is corroborated. Metal–organic solutions for these REBCO systems are successfully prepared, and their rheological properties and thermal behavior are analyzed. This work demonstrates homogeneous nanocrystalline films through propionate-based REBCO precursor solutions, including several rare-earth ions, which display exemplary chemical and microstructural characteristics crucial for TLAG, and provides a base for a wide variety of CSD-based functional oxides.

## Introduction

The transition toward renewable energy sources is among the most discussed topics of the last decades, being one of the most urgent realities faced by the scientific community. In this framework, functional materials play an important role for their possible applications in clean energy production, harvesting, storage, and transport, and extensive reports about chemical approaches to the synthesis of these materials have been presented.^1,2^ Regarding the latter, superconductors are in the limelight, as their distinguished characteristic of lossless electrical transport could lead to a substantial decrease of energy losses,^3−5^ which are instead present by default in currently employed conductive materials, such as copper cables. More specifically, cuprate high-temperature superconductors (HTS) based on REBa_2_Cu_3_O_7-*x*_ (REBCO, RE = Y or other rare-earth elements) systems show the most encouraging features since their discovery in 1986,^6^ as their critical temperature above liquid nitrogen temperatures combined with their extremely achievable high magnetic fields (irreversibility line) makes the attainment of unprecedented limits for low-temperature superconductors (LTS) possible, driving novel energy devices at operating temperatures from 20 to 77 K.^7^

The implementation of HTS REBCO systems on flexible substrate architectures for their industrialization, namely, coated conductors (CCs), has been challenging, with the main difficulty consisting of encountering a feasible way to adapt the industrial need of high-throughput processes to the preparation of the biaxially textured REBCO superconducting layer in a reel-to-reel configuration.^8−12^ Chemical solution deposition (CSD) emerged as a promising method to drastically reduce the production costs of REBCO superconducting films.^13,14^ High-performance REBCO films could be obtained using inexpensive metal–organic chemical solutions, in which the metal cations are responsible for the formation of the final superconducting film through different annealing processes. Early efforts were devoted to the development of CSD through the route known as trifluoroacetate metal–organic decomposition (TFA-MOD),^15,16^ which employs commercial fluorinated metal acetate precursors for the preparation of the REBCO precursor solutions.^16−19^ High-performance properties could be attained, with values of the critical current density (*J*_c_) up to 10 MA/cm^2^ at 77 K in self-field,^20^ and the application of TFA-MOD in industrial context is still nowadays prevalent.^21^ An adverse downside of the TFA-MOD route is found in the REBCO growth mechanism, occurring through a solid–solid reaction, thus inherently limiting the growth rates below 1–5 nm/s.^12,22,23^ Moreover, since HF out-diffusion is the limiting step, the rate of the process is importantly decreased in thicker films. For this purpose, the use of commercial metal acetates in propionic acid or propionic acid-based solvent mixtures and, in some cases, employing polymers or amines as additives are among the most common approaches nowadays.^24−29^ The main drawback of the fluorine-free route is the formation of BaCO_3_ as the barium precursor for the following growth of the superconducting REBCO films, undesirable due to its high thermal stability;^16,30^ regardless of this, it was demonstrated that high-performance YBCO films could be obtained through the use of this type of precursor solutions, by employing a novel growth process known as transient liquid-assisted growth (TLAG).^31^ Nonetheless, the use of commercial metal acetates in the solution preparation procedure resulted in high-performance physical properties, exhibited by films of 100 nm thickness of final YBCO. The critical current density of 5 MA/cm^2^ obtained on this thickness proved that TLAG is a true possibility for industrial applications if the thickness of the layers can be enhanced while retaining these superconducting properties, which was not viable mainly due to solubility limitations of the metal acetate precursors in the chosen media. Recently, through the optimization and development of a novel metal propionate-based precursor solution adaptable to the high-throughput TLAG process, thick pyrolyzed films of 400 nm per layer and up to 2.7 μm through multideposition with nanoscale homogeneity, high-performance, and reproducible properties could be demonstrated for the YBCO system,^32^ overcoming the thickness limitation and showing the feasibility of TLAG-CSD in the industrial framework through the compatibility with scalable deposition techniques (inkjet printing).^33,34^ Furthermore, the breakthrough for YBCO fabrication through TLAG was the confirmation of unprecedented growth rates beyond 2000 nm/s,^35^ undeniably boosting the possibilities for a high-throughput commercial implementation of TLAG.^12^ The key aspect in this novel fluorine-free solution was the optimization of the presynthesized metal propionate precursors of Y, Ba, and Cu for their application in the YBCO precursor solution, as when dissolving metal acetates in propionic acid, a full conversion of the acetates into propionates is not always granted: specifically, for the case of barium acetate, a mixed complex of acetate-propionate is yielded in the aforementioned conditions.^36,37^ The presence of undesired products in the YBCO precursor solution, such as nonreacted acetates, acetate-propionate mixtures, and acetic acid (intrinsically present due to the conversion reaction of metal acetate into metal propionate), leads to a more complex and less reproducible decomposition of the metal–organic derivatives in the first, low-temperature pyrolysis thermal treatment, hampering the preparation of the nanocrystalline precursor films with complications such as crack formation or presence of undesired phases and so leading to poor superconducting properties.^38^ As an alternative, the previous synthesis of pure metal propionates powders and their successive application as precursors in the chemical solution ensured reproducible decomposition mechanisms given the absence of any kind of secondary products in the solution, resulting in homogeneous and reproducible nanocrystalline precursor films.^32^

Extensive information is present in the literature regarding the YBCO superconducting system, but in the last decades, the interest in developing REBCO superconducting films has incremented importantly.^11,21,39−41^ An explanation may be found in the wide spectrum of possible applications that REBCO superconductors facilitate if compared to YBCO: when Sm or Gd are employed, considering the intrinsically higher critical temperature (*T*_c_) in these systems, given their higher irreversibility line, a more extensive range of magnetic field values can be employed, consequently improving critical current density and flux pinning phenomena.^20,42−45^ The replacement of Y for Yb, instead, may lead to a significant decrease in the processing temperatures, being the YbBCO nucleation conditions optimal at lower temperature when compared to YBCO, particularly interesting when aiming for more efficient industrial processes.^39,46^ Moreover, strategies toward the widening of the processing window include the use of solutions with RE mixtures, such as the widely spread Y_0.7_Gd_0.3_Ba_2_Cu_3_O_7-δ_ ((Y,Gd)BCO).

**Scheme 1:**
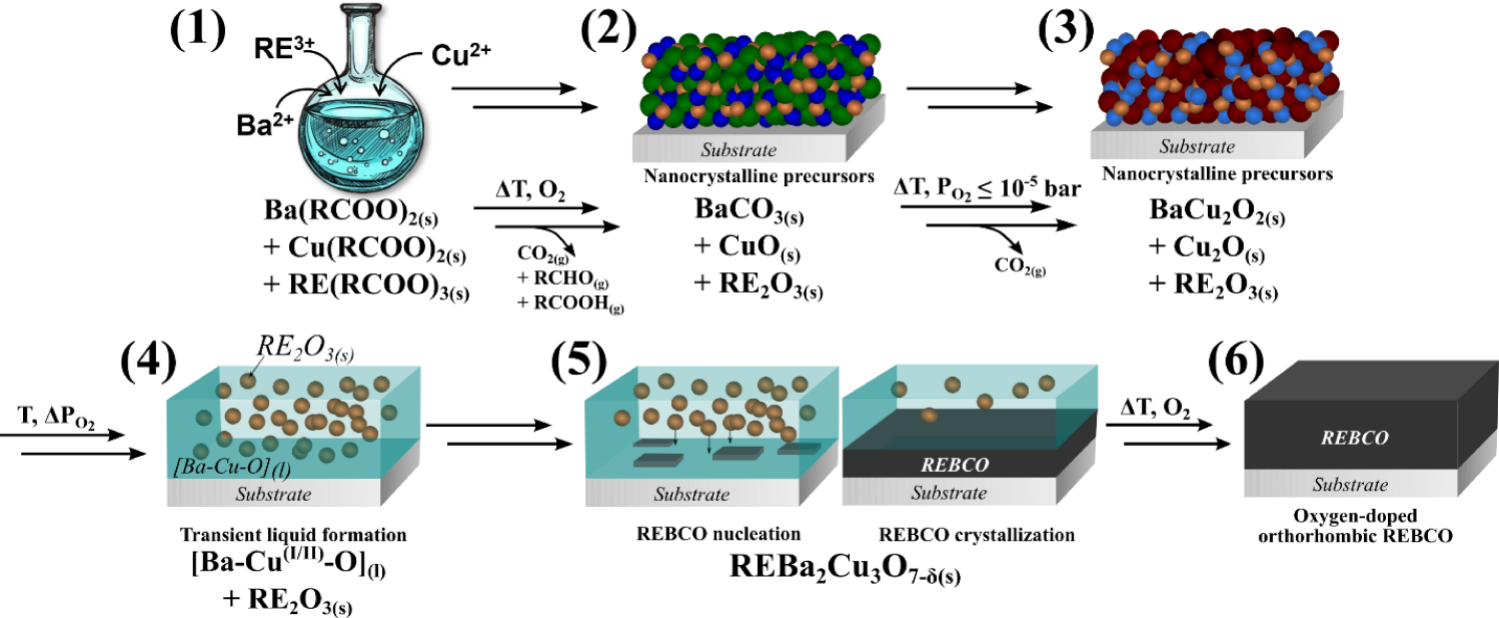
Overview of the Six Different, Consecutive Steps Followed to Grow Epitaxial REBa_2_Cu_3_O_7_ Films through the TLAG Process Using the  Route, Indicating the Phase Transformation Occurring in Each Step: (1) Metal–Organic Solution Preparation; (2) Deposition and Pyrolysis of the Metal–Organic Precursors; (3) Temperature Annealing at Low  Following a Well-Controlled Heating Ramp; (4) Isothermal Annealing Following a  Step to Form the Transient [Ba-Cu-O] Liquid Where Solid RE_2_O_3_ Nanoparticles Remain Trapped; (5) Nucleation and Growth of the Epitaxial REBCO Film in a Selected *T* –  Condition; (6) Oxygenation of Grown REBCO Film to Induce O_2_ Doping and Tetragonal to Orthorhombic Phase Transformation, the Latter Displaying Superconducting Properties

Accordingly, pursuing the fabrication of REBCO superconducting films through TLAG-CSD requires full control of the intermediate precursor quality as well as of the different processing steps. Scheme 1 describes the six consecutive steps to be followed to grow epitaxial REBCO films by TLAG-CSD. All of them need to be carefully defined to reach high superconducting performances.

In this article, the synthetic routes for metal propionates of Y, Ba, Cu, Gd, Sm, and Yb are described in detail. Through the use of one-pot syntheses and standard purification procedures, high purity powder products of all of the metal propionates mentioned above could be obtained. The resulting compounds were characterized through various techniques to confirm their purity. Moreover, their compatibility with the following steps to prepare REBCO films by the TLAG-CSD process has been demonstrated.

Additionally, *per se* the synthetic methods and final products described here could be applied to any functional material that follows the metal–organic precursor decomposition pathway based on CSD methodology, for instance, buffer layers for REBCO CCs,^47−49^ or other applications in fuel cells, catalysis, and ionic conductors.^50^ Overall, the reported synthetic methods can pave the way for new possibilities for low-cost and high-throughput functional metal oxides-based technologies.

## Experimental Section

### Materials and General Details

Propionic acid, ≥ 99.5% (HProp), ytterbium(III) acetate hydrate 99.95% trace metal basis (Yb(OAc)_3_), and barium acetate (Ba(OAc)_2_) ACS reagent ≥99.5% were purchased from Sigma-Aldrich. Barium(II) carbonate, 99.95% trace metal basis (BaCO_3_), copper(II) oxide, Puratronic, 99.7% metal basis (CuO), yttrium(III) oxide, REacton, 99.99% (REO) (Y_2_O_3_), gadolinium(III) oxide, REacton, 99.99% (REO) (Gd_3_O_2_), and samarium(III) acetate, REacton, 99.9% (REO) (Sm(OAc)_3_) were purchased from Alfa Aesar. Acetic acid glacial (99.5%) was purchased from Panreac Sintesis. Diethyl ether, AGR, ACS, ISO, stabilized with BHT (Et_2_O) was purchased from Labbox. Acetone, Multisolvent HPLC grade ACS ISO UV–vis, was purchased from Scharlab. Methanol 99.9%, anhydrous (max 0.003% H_2_O) (MeOH), and monoethanolamine purified by redistillation, ≥99.5% (MEA), used in the preparation of the REBCO precursor solutions were purchased, respectively, from Scharlab and Sigma-Aldrich. All of the above reagents and solvents were used without further purification.

The final powder products of all metal propionates are characterized through various techniques. FTIR-ATR spectra were measured on a Jasco 4700 spectrophotometer equipped with an attenuated total reflectance (ATR) accessory (energy range 4000–500 cm^–1^). Powder X-ray diffraction patterns (PXRD) were measured on a Siemens D-5000 diffractometer (Cu Kα radiation of λ = 1.5418 Å, 2θ = 5° to 60°, step scan 0.02°, acquisition time 1 s per step). Elemental analysis (EA) was measured on a PerkinElmer 2400 series instrument. Scanning electron microscopy (SEM) (QUANTA FEI 200 FEG-ESEM) was employed for the evaluation of the grain size. Thermogravimetric analysis (TGA) was performed on a Mettler-Toledo thermobalance, model TGA/DSC1 (temperature range 50 to 600 °C, constant heating rate of 10 °C/min for powders and 5 °C/min for films). In situ EGA/FTIR was obtained by connecting the TGA gas outlet to an ALPHA Bruker FTIR gas analyzer (40 cm long steel tube heated to 200 °C). Powders were placed inside open alumina crucibles, and a flowing atmosphere (60 mL/min) of high purity N_2_ (Air Liquide, ≥99.999%) was established. Films were obtained by drop casting each solution onto 10 × 10 mm LaAlO_3_ (LAO) substrates and placed on the TGA sensor. Prior to the TG process, deposited films are placed on a preheated heating plate (70 °C, 5 min) to eliminate solvent excess. For films, a flow (60 mL/min) of high purity dry or humid O_2_ (Air Liquide, ≥99.999%) was established. Water-saturated flows were obtained by bubbling the carrier gas in deionized water at 25 °C and atmospheric pressure. EGA-FTIR signals are identified according to.^51^ Evolution of the species of interest evolved during the TGA experiments was obtained by tracing the following selected absorption peak frequencies: 1732 cm^–1^ for 3-pentanone, 1780 cm^–1^ for propionic acid, 2180 cm^–1^ for CO, 2355 cm^–1^ for CO_2_, 2740 cm^–1^ for acetaldehyde, 3903 cm^–1^ for H_2_O, 1508 cm^–1^ for formaldehyde, and 1796 cm^–1^ for acetic acid.

Single crystals of all of the propionate compounds described in this manuscript were selected and mounted for X-ray single-crystal diffraction experiments. Crystallographic data were collected on a Bruker APEX-II CCD diffractometer using graphite monochromated Mo Kα radiation (λ = 0.71073 Å). Crystallographic data were collected at 294(2) K. Data reduction was performed using SAINT V6.45A and SORTAV^52^ in the diffractometer package. Data were corrected for Lorentz and polarization effects and for absorption by SADABS.^53^ The structural resolution procedure was made using SHELXT.^54^ Non-hydrogen atoms were refined anisotropically. Hydrogen atoms were introduced in calculated positions and refined riding on their parent atoms, except for those belonging to water molecules, which were refined under restrictions of forming the hydrogen bonds expected from a crystallographic perspective.^55^ Selected crystal and data collection parameters are reported in corresponding Tables S1 and S10 and Section I.

Complete crystallographic data for the structural analysis have been deposited with the Cambridge Crystallographic Data Centre, CCDC n° 2304806-2304811. Copies of this information may be obtained free of charge from the Director, Cambridge Crystallographic Data Centre, 12 Union Road, Cambridge, CB21EZ, UK (fax: +44-1223-336033, e-mail: deposit@ccdc.cam.ac.uk or via: www.ccdc.cam.ac.uk).

Films deriving from solutions of individual metal propionate and nanocrystalline precursor layers of REBCO (RE = Y, Gd, Sm, and Yb) were obtained by depositing each precursor solution via spin coating in a grade ISO7 clean room at 10% humidity at a spinning rate of 6000 rpm for 2 min on single-crystal (001) SrTiO_3_ (STO) substrates (CrysTech GmbH). Before deposition, substrates undergo an annealing process at 900 °C for 5 h to obtain flat-terraced surfaces, which are successively cleaned with acetone and methanol to eliminate any possible residues.

The annealing (pyrolysis) that yields the nanocrystalline precursor films was performed by heating the samples in a tubular furnace in a humid oxygen flow (0.12 L/min) up to 240 °C at a rate of 3 °C/min and up to 500 °C at a rate of 5 °C/min, followed by cooling to room temperature. To obtain REBCO nanocrystalline precursor films with a thickness of approximately 700 nm, a multideposition process (deposition and pyrolysis) was carried out by repeating the same procedure twice.

Characterization of nanocrystalline precursor films was performed by optical microscopy (OM) (Leica DM1750 M) analysis to inspect homogeneity of the films.

X-ray diffraction (XRD) was performed on a Bruker D8 Discover system (Cu Kα, X-ray energy = 8.049 keV) equipped with a Lynxeye XE-T energy-dispersive one-dimensional (1D) detector, measuring in grazing incidence (GI) geometry to characterize the structure and phase composition of the as-prepared films of precursor REBCO layers or solutions of each metal propionate.

The microstructure of the REBCO precursor films was investigated using various techniques of transmission electron microscopy (TEM). The spatial distribution, homogeneity, and respective sizes of the nanocrystalline phases were analyzed by using high-resolution transmission electron microscopy (HRTEM), high-angle annular dark field scanning transmission electron microscopy (HAADF-STEM), energy-dispersive X-ray spectroscopy (EDX), and electron energy loss spectroscopy (EELS). FEI Tecnai F20 (S)TEM operated at 200 kV was used, equipped with a Gatan imaging filter (GIF) Quantum SE along with a 2kx2k CCD camera for EELS analyses and an EDAX super ultra-thin window X-ray detector for EDX analyses. Atomic-resolution HAADF-STEM images were acquired using a double-aberration-corrected Thermo Fisher Scientific Spectra 300 STEM operated at 200 kV.

The pore density was evaluated from the cross-sectional HAADF-STEM micrographs, and image analysis software ImageJ^56^ was used, where pores were defined by dark contrast areas.

HRTEM, STEM, and EELS data analysis was conducted using Gatan digital micrograph (DM) software. A principal component analysis (PCA) based on multivariate statistical analysis (MSA) was also used for EELS data processing in order to reduce the statistical noise in the EELS spectrum images. A reconstruction using the relevant principal components was performed using the weighted-PCA MSA plugin^57^ in DM, and EELS elemental maps were extracted from this reconstructed data.

Growth of the thin films was performed following the TLAG process previously described.^31,35^ We used the  route, and the experiments were carried out in a tubular furnace equipped with a vacuum system that enables rapid switching (time range of seconds) from a low vacuum to a high vacuum. The samples are heated at low  (10^–5^ bar), with an average heating rate of 1 °C/s to the desired temperature (765–870 °C) (step 3 in Scheme 1). The jump in  follows, reaching the desired final  (1–2 mbar) in a time range of 1 s (steps 4 and 5 in Scheme 1). Following the TLAG process, an oxygenation process is performed for tetragonal to orthorhombic phase transition of REBCO. The samples are heated in a tubular furnace at a total pressure of 1 bar with a heating rate of 10 °C/min to 450 °C, followed by a dwell of 210 min, and then cooling with the same heating rate to room temperature. This process is performed under a continuous O_2_ flow of 0.6 L/min (step 6 in Scheme 1).

Electron transparent cross-sectional TEM specimens were prepared by a focused ion beam (FIB, Helios 5 UX) as well as conventional methods, including cutting with a precision vertical diamond wire saw (Well, model 3242), gluing, mechanical polishing with an Allied Multiprep precision polishing system, and finally Ar^+^ ion milling using a Gatan precision ion polishing system (PIPS).

The critical temperature (*T*_c_) is calculated from electrical resistivity measurements in a van der Pauw configuration using a Quantum Design Physical Property Measurement System (PPMS) and confirmed by SQUID magnetic induced measurements. The transport critical current density (*J*_c_) as a function of temperature was determined from a SQUID magnetometer from Quantum Design equipped with a 7 T superconducting coil. The Bean critical state model was applied to deduce the critical current density field and the transport current.

### Synthesis of Metal Propionates (M(Prop)_*x*_)

#### Synthesis of M(Prop)_*x*_ (with M = Cu, Y, Sm, Gd, and Yb)

Metal propionate precursors (CuO, Y_2_O_3_, Sm(OAc)_3_, Gd_2_O_3_, or Yb(OAc)_3_) were added individually to HProp to reach a total concentration of [M^*x*+^] = 0.5 M (where *x* = 2 when M = Cu and *x* = 3 in the cases in which M = Y, Gd, Yb, or Sm). The reaction takes place overnight at 140 °C with vigorous stirring. Excess of solvent is eliminated through the use of a rotary evaporator to obtain a solid, except for the case of Y(Prop)_3_, in which a solid is obtained upon cooling. Purification to eliminate residual solvent is carried out by washing the solid three times with acetone and three times with Et_2_O with a Büchner funnel filtration system, except in the case of Cu in which only purification with Et_2_O is necessary. Yields between 68% and 96% were obtained for all compounds. Grain sizes were below 100 μm in all cases; nonetheless, in the case of Cu(Prop)_2_, mechanical grinding was employed to decrease the grain size below 30 μm and enhance its solubility. Suitable single crystals were obtained through several crystallization methods for the evaluation of the crystal structure. Details for each case are reported in Section I.

#### Synthesis of [Ba_7_(Prop)_14_(OH_2_)_8_]_*n*_(Ba(Prop)_2_)

BaCO_3_ is added to deionized water followed by the dropwise addition of HProp under vigorous stirring. HProp and distilled water are in a ratio of 1.125:1, and total [Ba^2+^] = 0.5 M. The reaction starts as a highly foamy, white solution upon the addition of HProp and is stirred vigorously for 24 h at RT to yield a transparent, clear solution. Solvent excess was eliminated using a rotary evaporator, to obtain a transparent gel. No solid could be obtained from the evaporation of the solvent; therefore, to induce crystallization, the product was placed in an ice bath for several hours. A white solid was then obtained, and its purification, as in the previous case using the Büchner funnel filtration system, needed a first washing of the product three times with acetone, followed by three times with Et_2_O. Yields of up to 88% were obtained following this procedure. In the case of employing only Et_2_O during purification, yields of only 20% were obtained. For more details, see Section I.

### Solutions of Individual Metal Propionates

To evaluate the decomposition of M(Prop)_*x*_ in films, solutions of the individual metal propionates were prepared by simply dissolving the metal propionate in a mixture of HProp and MeOH (1:1) under stirring at 30 °C, until complete dissolution was obtained (30 min approximately). To maximize the EGA-FTIR signal in the TGA experiments, the solutions were saturated; however, due to different solubilities of the precursors, the final concentrations were 1.5 M for the solution of Ba(Prop)_2_, 1 M for the solution of Gd(Prop)_3_, 0.5 M for the solution of Cu(Prop)_2_, and 0.25 M for the solutions of Y(Prop)_3_, Yb(Prop)_3_, and Sm(Prop)_3_.

### REBCO (RE = Y, Gd, Sm, or Yb) Precursor Solutions

REBCO precursor solutions for the preparation of nanocrystalline precursors layers of YBCO, GdBCO, SmBCO, and YbBCO were obtained by following the procedure developed in a previous detailed study dedicated to YBCO precursor solutions.^32^ Briefly, by dissolving Ba(Prop)_2_, Cu(Prop)_2_, and the respective RE propionate in a mixture of propionic acid and MeOH (1:1), with the addition of MEA in a molar ratio (Cu:MEA) = 1:0.61, homogeneous and stable solutions are obtained for the cases of RE = Y, Sm, Gd, or Yb. These are used as YBCO, SmBCO, GdBCO, and YbBCO precursor solutions.

All solutions described in this paper have a stoichiometry following the Cu-rich mixture, with a proportion of (Y or RE)-Ba-Cu of 1:2:4.66 ((3:7) composition). The concentration of the REBCO precursor solutions is 1.5 M in sum of metal salts for all solutions, and 3.4%_v/v_ MEA is used as an additive in each solution. We previously reported a thorough study on the influence of MEA in YBCO precursor solutions, and it was demonstrated that MEA aids solubility of metal salts in the media, solution stability, and homogeneity of the final films^32^ (step 1 in Scheme 1).

## Results and Discussion

### Characterization of the Synthesized Metal Propionates

Powder products of the propionates of copper (Cu(Prop)_2_), yttrium (Y(Prop)_3_), barium (Ba(Prop)_2_), samarium (Sm(Prop)_3_), gadolinium (Gd(Prop)_3_), and ytterbium (Yb(Prop)_3_) are obtained through one-pot syntheses starting from inexpensive precursors with high yields and high purity, and the elemental analysis results are in good agreement with the theoretical values for each product (see Experimental Section). The exact composition of the metal propionate compounds is determined jointly, considering the results of elemental analysis and the residual solvent observed during the initial mass losses in the TGA of the powders. In the cases of Ba(Prop)_2_, Sm(Prop)_3_, and Gd(Prop)_3_, the residual solvent or H_2_O observed by TGA is not present in the single-crystal structures, suggesting these are presumably lost during crystallization. FTIR-ATR spectra exhibit the characteristic bands for propionate groups corresponding to the aliphatic chain vibrational modes in the region between 2900 and 2800 cm^–1^ (individual identification for each metal propionate can be found in Experimental Section and Section I, and Figures S1 and S2). Moreover, for the cases of Sm(Prop)_3_ and Yb(Prop)_3_, the FTIR-ATR spectra in Figure S3 show no evidence of the presence of any acetate groups coming from the acetate precursors used for their synthesis, suggesting, within the detection limit, that there has been full replacement of the precursor functional groups by propionate groups. Analysis of the powders through SEM enabled us to determine average grain sizes, varying for each powder product and shown in Figure S4. Obtaining thick REBCO final films requires high concentrations, which are directly related to the solubility of the propionate precursors. In the case of Cu(Prop)_2_, the as-synthesized powder has an average grain size of 100 μm and a crystal-like shape (Figure S4a), and this has been observed to hinder a fast dissolution. The solubility of this particular precursor could be enhanced by drastically reducing the grain size by means of mechanical grinding, as shown in Figure S4b.

Suitable single crystals of several compounds were achieved, and the crystallographic structure was determined by X-ray diffraction to assess its influence on the decomposition process during pyrolysis. As it was mentioned previously, the growth of highly epitaxial REBCO thin films through TLAG requires nanoscale compositional homogeneity of the solid precursors; comprehensive knowledge on the crystal structure of the metal propionates is essential to disclose their decomposition mechanisms and ensure rigorous control of the microstructure of the nanocrystalline precursor films for TLAG. The single crystals could be obtained for all of the metal propionates through different crystallization methods. The structure obtained for Cu(Prop)_2_ matches the one published by Chung et al.^58^ (Figure S5a), and the one corresponding to Yb(Prop)_3_ matches the structure published by Bierke^59^ (Figure S5b); however, no matches in the literature could be found for the other crystal structures.

Sm(Prop)_3_ and Gd(Prop)_3_ both crystallize in a triclinic *P*-1 space group, whereas Y(Prop)_3_ and Ba(Prop)_2_ both crystallize in the monoclinic *P*21/n space group. Crystal data and structure refinement for the four compounds can be found in Table S1. Bond lengths and torsion angles for the four compounds are reported in Section I (Tables S2–S5).

**Figure 1:**
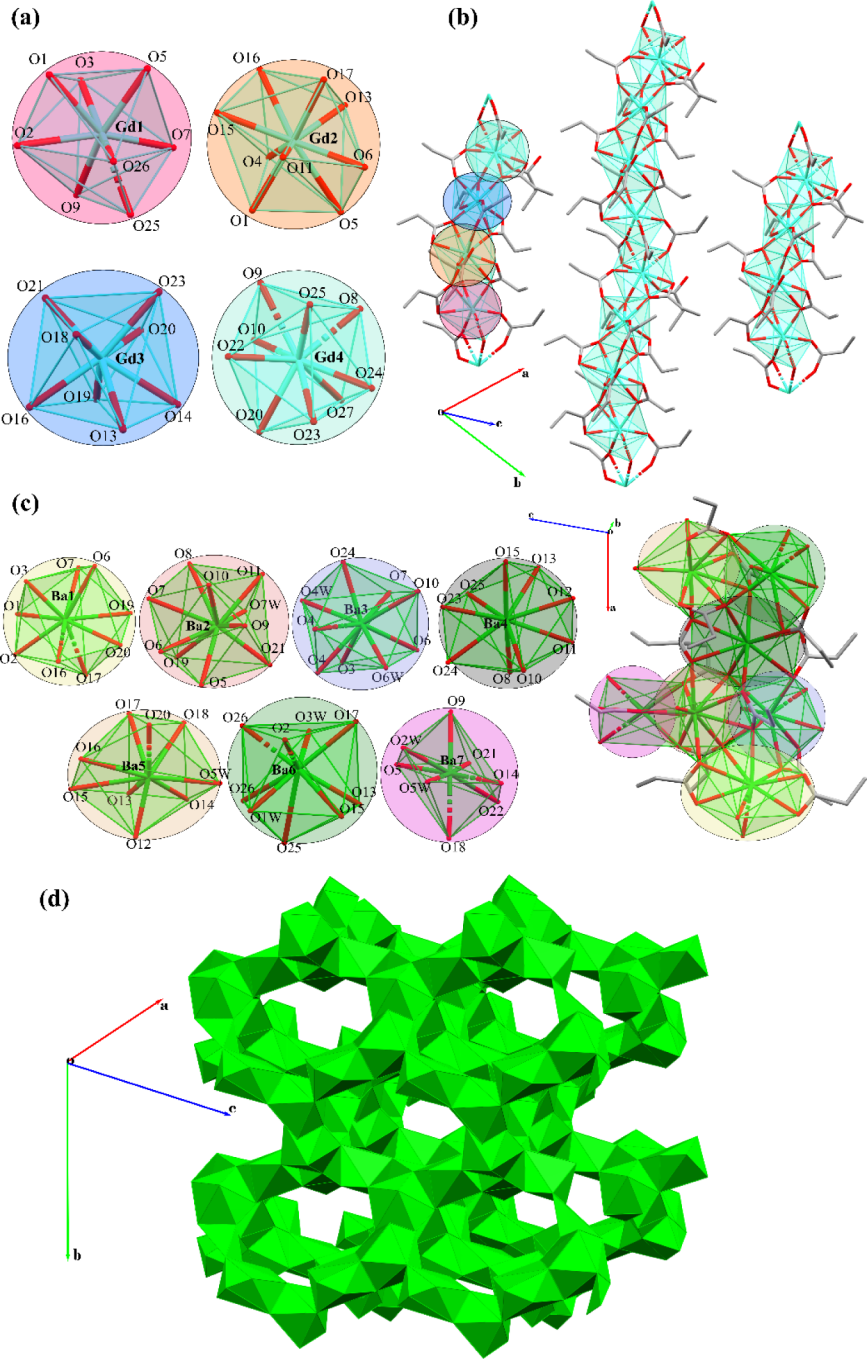
Representation of the (a) sequence of the crystallographically independent Gd(III) centers and (b) one-dimensional polymeric chains of Gd(Prop)_3_. Representation of the (c) seven crystallographically independent Ba(II) centers in Ba(Prop)_2_, which arrange into a distorted honeycomb 3D-lattice (d).

Gadolinium propionate obtained through the synthetic method described in this paper shows two pairs of Gd(III) centers bearing [GdO_8_] and [GdO_9_] cores with, respectively, coordination numbers 8 and 9 (Figure 1a). This results in a structural arrangement of polymeric 1D chains (Figure 1b). No other crystal structure relative to pure gadolinium propionate is reported in the literature. The structure of Ba(Prop)_2_ displays seven crystallographically independent Ba (II) centers (Figure 1c) bearing one [BaO_8_] core, five [BaO_9_] cores, and one [BaO_10_] core with, respectively, coordination numbers 8, 9, and 10. The arrangement of the various geometries of the crystallographically independent Ba (II) centers results in a distorted honeycomb 3D-lattice (Figure 1d). Mos et al. previously described a mixed complex of barium-acetato-propionate,^36^ but the compound described in this manuscript is the first, pure barium propionate structure ever reported. More details regarding the described crystal structures may be found in Section I.

Both single-crystal structures for Y(Prop)_3_ and Sm(Prop)_3_ yield 2D arrangement. There is a previously reported crystal structure of yttrium propionate by Nasui et al.;^60^ alternatively, we provide a new polymorphic structure displaying important differences, as the [YO_9_] core with coordination number 9 is bearing a muffin geometry (S values reported in Table S8). The dimeric unit is formed by two identical Y(III) centers connected to each of seven oxygen atoms derived from propionate groups and two H_2_O molecules (O7 and O8 in Figure S6a). The H_2_O molecules assemble the dimeric units through intramolecular and intermolecular H-bonds (Figure S6b) into a zigzag 2D-sheet arrangement along the *bc* plane (Figure S6c). Information on the types of coordination modes present may be found in Section I.

Only one further crystal structure of samarium propionate can be found in literature,^61^ and it exhibits an anhydrous structure with coordination to propionate ligands and HProp in a one-dimensional arrangement of polymeric chains. Conversely, the compound of samarium reported in this paper displays a [SmO_9_] core with coordination number 9 bearing a muffin geometry (S values reported in Table S9). There are two crystallographically independent Sm(III) centers, with Sm(1) being connected to seven oxygen atoms relative to propionate groups and two H_2_O molecules (O13 and O14 in Figure S6d), while Sm(2) is solely bonded to propionate ligands. The ligands display a mixed bidentate chelating and bridging arrangement, yielding distorted square lattice 2D-sheets along the *ab* plane (Figures S6e and S7). Information on the specific types of coordination modes present in this structure is reported in Section I.

Comparison between the measured PXRD and the pattern calculated for single-crystal structures of Ba(Prop)_3_, Sm(Prop)_3_, and Gd(Prop)_3_ can be found in Figure S8 and for Y(Prop)_3_ in Figure S9, confirming that the crystals selected to solve the crystal structures described here match the synthesized powders. Additionally, the single-crystal structure for Gd(Prop)_3_ is showing a different structure with respect to the one exhibited by the PXRD (Figure S10), and its description may be found in Section I and Figure S11.

### Thermal Analysis of Films of Individual Metal Propionates

The thermal behavior of all of the metal propionate powder products was analyzed by comparing their decomposition pathways in inert conditions (N_2_). All information relative to the decomposition of the metal propionate powders may be found in Section II. The respective proposed mechanisms may be found in Scheme S1, and TG curves with the analysis of evolved gases are shown in Figures S12–S14, S16, S18, and S20. XRD data confirming the desired phase formation following the thermal treatment are displayed in Figures S15 and S19, whereas the dTG and DSC curves are reported in Figure S17.

To evaluate the solubility and compatibility of the metal propionates in the solvent mixture required for the TLAG-CSD process, the decomposition of each metal propionate in the form of films has been analyzed.

Regarding the propionates of copper, yttrium, and barium, previous TG studies have extensively covered how the decomposition of these films is affected by the various types of atmospheres.^37,62 −64^ However, in these previous works, acetates of the three metals were dissolved in excess propionic acid, and the resulting solution was used for the deposition of the films. The use of acetate precursors is especially detrimental when considering the case of barium precursor, as the complete conversion of acetate to propionate is never obtained, even in conditions of excess of propionic acid.^36^ Therefore, the use of these powder precursors would lead to the presence of undesired phases in the REBCO precursor solution, such as mixed carboxylate species containing acetate and propionate groups. This may result in inhomogeneities or crack formation in the REBCO precursor film.^38^ For this reason, the propionates described in this article are first synthesized as powders and, once their purity is ensured, dissolved in the chosen mixture of solvents.

**Figure 2:**
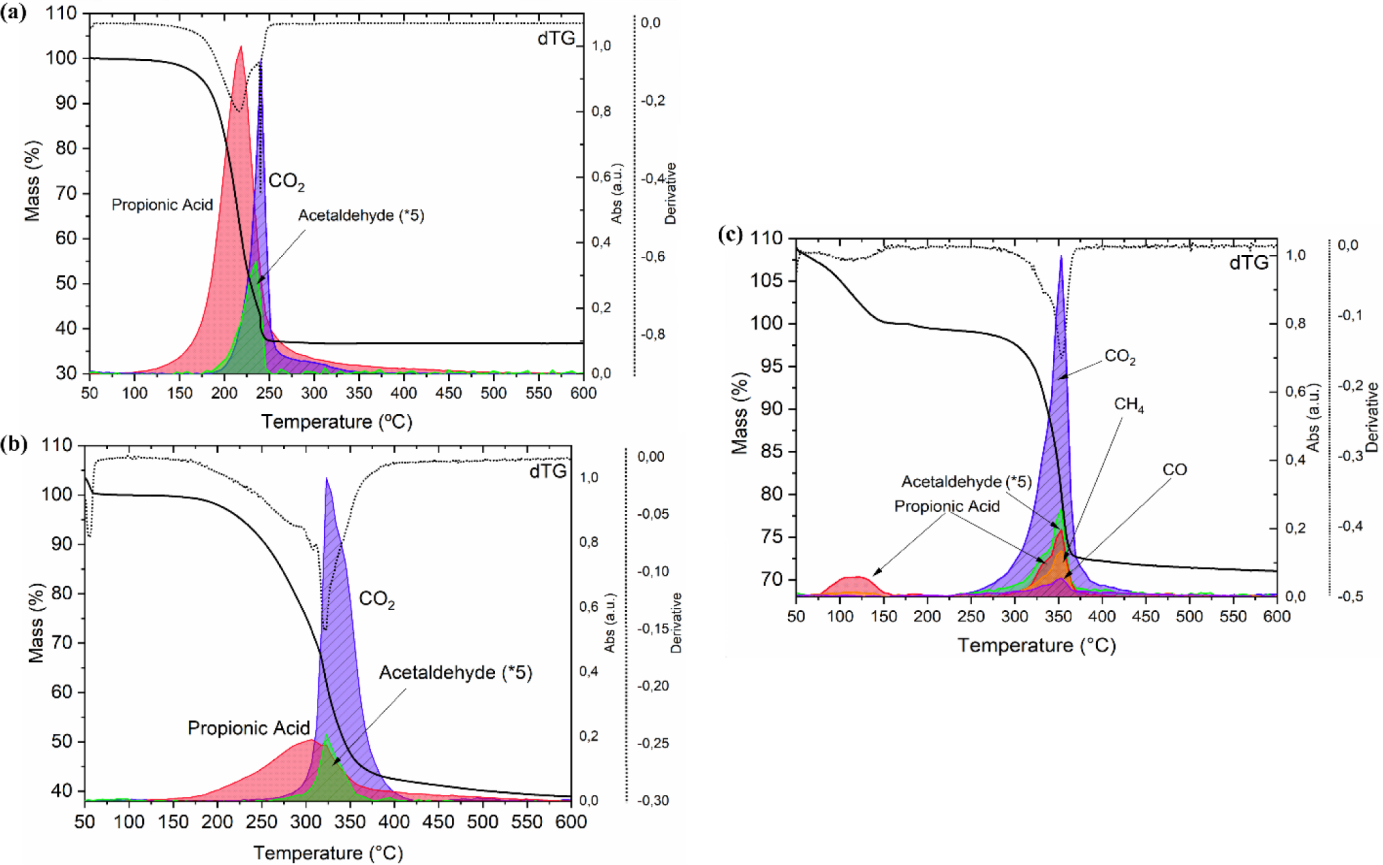
Decomposition of films of (a) Cu(Prop)_2_, (b) Y(Prop)_3_, and (c) Ba(Prop)_2_, in an oxidative atmosphere (dry O_2_). The TG curves are shown as black solid lines, time derivative (dTG) are shown as a black dotted line, and EGA signals are identified for each of the gases released during the process.

We will analyze only the case of an oxidative atmosphere, created by means of a flow of dry O_2_. As shown in Figure 2, in accordance with Rasi et al.,^37,64^ the decomposition in an oxidative atmosphere occurs following an oxidative degradation of the propionate groups (dismutation), with the evolution of propionic acid, CO_2_, and acetaldehyde, in a one-step process to yield CuO from the Cu(Prop)_2_ film, Y_2_O_3_ from the Y(Prop)_3_ film, and BaCO_3_ from the Ba(Prop)_2_ film. For the latter, also methane (CH_4_) and CO evolve, as a result of the decomposition of acetaldehyde and the release of the intermediate species barium oxalate (BaC_2_O_4_), as will be described later. The decomposition temperatures for Cu(Prop)_2_ match the previously reported ones: as shown in the TG profile in Figure 2a, starting as early as 172 °C, the maximum decomposition rate occurs at 220 °C and ends at 250 °C with the full oxidation of Cu(Prop)_2_ to CuO. The case of Y(Prop)_3_, shown in Figure 2b, also shows decomposition temperatures in accordance with the ones reported in the literature for similar systems:^60,64^ decomposition of the propionate groups happens through oxidation around 320 °C, to yield the oxycarbonate species (Y_2_O_2_CO_3_) first, at temperatures ranging between 350 and 480 °C, and finally, at 500 °C, the sesquioxide Y_2_O_3_. For Ba(Prop)_2_, as shown in Figure 2c, the main decomposition step differs from the case of a mixed carboxylate of acetate and propionate referenced in ref,^37^ as it is showing only one main mass loss between 350 and 370 °C, presumably due to the sole presence of propionate groups. In this specific case, in the final region of the decomposition process, the formation of the oxalate species of barium (BaC_2_O_4_) could be observed: BaC_2_O_4_ is known to be an unstable species, which decomposes promptly following its formation, at temperatures between 330 and 350 °C,^37^ to yield BaCO_3_. A series of experiments in which films of a solution of Ba(Prop)_2_ were quenched at different temperatures, displayed in Figure S21b, enabled us to corroborate the presence of barium oxalate as part of the decomposition products of this system. The observed evolution of the CO volatiles at 360 °C, as shown in Figure 2c, is consistent with the complete transformation of the oxalate into BaCO_3_. Although barium oxalate is the intermediate product resulting from a secondary reaction, verification of its presence in the Ba(Prop)_2_ decomposition pathway contributes to an accurate understanding of the system, pivotal for the application proposed in this paper, as well as bearing additional possibilities for its employment in various functional systems. Further information on the decomposition through the formation of a barium oxalate intermediate can be found in Figures S21 and S22 and Section III.

In addition, the formation of the final oxides and BaCO_3_ was confirmed by XRD of samples which underwent the pyrolysis process (step 2 in Scheme 1) described in Experimental Section, to ensure the formation of the desired phases in the same process in which the REBCO nanocrystalline precursor films are prepared. After thermal treatment at 500 °C, the oxides of copper, yttrium, and BaCO_3_ are successfully formed, as displayed in Figure S23. Notice the difference in intensity of the XRD signals, due to the difference in concentration of the solutions of each metal propionate, which will most likely result in different thicknesses of the final film.

**Figure 3:**
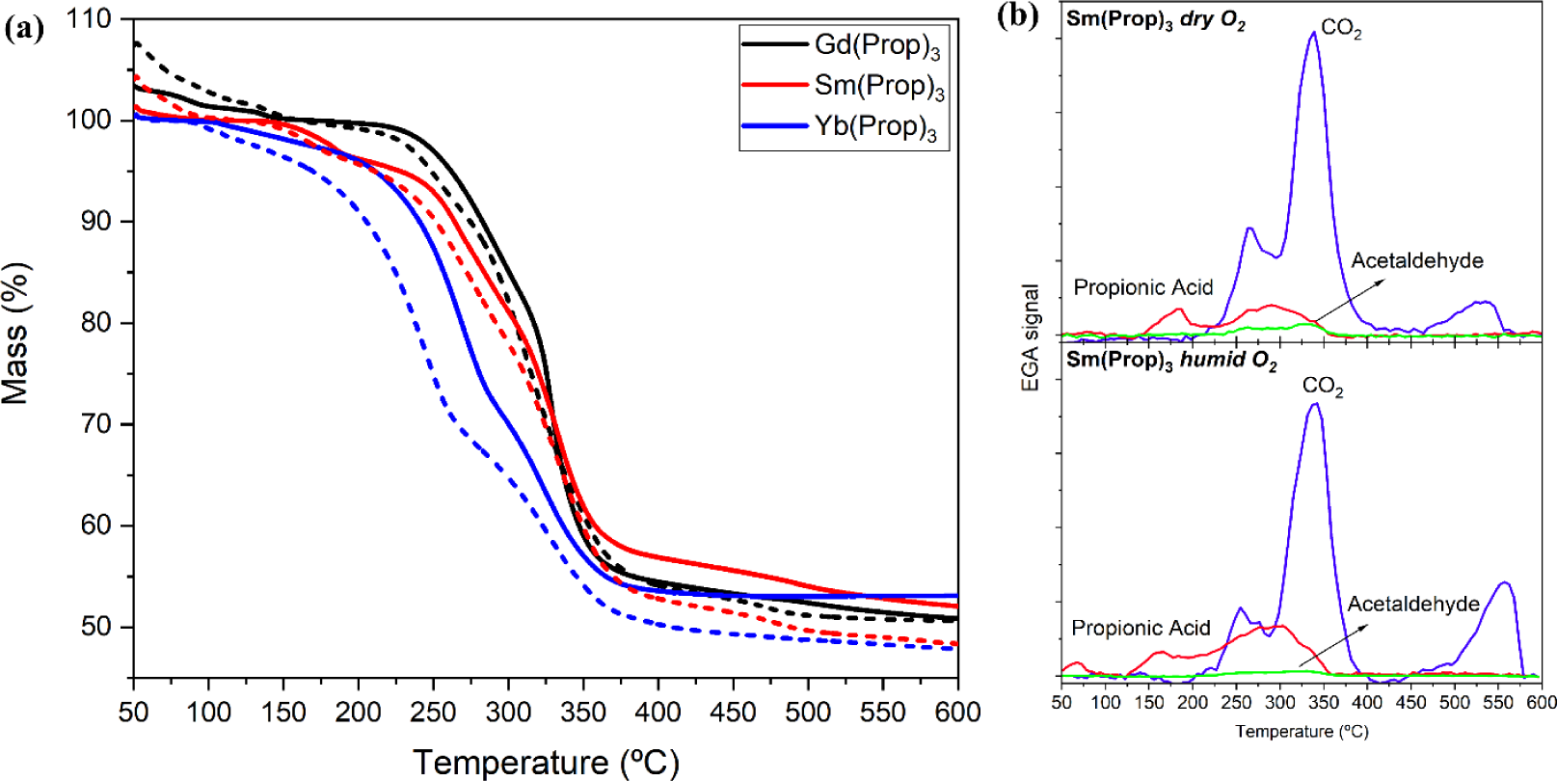
(a) TG curves for Sm(Prop)_3_ (red), Gd(Prop)_3_ (black), and Yb(Prop)_3_ (blue), registered in dry O_2_ (solid lines) and humid O_2_ (dashed lines) flows. (b) Analysis of the evolved gases comparing decomposition in dry and humid oxidative atmospheres during the thermal treatment for Sm(Prop)_3_, here reported as an example. The same analysis for Gd(Prop)_3_ and Yb(Prop)_3_ is reported in Figure S24.

**Scheme 2:**
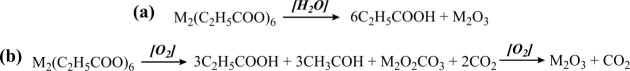
Hydrolysis and Oxidation Mechanism Followed by Films of RE Propionates (M = Y, Sm, Gd, or Yb) When the decomposition is conducted in dry O_2_, only the mechanism in (b) (oxidation of the propionate groups) is occurring, whereas when the TG experiment is performed under a humid O_2_ flux, the hydrolysis mechanism in (a) is favored at lower temperatures, superseded by (b) when higher temperatures are reached.

The thermal analysis of films derived from solutions of RE propionates (RE = Sm, Gd, and Yb) has been conducted in two oxidative atmospheres, dry and humid O_2_, and a comparison of the TG curves is shown in Figure 3a, whereas the EGA from decomposition in both atmospheres is displayed in Figures 3b and S24. The tendency of the TG curves from each RE propionate relative to a dry O_2_ atmosphere (solid lines) is to have a moderately higher decomposition temperature than the ones measured for films exposed to a humid O_2_ flux (dashed lines). The explanation for this behavior lies in the different decomposition mechanism, similar to the one described for Y(Prop)_3_ in,^64^ and displayed in Scheme 2. In dry O_2_, the mechanism governing the decomposition is the same as the cases explained above, an oxidative path that yields propionic acid, acetaldehyde, and CO_2_ in a one-step reaction to form the corresponding oxycarbonate of each RE element, and finally the sesquioxide by elimination of CO_2_. When subjected to a humid O_2_ flux, instead, there is a competition between the hydrolysis and the oxidation mechanisms, the hydrolysis mechanism being favored at lower temperatures. Accordingly, in a humid O_2_ atmosphere, the decomposition starts with the hydrolysis of the RE propionate, which releases propionic acid and sesquioxide of the RE, according to the mechanism shown in Scheme 2a. However, hydrolysis is replaced by the oxidation step when higher temperatures are reached; hence, the evolution of CO_2_ and acetaldehyde, together with propionic acid, yields the oxycarbonate species. Finally, the sesquioxide of each RE is obtained with the release of CO_2_. As a result, the progression of the hydrolysis mechanism is hindered by oxidation, being the latter thermodynamically favored when the temperature is increased. Figures 3b and S24 show the EGA registered for films of Sm(Prop)_3_, Gd(Prop)_3_, and Yb(Prop)_3_ which allows to compare the cases of dry and humid O_2_. The concomitant evolution of propionic acid with CO_2_ and acetaldehyde in the experiments conducted in dry O_2_ for the three RE propionates is a clear confirmation of the oxidative nature of the decomposition, following the reaction scheme shown in Scheme 2b. Conversely, the evolution of propionic acid prior to the release of the other gases implies a first unrelated decomposition step, which is an indication of the hydrolysis reaction.

Corroboration that the sesquioxide of the three RE elements is formed during the pyrolysis process was possible through XRD measurements of films deposited by spin coating and subjected to the thermal treatment described in Experimental Section, and it is displayed in Figure S25.

### Thermal Analysis of Films of Ternary REBCO Precursor Solutions

Finally, after confirmation that the powder precursors are suitable for their application in the preparation of REBCO superconducting films through CSD, we analyzed the rheological properties and the thermal behavior of three REBCO precursor solutions (RE = Sm, Gd, and Yb), resulting from the use of these powder precursors.

The rheology of these solutions has been compared to that of YBCO previously analyzed,^32^ and an overview can be found in Table S13 in Section IV. The values of H_2_O wt % and viscosity of the solutions are all extremely similar; thus, the change of the RE has no direct impact on these solution properties. Nonetheless, the values of contact angles are in the same range, 19.4° and 19.1°, for YBCO and GdBCO precursor solutions, respectively, but lower in the case of SmBCO and YbBCO, being specifically 12.3° and 13.2°. This may lead to a variation during deposition of these solutions, and possibly in the final film thickness. Figure S26 displays the H_2_O wt % as a function of days after solution preparation; as in previous studies conducted on YBCO,^32^ an accurate control of the H_2_O wt % was shown to be pivotal to favor epitaxy of the final film. The REBCO solutions (RE = Sm, Gd, and Yb) show a trend similar to that of the YBCO solution, and we believe this may lead to the same characteristics already observed in the case of yttrium-based solutions.

**Figure 4:**
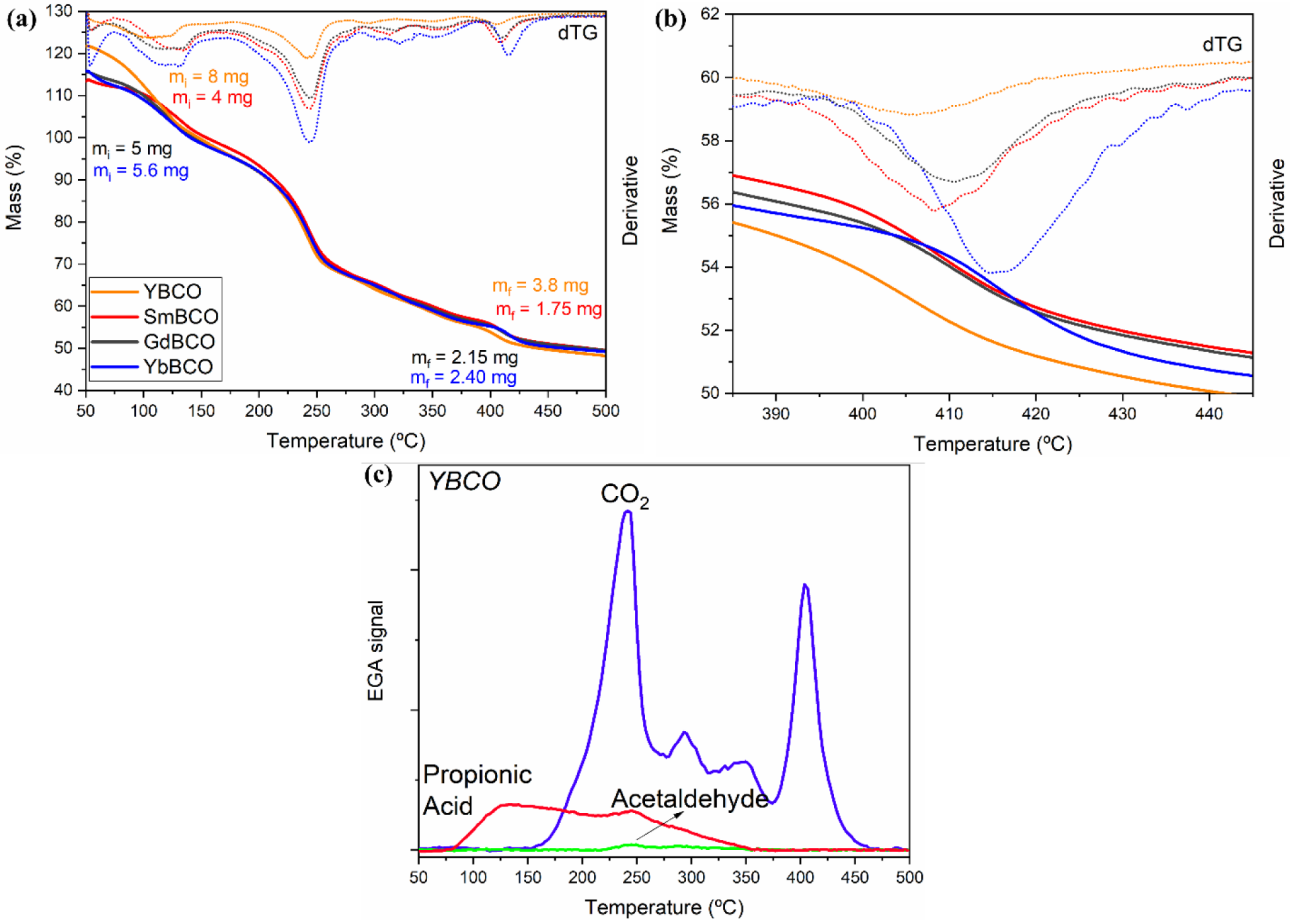
(a) Comparison of TG curves for solutions of YBCO (orange), SmBCO (red), GdBCO (black), and YbBCO (blue), plotted together with the corresponding dTG curves (dotted lines, same colors). Initial and final masses of each solution are shown. Shown in (b) is the final step corresponding to the conversion of the oxycarbonate to the sesquioxide. (c) EGA-FTIR data of the gases released during decomposition for the case of a solution of YBCO. The EGA-FTIR of solutions of SmBCO, GdBCO, and YbBCO is reported in Figure S27.

Before applying these novel REBCO solutions for the CSD of REBCO nanocrystalline precursor films, their thermal decomposition pathway was analyzed, as previously done for the case of YBCO precursor solutions prepared through this method.^32^ A comparison of the TG curves of the three REBCO precursor solutions together with one from a YBCO solution is displayed in Figure 4a. As the films deriving from these solutions have been prepared through drop coating, the difference in mass between the films from these REBCO (RE = Sm, Gd, or Yb) solutions and the YBCO solution is ascribable to more volume used in the latter film deposition. However, this seems to have no important effect on the decomposition pathway other than the initial mass loss coming from the solvent evaporation. In fact, the decomposition temperature ranges are not varying significantly; therefore, it can be assumed that the behavior of these REBCO precursor solutions and the YBCO reference is similar. By analyzing the gas species released through EGA-FTIR, Figure 4c for the YBCO precursor solution and Figures S27 for SmBCO, GdBCO, and YbBCO precursor solutions, respectively, one can notice that the same volatiles are released with an analogous pattern in the evolution with a difference of only tenths of degrees. Therefore, as a general mechanism, we can confirm that the present REBCO precursor solutions follow the same decomposition pathway as the YBCO precursor solution, described in detail in.^30,65^ The initial mass loss below 150 °C, as stated above, is due to the evaporation of the remaining solvent, which, due to the high viscosity of these solutions, has not reached full completion during the drying process. The first meaningful mass loss is in correspondence of 240 °C: the decomposition of Cu(Prop)_2_ begins at approximately 200 °C and is seen to overlap with the start of the RE propionate decomposition. The resulting large decomposition step is concomitant with the highest CO_2_ evolution, with additional release of propionic acid and acetaldehyde. As the experiments are carried out in a humid O_2_ atmosphere, we face the initial hydrolysis and the immediate following oxidation of these precursors to CuO and the RE oxycarbonate species. Subsequently, the next, smaller gas release at 300 °C can be ascribed to the oxidation of the barium precursor, as it has been demonstrated to be stable up to temperatures of 280–300 °C. Finally, the last pronounced gas release, corresponding to a temperature of approximately 400 °C, is relative to two processes: the decomposition of the intermediate barium oxalate (BaC_2_O_4_) to the final BaCO_3_ and the formation of the final sesquioxide of each RE element by release of CO_2_. Note that this last step is shifted to higher temperatures when considering the different RE, following the atomic number in the order Y < Sm < Gd < Yb, especially visible in the dTG curves, shown in Figure 4b. Additionally, the identification of the evolved gases through FTIR also confirms this mechanism and matches the temperature ranges previously described.

Thus, we can conclude that, despite the RE propionates displaying modified crystal structures, the decomposition mechanism of REBCO precursor solutions (step 2 in Scheme 1) is only slightly modified compared with the one presented by a YBCO precursor solution, which has been proven to yield homogeneous nanocrystalline precursor films for the TLAG of epitaxial YBCO superconducting films.

### Microstructural Analysis of REBCO Nanocrystalline Precursor Films

Fundamental to the epitaxial growth of the final REBCO films is the formation of the nanocrystalline precursors of RE_2_O_3_, CuO, and BaCO_3_, homogeneously distributed throughout each layer and with small grain sizes. Therefore, a thorough structural and microstructural analysis through XRD and TEM has been carried out to confirm the nanocrystalline nature of the precursor films derived from the REBCO precursor solutions described in this manuscript, which fulfills the necessary requirements for their application in TLAG-CSD.

Precursor solutions of SmBCO, GdBCO, and YbBCO have been deposited on single crystalline STO substrates via spin coating and subjected to pyrolysis as described in Experimental Section. Following pyrolysis, crack-free films of the three cases are obtained, as displayed in the optical microscopy (OM) image of Figure S28a,c,e, whereas the formation of the desired nanocrystalline precursor phases was confirmed through XRD analyses, displayed in Figure S28b,d,f.

**Figure 5:**
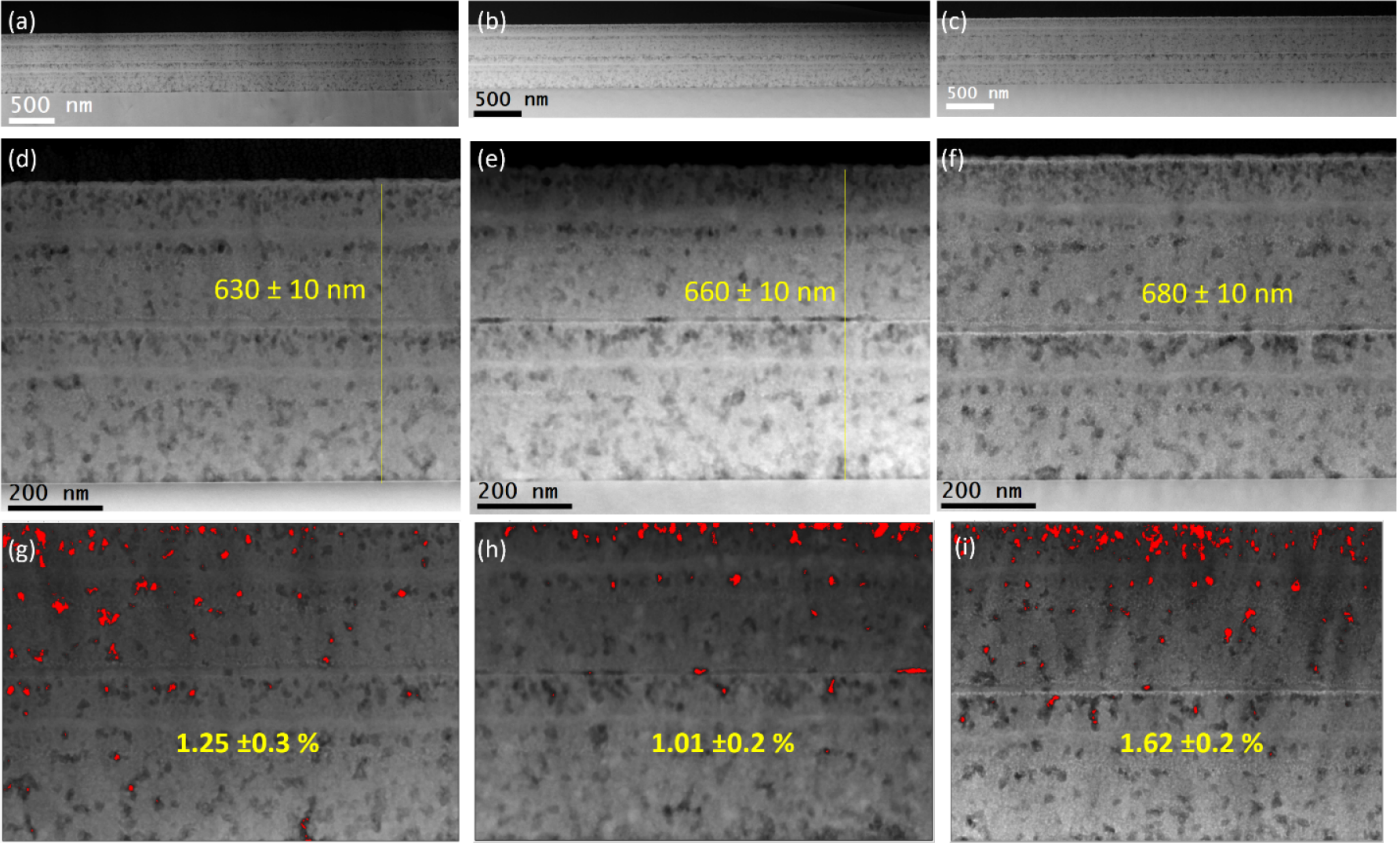
Cross-sectional low-magnification STEM-HAADF images of nanocrystalline precursor thin films deposited using a 1.5 M + 3.4%_v/v_ MEA solution for the cases of (a) SmBCO, (b) GdBCO, and (c) YbBCO. Total thicknesses of the two-layered nanocrystalline precursor films are determined through magnified STEM-HAADF images shown in (d), (e), and (f) for the cases of SmBCO, GdBCO, and YbBCO, respectively. (g–i) Pore density analysis through the use of the software ImageJ, with typical examples of pores displayed in red for their quantification.

A detailed microstructural analysis employing several techniques of electron microscopy reveals that nanocrystalline precursor films (step 2 in Scheme 1) of the three REBCO precursor solutions considered in this paper exhibit consistent features (Figure 5). Low-magnification cross-sectional HAADF-STEM images exhibit that the three REBCO precursor films deposited using SmBCO, GdBCO, and YbBCO solutions show uniform films with smooth surfaces (Figures 5a–c, respectively). The three cases show similar, elevated thicknesses for films of two layers prepared through repetitive deposition, notably, 630 ± 10, 660 ± 10, and 680 ± 10 nm for SmBCO-, GdBCO-, and YbBCO-based precursor films, respectively (Figure 5d–f). Moreover, the homogeneity during these depositions is preserved, as was observed for the case of YBCO nanocrystalline films,^32^ thus suggesting that several processes of multideposition are possible, facilitating the preparation of thick REBCO nanocrystalline precursor films. Figures 5g–i demonstrate the low porosity of the REBCO precursor films after pyrolysis (step 2 in Scheme 1), determined through the processing of HAADF-STEM images using the software ImageJ: pore density estimations of 1.2 ± 0.3%, 1.0 ± 0.2%, and 1.6 ± 0.2% for the SmBCO, GdBCO, and YbBCO nanocrystalline films, respectively, are considered to be optimal for the TLAG process. These are minimal porosity values, as compared to most precursor films prepared by CSD,^14,32^ and they have been proven to enhance greatly the reactivity of the nanocrystalline precursor phases, specifically for the formation of the transient liquid through the reaction between BaCO_3_ and CuO during the TLAG process.

**Figure 6:**
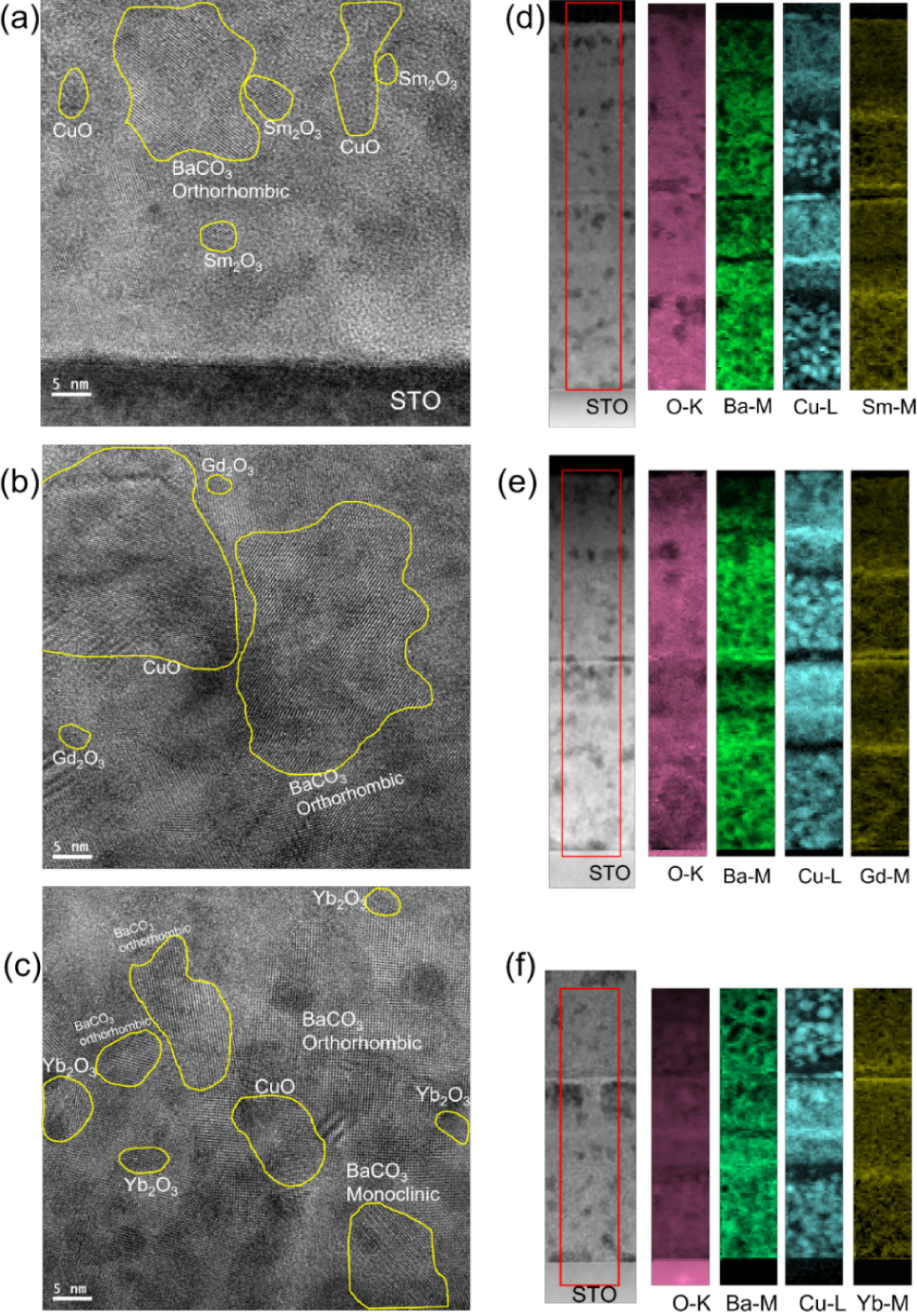
HRTEM images collected from (a) SmBCO, (b) GdBCO, and (c) YbBCO precursor films after pyrolysis (step 2 in Scheme 1): the individual sizes and the presence of all precursor phases in the same area are evidence of a homogeneous distribution of the chemical solutions employed. STEM-HAADF images of (d) SmBCO, (e) GdBCO, and (f) YbBCO two-layer films deriving from 1.5 M + 3.4%_v/v_ MEA solution. Elemental EELS maps of O-K edge (pink), Ba-M edge (light green), Cu-L edge (cyan), and RE (Sm-M, Gd-M, and Yb-M) edge (yellow), from red rectangular regions in (d), (e), and (f), respectively.

A further essential point, which is known to enable the ultrafast growth rate characteristic of TLAG in the case of YBCO, is the small sizes of the nanocrystalline phases: as TLAG is a liquid-assisted process in which the Y_2_O_3_ nanoparticle dissolution in the transient liquid determines the rates of nucleation and growth of the final YBCO phase, a reduced size will significantly enhance the rate of the dissolution and the movement of the yttrium atoms to the nucleation front. In this paper, we demonstrate that the case of REBCO nanocrystalline precursor films based on other RE, for example, Sm, Gd, and Yb, does not differ significantly from what was previously observed for YBCO prepared through this novel fluorine-free chemical solution. By employing high-resolution transmission electron microscopy (HRTEM), we could establish spatial distribution, the crystalline nature, and typical sizes of the nanocrystalline precursor phases. All nanocrystalline phases are identified for the three REBCO systems considered, as shown in Figure 6a–c. Characteristic sizes displayed by BaCO_3_ orthorhombic vary between 15 and 40 nm and between 10 and 15 nm for its monoclinic phase, whereas CuO is formed in an average nanoparticle size of 10–30 nm. The RE oxides in the three cases have extremely reduced diameters, 3–7 nm for Sm_2_O_3_, 3–5 nm for Gd_2_O_3_, and 5–6 nm for Yb_2_O_3_.

Moreover, through electron energy loss spectroscopy (EELS), elemental maps of Cu-L_2,3_, Ba-M_4,5_, and O-K could be acquired along with Sm-M_4,5_, Gd-M_4,5_, and Yb-M_4,5_, which are displayed in Figure 6d–f, respectively. The high homogeneity in the scale of a few nm is reflected in the uniform distribution of the phases throughout the entirety of the films. Thin RE_2_O_3_ and CuO segregations of less than 15 nm thickness are shown, similar to those previously observed in YBCO films;^38^ however, these are not preventing the distribution of these phases consistently in the rest of the film. The homogeneous distribution and the aforementioned typical diameters of the nanocrystalline precursor phases are a confirmation of the robustness of the optimized chemical solution also when extended to the case of REBCO systems (other than Y). The microstructure presented in this section is believed to be the ideal starting point of the TLAG process for the preparation of epitaxial REBCO superconducting films.

### Microstructure and Superconducting Properties of Epitaxial REBCO Films

**Figure 7:**
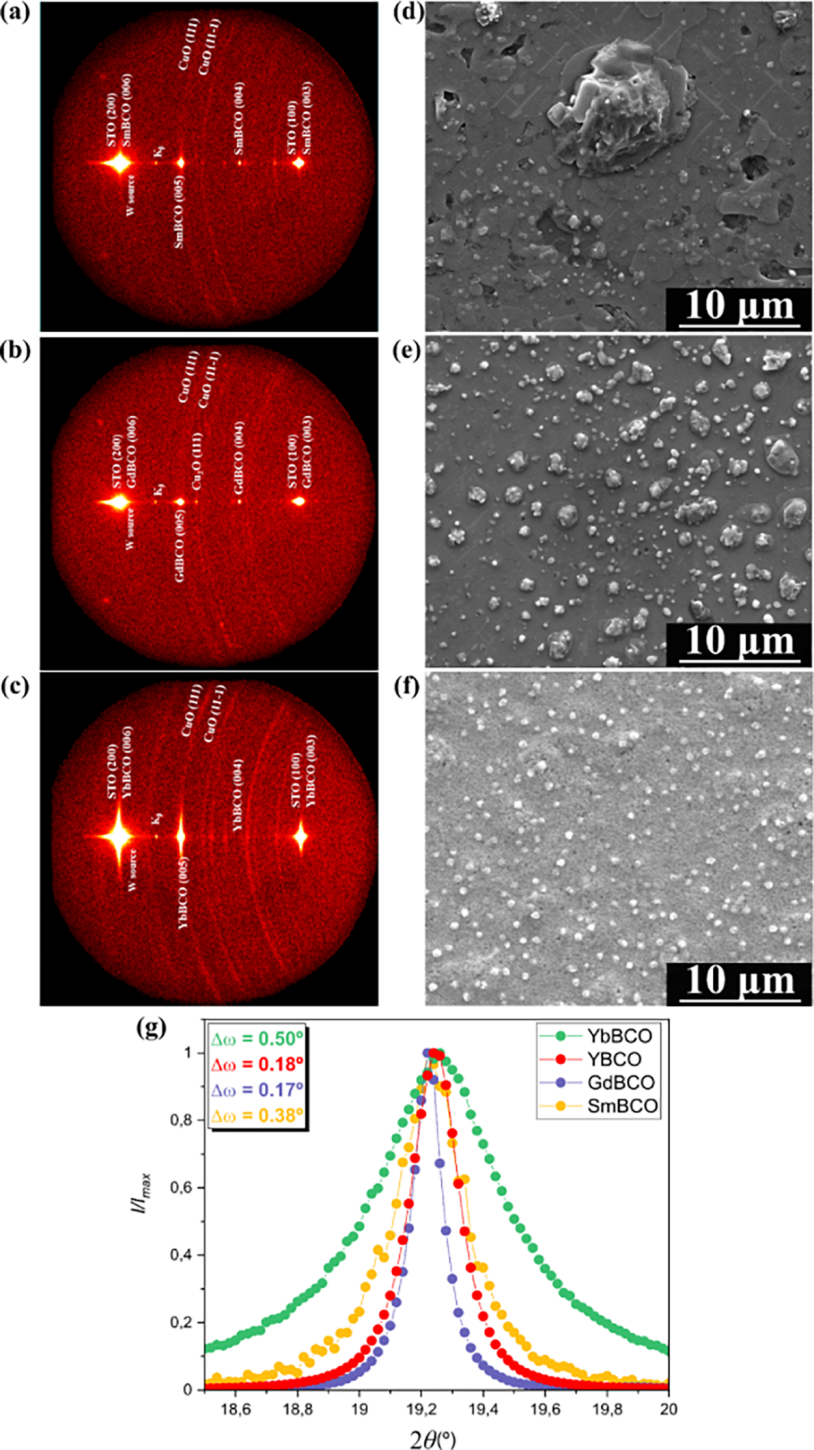
2D-XRD images acquired through GADDS of films (a) SmBCO, (b) GdBCO, and (c) YbBCO grown through TLAG. Identification of the crystalline phases is indicated next to each reflection. SEM images acquired from the (d) SmBCO, (e) GdBCO, and (f) YbBCO films showing flat surface morphology with the ideal characteristic of the excess of copper characteristic of the stoichiometry of this particular liquid composition found as CuO precipitates on the surface. (g) ω-scans of the four REBCO films grown through TLAG, with specific Δω values found in the inset.

Microstructural analyses of TLAG films (after step 5 in Scheme 1) of the three REBCO systems described in this manuscript showed that they have an epitaxial orientation, as confirmed by X-ray diffraction (Figure 7a–c), with optimal surface morphologies (Figure 7d–f), in agreement with the case of the high-performance YBCO films previously reported.^32^ All these films have been prepared with an excess in the initial Cu content to facilitate the achievement of *c*-axis epitaxy; therefore, the final films show the excess of CuO grains in the X-ray diffraction patterns (Figure 7a–c), and in the SEM images, it is confirmed that they have been pushed to the film surface (Figure 7d–f). This confirms that the REBCO films are free of CuO secondary phases embedded in the matrix which would reduce the critical currents.

X-ray diffraction patterns and ω-scan analyses (Figure 7g) additionally allow us to conclude that, although *c*-axis-oriented films have been achieved, YBCO and GdBCO films have a higher texture quality (Δω = 0.18°) than SmBCO and YbBCO films (Δω = 0.38° and 0.50°, respectively).

**Figure 8:**
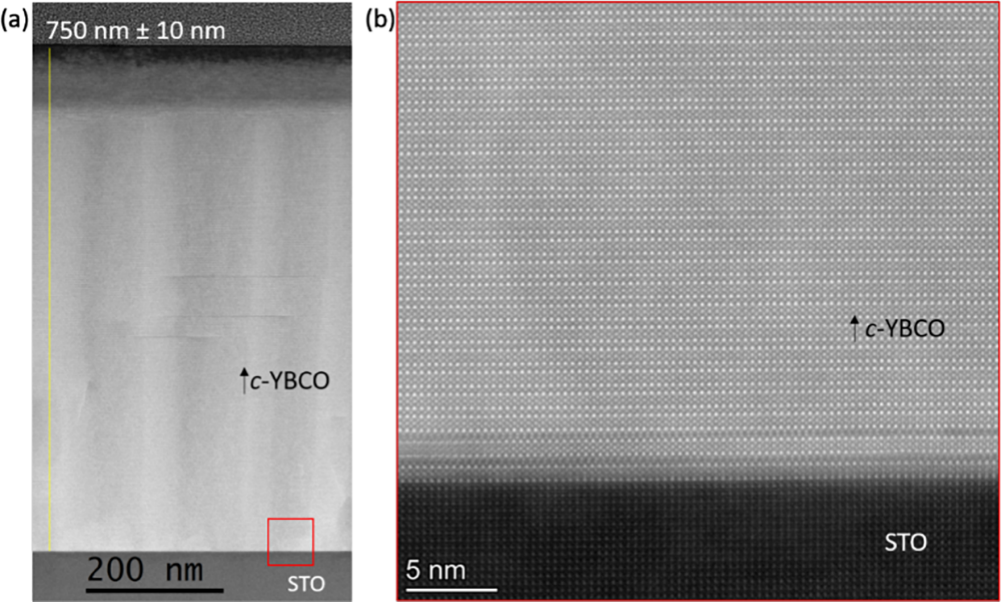
(a) Low-magnification cross-sectional STEM-HAADF image displaying the highly homogeneous growth achieved for an ∼750 nm thick YBCO film grown through TLAG  route. (b) Atomic-resolution STEM-HAADF image showing the highly epitaxial interface of STO substrate to YBCO film.

A low-magnification cross-sectional STEM-HAADF image confirms that high-quality pores-free YBCO film with a smooth surface, displaying a thickness of ∼750 nm, has been obtained on STO single crystalline substrate (Figure 8a). Moreover, the atomic-resolution STEM-HAADF image displayed in Figure 8b exhibits a sharp epitaxial interface between the STO substrate and *c*-YBCO.

**Figure 9:**
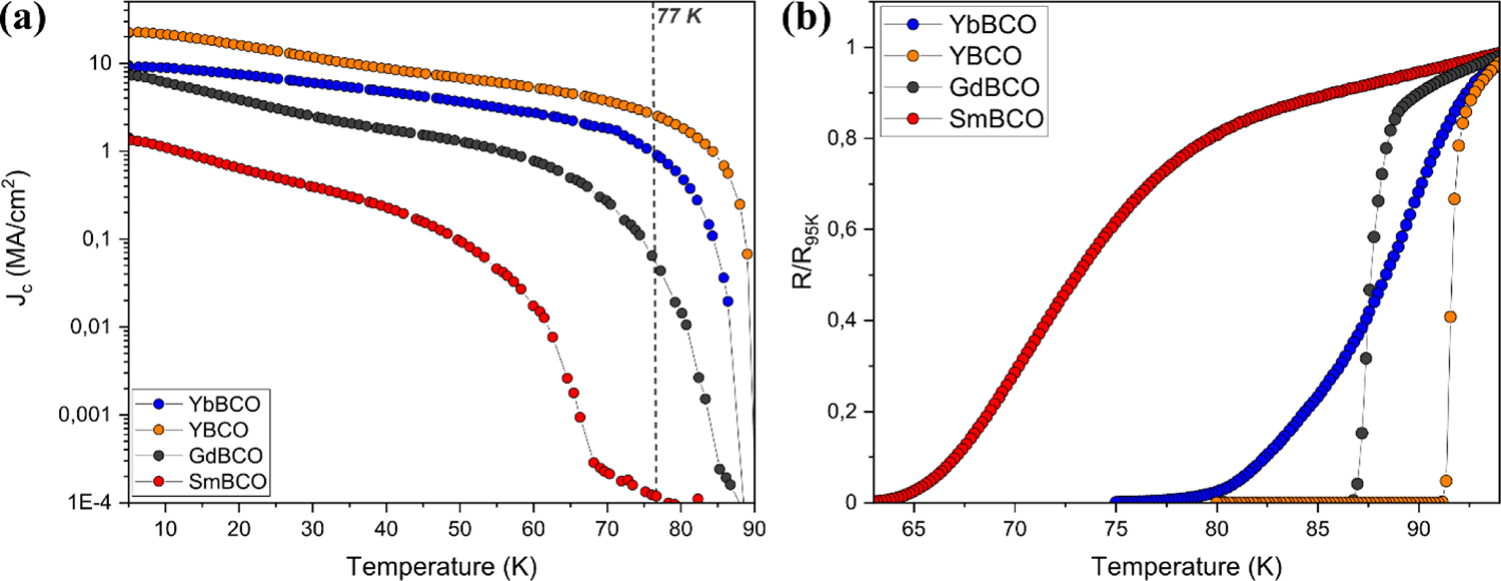
(a) *J*_c_ curves and (b) resistivity curves as a function of temperature for superconducting films of YBCO (orange), SmBCO (red), GdBCO (black), and YbBCO (blue) films grown through TLAG. Resistivity curves are obtained through the van der Pauw method and are normalized to each value at 95 K.

Nonetheless, the achievement of highly performant superconducting YBCO films (*J*_c_ = 2.3 MA/cm^2^ at 77 K and self-field) was the result of a meticulous optimization of the processing parameters in steps 4, 5, and 6 (Scheme 1), including the oxygenation process after growth.^66^ This optimization process has yet to be carried out for the REBCO (RE = Sm, Gd, and Yb) films which were grown only under one specific combination of temperature and  conditions, as specified in Experimental Section. Therefore, growth analyses undertaken for these systems intend to bring a proof-of-concept toward the widening of the TLAG-CSD approach to REBCO systems other than YBCO. As a result, the critical current density (*J*_c_) of these films is in the range of 1–10 MA/cm^2^ at 5 K (23 MA/cm^2^ in YBCO), as displayed in Figure 9a. Resistive and inductive measurements of the superconducting transitions of the new REBCO films are significantly wide with reduced *T*_c_ values (Figures 9b and S29), very likely due to incomplete and inhomogeneous oxygenation,^66−69^ and thus, a fine adjustment of the parameters controlling this process is still needed.

These results are promising toward the realization of high-performance superconducting REBCO films, and we believe a thorough optimization process of the processing parameters of the TLAG growth as well as of the oxygenation process will demonstrate competitive superconducting properties for REBCO films other than YBCO.

## Conclusions

Synthetic routes for a series of metal propionates have been developed, optimized, and characterized in detail, yielding cost-effective and high purity powder precursors of copper, yttrium, barium, samarium, gadolinium, and ytterbium propionates for their use in the CSD of superconducting REBCO films. Crystal structures were obtained for all of the described compounds. Specifically, four novel crystal structures have been obtained for the cases of Ba(Prop)_2_, Y(Prop)_3_, Sm(Prop)_3_, and Gd(Prop)_3_, and their characteristics could be correlated to their chemical properties.

The thermal decomposition pathways for all of the synthesized products have been examined thoroughly and established for each individual metal propionate both for powders in an inert atmosphere and for films in an oxidative atmosphere. The synthesis of pure metal propionates has been shown to be pivotal for consistent thermal decomposition by avoiding the possibility of unwanted product mixtures derived from unreacted species that detrimentally vary the final, expected phase formation.

Once the thermal behavior of the metal propionate powder products was confirmed to be compatible with the TLAG-CSD process, their use as precursors for REBCO metal–organic chemical solutions was investigated. A comparison with a previous study regarding the optimization of YBCO precursor solutions evidenced that variation of the RE has minimal effects on the outcome of the final REBCO nanocrystalline precursor films. Extensive microstructural characterization through various electron microscopy techniques unraveled the highly homogeneous nature of the nanocrystalline precursor films with a uniform distribution of the three phases in each of the REBCO systems under study. These results are a direct consequence of robust and homogeneous chemical solutions, which were confirmed to yield nanocrystalline precursor phases with the desired, optimal features for TLAG through consistent thermal decomposition mechanisms.

The initial examination of the TLAG process on the films of the three REBCO systems considered in this article resulted in a high-quality *c*-axis epitaxy, although a precise tuning of the processing parameters and further optimization of the oxygenation processes are still required to optimize the superconducting properties.

## Supporting Information Available

Full characterization of the metal propionates powder products (FTIR-ATR, SEM, and XRD), single-crystal structure descriptions and relative crystal data, TGA data coupled with EGA-FTIR of individual metal propionates, powders, and films, rheological properties of the REBCO precursor solutions together with TGA-FTIR of these solutions, microstructural characterization of the nanocrystalline precursor films through TEM, and additional data relative to superconducting properties of grown REBCO films (PDF)
Cif files for all the single-crystal structures described in this manuscript (CIF)

## Supplementary Material

am4c11685_si_001.pdf

am4c11685_si_002.cif
