# Supplementary material for: Metal Propionate Solutions for High-Throughput Liquid-Assisted Manufacturing of Superconducting REBa2Cu3O7-δ (RE = Y, Gd, Sm, and Yb) Films
Source: ACS Appl Mater Interfaces. 2024 Sep 26;16(40):54200–15. doi: 10.1021/acsami.4c11685 (PMC11472267; doi:10.1021/acsami.4c11685)
Supplement: Supplementary file 1 — am4c11685_si_001.pdf [file am4c11685_si_001.pdf]

Supporting Information

Metal propionate solutions for high-throughput liquid-assisted manufacturing of superconducting $REBa_2Cu_3O_{7-\delta}$ (RE= Y, Gd, Sm, Yb) films

Lavinia Saltarelli, *[a] Daniel Sanchez-Rodriguez, [b] Kapil Gupta, [a], Aiswarya Kethamkuzhi [a], Jordi Farjas, [b] Elies Molins, [a] Ramón Yañez, [c] Susagna Ricart, [a] Xavier Obradors, [a] Teresa Puig *[a]

[a] *Institut de Ciència de Materials de Barcelona, ICMAB-CSIC, Campus de la UAB, 08193 Bellaterra, Catalonia, Spain*

[b] *GRMT, Department of Physics, University of Girona, E17071-Girona, Catalonia, Spain*

[c] *Departament de Química, Facultat de Ciències, Universitat Autònoma de Barcelona, 08193, Cerdanyola del Vallès, Catalonia, Spain*

*Corresponding authors: Lavinia Saltarelli (lavinia.saltarelli@gmail.com), Prof. Teresa Puig (teresa.puig@icmab.es)

Table of Contents

Section I. Characterization of metal propionates precursors….……………………page 3

Section II. Metal propionates powders decomposition………………………….……page 34

Section III. Metal propionates films decomposition..……………………………….page 44

Section IV. Rheological analysis of REBCO precursor solution…………….…….page 50

Section V. REBCO nanocrystalline films…...…………………………….………........page 52

Section VI. Superconducting properties of epitaxial REBCO films………………..page 53

References…………………………………………………………………………...page 54

Section I. Characterization of metal propionates precursors

$[Cu(Prop)_2]_n$ ($Cu(Prop)_2$). Blue powder, grain size >100 μm after manual crushing, average of 30 μm after mechanical grinding (1 min, 650 rpm). Suitable crystals were obtained by recrystallization in MeOH for 5 days. Yield: 29.88 g (95%). Anal. Calcd. for $C_6H_{10}CuO_4$ (209.688 g·mol^{-1}): C, 34.36; H, 4.81. Found: C, 34.24; H, 4.73. FTIR-ATR (wavenumber, cm^{-1}): 2972 [$\nu_{as}(CH_3)$], 2940 [$\nu_s(CH_3)$], 2919 [$\nu_s(CH_3)$], 2876 [$\nu_{as}(CH_2)$], 1583 [$\nu_{as}(COO^-)$], 1510 [$\delta_{as}(CH_3)$], 1472, 1458, 1417 [$\nu_s(COO^-)$], 1370 [$\delta_{as}(CH_3)$], 1298 [$\omega(CH_2)$], 1242 [$tw(CH_2)$], 1079 [ν (CC_α)], 1006 [$\gamma(CH_3)$], 992 [$\nu_{as}(CC_\alpha)$], 892 [$\nu_s(C_\alpha CO)$], 810 [$\gamma(C_\alpha H_2)$], 753 [$\gamma(CH_2)$], 696, 662 [$\delta(COO^-)$], 595 [$\gamma(COO^-)$] (Figure S1a). $\Delta\nu = \nu_{as}(COO^-) - \nu_s(COO^-) = 166$ cm^{-1}, suggesting a bridging ligand coordination of the propionate ligands[1].

$[Y(Prop)_3{\cdot}(OH_2)]_n$ ($Y(Prop)_3$). White powder, average grain size ~10 μm after manual crushing. Suitable crystals were obtained by recrystallization in MeOH for 8 days. Yield: 26.62 g (96%). Anal. Calcd. for $C_9H_{15}YO_6{\cdot}H_2O$ (326.12 g·mol^{-1}): C, 33.1; H, 5.2. Found: C, 32.99; H, 5.01. FTIR-ATR (wavenumber, cm^{-1}): 3365-3160 [$\nu(OH)$], 2977 [$\nu_{as}(CH_3)$], 2939 [$\nu_s(CH_3)$], 2879 [$\nu_{as}(CH_2)$], 1661, 1558, 1556 [$\nu_{as}(COO^-)$], 1536, 1466 [$\delta_{as}(CH_3)$], 1412 [$\nu_s(COO^-)$], 1375 [$\delta_{as}(CH_3)$], 1287 [$\omega(CH_2)$], 1258 [$tw(CH_2)$], 1077 [ν (CC_α)], 1013 [$\nu_{as}(CC_\alpha)$], 899 [$\nu_s(C_\alpha CO)$], 810 [$\gamma(C_\alpha H_2)$], 766 [$\gamma(CH_2)$], 698, 584 (Figure S1b). $\Delta\nu = \nu_{as}(COO^-) - \nu_s(COO^-) = 146$ cm^{-1}, indicating a mixed case of bidentate chelating and bridging of the propionate ligands.

$[Ce(Prop)_3(OH_2)_3]_n$ ($Ce(Prop)_3$). White powder, grain size between 5 μm and 100 μm after manual crushing. Suitable crystals were obtained by recrystallization in MeOH for 6 days. Yield: 7.40 g (68%). Anal. Calcd. for $C_9H_{15}CeO_6$ (359.33 g·mol^{-1}): C, 30.08; H, 4.20. Found: C, 30.47; H, 4.05. FTIR-ATR (wavenumber, cm^{-1}): 3375 [$\nu(OH)$], 2977 [$\nu_{as}(CH_3)$], 2936 [$\nu_s(CH_3)$], 2874 [$\nu_{as}(CH_2)$], 1708 [$\nu_{as}(C{=}O)$], 1686, 1538 [$\nu_{as}(COO^-)$], 1468 [$\delta_{as}(CH_3)$], 1416 [$\nu_s(COO^-)$], 1369 [$\delta_{as}(CH_3)$], 1285 [$\omega(CH_2)$], 1240 [$tw(CH_2)$], 1116, 1074 [$\nu(CC_\alpha)$], 1008 [$\nu_{as}(CC_\alpha)$], 893 [$\nu_s(C_\alpha CO)$], 813 [$\gamma(C_\alpha H_2)$], 671, 626, 591 (Figure S1d). $\Delta\nu = \nu_{as}(COO^-) - \nu_s(COO^-) = 122$ cm^{-1}, indicating a mixed case of bidentate chelating and bridging of the propionate ligands.

$[Sm_2(Prop)_6(OH_2)_2]_n$ ($Sm(Prop)_3$). Pale yellow powder, grain size between 5 μm and 60 μm after manual crushing. Suitable crystals were obtained by vapour diffusion of acetone in a solution of

Sm(Prop)$_3$ in HProp after 10 days. Yield: 1.72 g (93%). Anal. Calcd. for $C_{21}H_{39}Sm_2O_{16}$ (848.28 g·mol^{-1}): C, 29.73; H, 4.6. Found: C, 30.00; H, 4.54. FTIR-ATR (wavenumber, cm^{-1}): 3346 [ν(OH)], 2973 [ν_{as}(CH$_3$)], 2946 [ν_s(CH$_3$)], 2879 [ν_{as}(CH$_2$)], 1688, 1536 [ν_{as}(COO$^-$)], 1464 [δ_{as}(CH$_3$)], 1410 [ν_s(COO$^-$)], 1379 [δ_{as}(CH$_3$)], 1281 [ω(CH$_2$)], 1245 [tw(CH$_2$)], 1074 [ν (CC$_\alpha$)], 1008 [ν_{as}(CC$_\alpha$)], 894 [ν_s(C$_\alpha$CO)], 813 [γ(C$_\alpha$H$_2$)], 764 [γ(CH$_2$)], 674, 639 (Figure S2a). Δν = ν_{as}(COO$^-$) - ν_s(COO$^-$) = 121 cm^{-1}, indicating a mixed case of bidentate chelating and bridging of the propionate ligands.

[Gd$_2$(Prop)$_6$·2HProp]$_n$ (Gd(Prop)$_3$). White powder, grain size between 10 μm and 100 μm after manual crushing. Suitable crystals were obtained both by recrystallization in MeOH after 5 days and by vapor diffusion of acetone in HProp after 8 days. Yield: 9.76 g (86%). Anal. Calcd. for $C_{21}H_{38}Gd_2O_{15}$ (845.0 g·mol^{-1}): C, 29.86; H, 4.53. Found: C, 29.25; H, 4.1. FTIR-ATR (wavenumber, cm^{-1}): 3585-3389 [ν(OH)], 2984 [ν_{as}(CH$_3$)], 2944 [ν_s(CH$_3$)], 2874 [ν_{as}(CH$_2$)], 1534 [ν_{as}(COO$^-$)], 1465 [δ_{as}(CH$_3$)], 1417 [ν_s(COO$^-$)], 1373 [δ_{as}(CH$_3$)], 1293 [ω(CH$_2$)], 1240 [tw(CH$_2$)], 1081 [ν (CC$_\alpha$)], 1016 [ν_{as}(CC$_\alpha$)], 897 [ν_s(C$_\alpha$CO)], 812 [γ(C$_\alpha$H$_2$)], 765 [γ(CH$_2$)], 680, 641 (Figure S2b). Δν = ν_{as}(COO$^-$) - ν_s(COO$^-$) = 125 cm^{-1}, indicating a mixed case of bidentate chelating and bridging of the propionate ligands.

[Yb(Prop)$_3$·(OH$_2$)]$_n$ (Yb(Prop)$_3$). White powder, grain size between 1 μm and 10 μm after manual crushing. Suitable crystals were obtained by vapour diffusion of acetone in a solution of Sm(Prop)$_3$ in HProp after 14 days. Yield: 1.76 g (79%). Anal. Calcd. for $C_9H_{15}YbO_6\cdot H_2O$ (410.26 g·mol^{-1}): C, 26.3; H, 4.1. Found: C, 25.33; H, 3.89. FTIR-ATR (wavenumber, cm^{-1}): 3366-3187 [ν(OH)], 2980 [ν_{as}(CH$_3$)], 2942 [ν_s(CH$_3$)], 2879 [ν_{as}(CH$_2$)], 1662, 1636, 1555, 1534 [ν_{as}(COO$^-$)], 1471 [δ_{as}(CH$_3$)], 1412 [ν_s(COO$^-$)], 1376 [δ_{as}(CH$_3$)], 1284 [ω(CH$_2$)], 1245 [tw(CH$_2$)], 1078 [ν (CC$_\alpha$)], 1014 [ν_{as}(CC$_\alpha$)], 910 [ν_s(C$_\alpha$CO)], 903 [γ(C$_\alpha$H$_2$)], 810 [γ(CH$_2$)], 771, 676, 614 (Figure S2c). Δν = ν_{as}(COO$^-$) - ν_s(COO$^-$) = 124 cm^{-1}, indicating a mixed case of bidentate chelating and bridging of the propionate ligands.

Synthesis of [Ba$_7$(Prop)$_{14}$(OH$_2$)$_8$]$_n$ (Ba(Prop)$_2$). White powder, average grain size for the final powder product after manual crushing was ~6 μm. Suitable crystals were obtained by recrystallization in MeOH for 14 days. Yield: 25.28 g (88%). Anal. Calcd. for C9H17BaO7 (359.5474 g·mol-1): C, 30.06; H, 4.76. Found: C, 30.00; H, 4.54. FTIR-ATR (wavenumber, cm-

1): 3530-3208 [ν(OH)], 2974 [νas(CH3)], 2941 [νs(CH3)], 2879 [νas(CH2)], 1707 [νas(C=O)], 1650, 1529 [νas(COO-)], 1462 [δas(CH3)], 1413 [νs(COO-)], 1370 [δas(CH3)], 1290 [ω(CH2)], 1246 [tw(CH2)], 1074 [ν (CCα)], 1009 [γ(CH3)], 880 [νas(CCα)], 812 [νs(CαCO)], 606. Δν = νas(COO-) - νs(COO-) = 116 cm-1, indicating a mixed case of bidentate chelating and bridging of the propionate ligands.

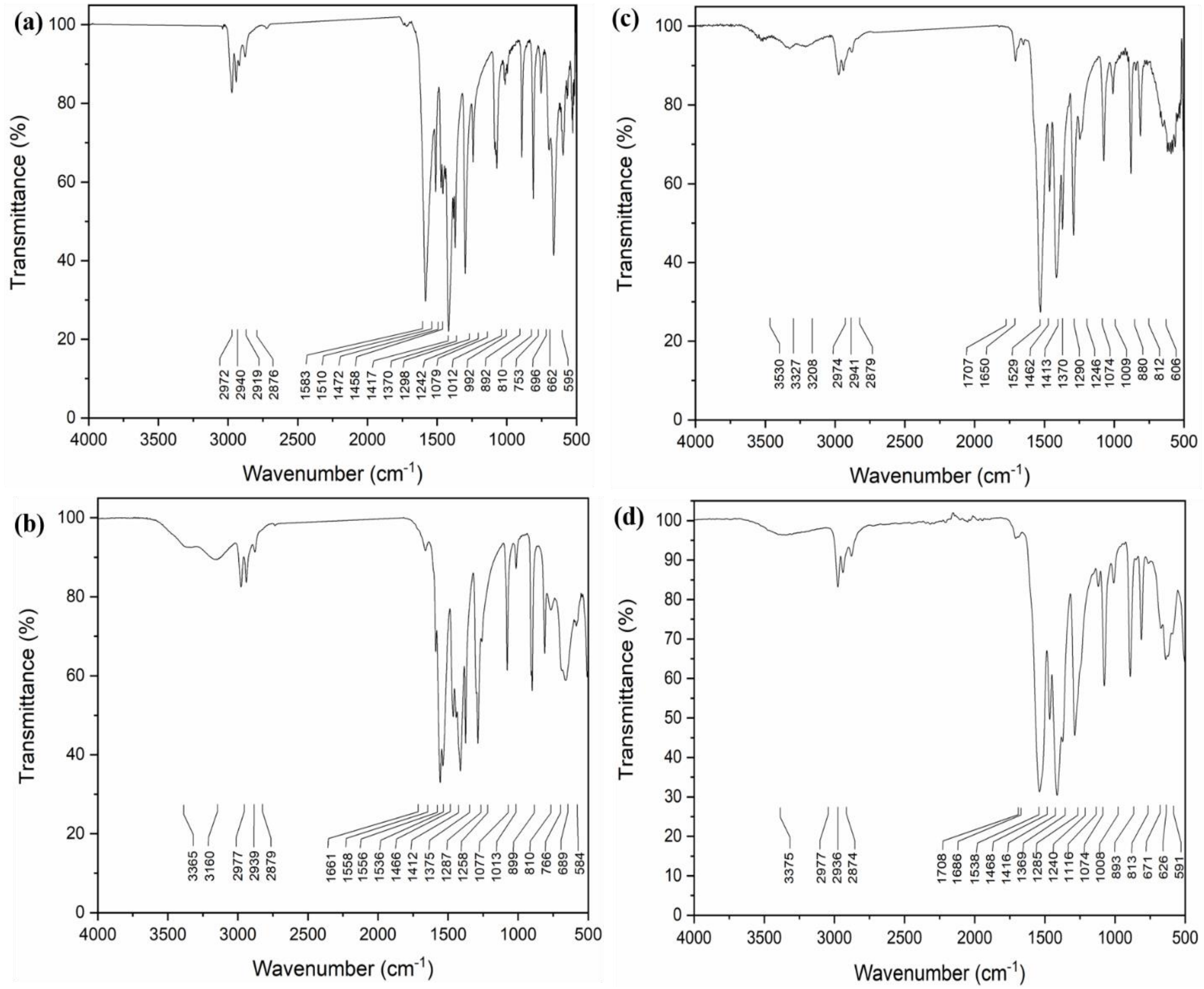

Figure S1. FTIR-ATR spectra measured for (a) Cu(Prop)$_2$, (b) Y(Prop)$_3$, (c) Ba(Prop)$_2$, (d) Ce(Prop)$_3$.

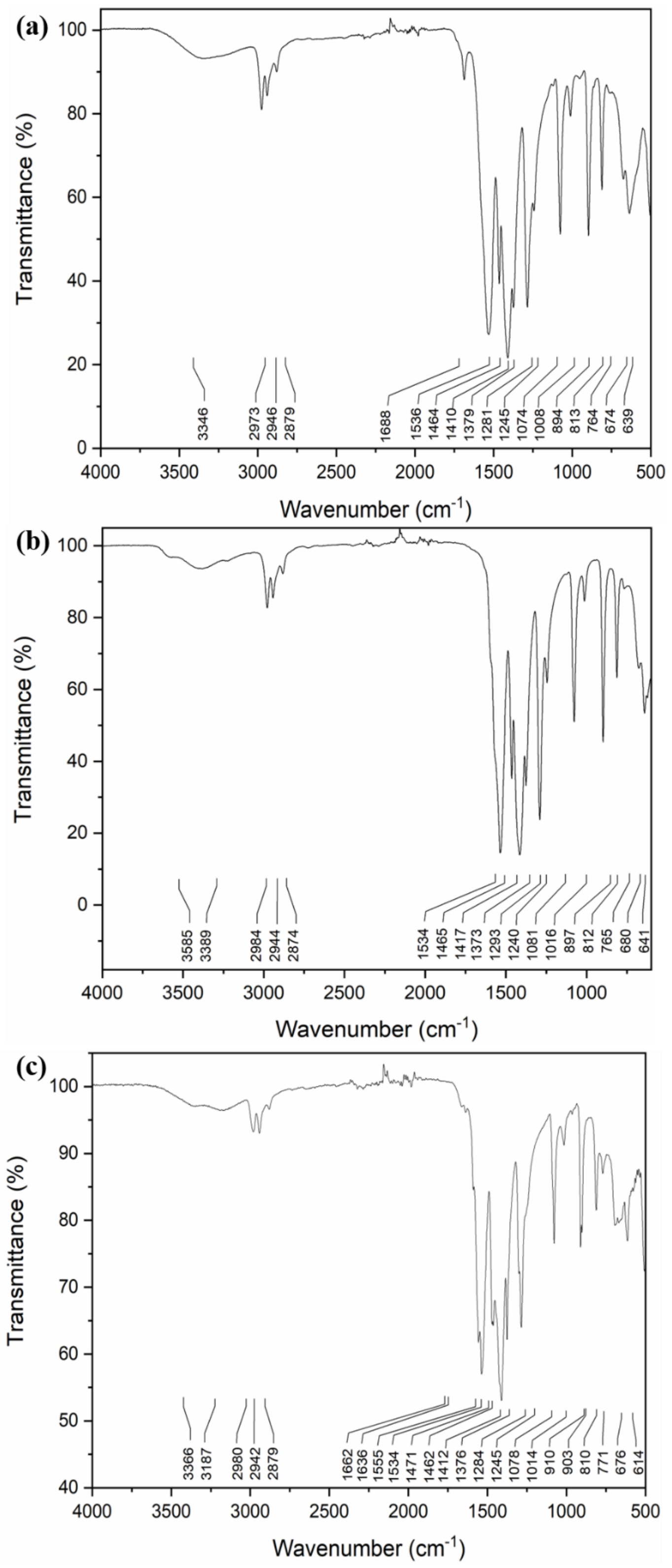

Figure S2. FTIR-ATR spectra measured for (a) $Sm(Prop)_3$, (b) $Gd(Prop)_3$, (c) $Yb(Prop)_3$.

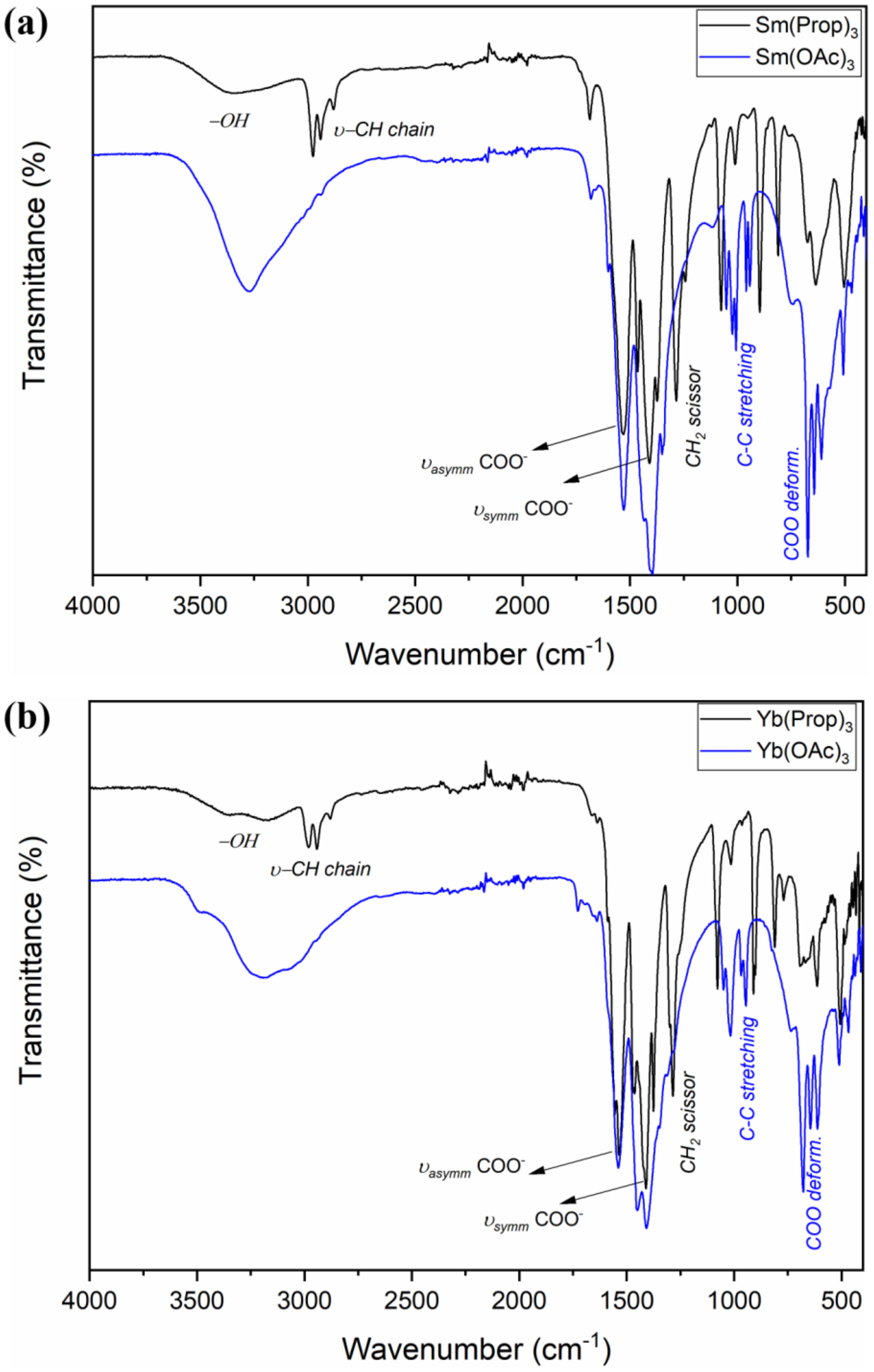

Figure S3. FTIR-ATR spectra measured for (a) $Sm(Prop)_3$, (b) $Yb(Prop)_3$ compared to their respective acetate precursors (blue line). Particularly important is the absence of the *C-C* stretching mode (1009 cm^{-1} for $Sm(OAc)_3$ and 1016 cm^{-1} for $Yb(OAc)_3$) and of the *COO* deformation bands (670 cm^{-1} for $Sm(OAc)_3$ and 675 cm^{-1} for $Yb(OAc)_3$) in the propionate powders, confirming the substitution of acetate to propionate ligands has reached completion entirely.

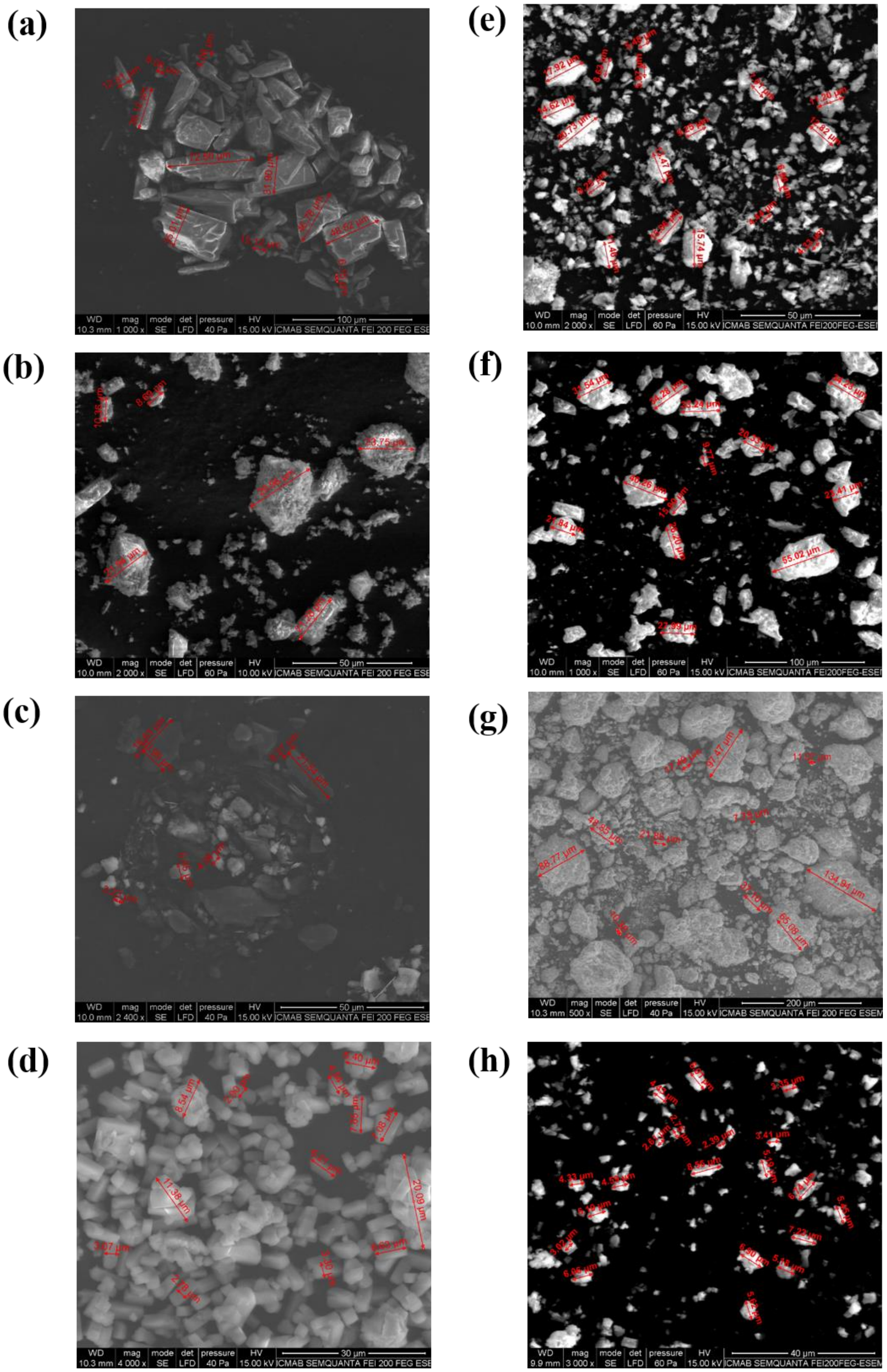

Figure S4. SEM images from the powder precursors with respective grain size measurement of (a) Cu(Prop)$_2$ as synthesized; (b) Cu(Prop)$_2$ after 1 min mechanical grinding; (c) Y(Prop)$_3$; (d) Ba(Prop)$_2$; (e) Ce(Prop)$_3$; (f) Sm(Prop)$_3$; (g) Gd(Prop)$_3$; (h) Yb(Prop)$_3$.

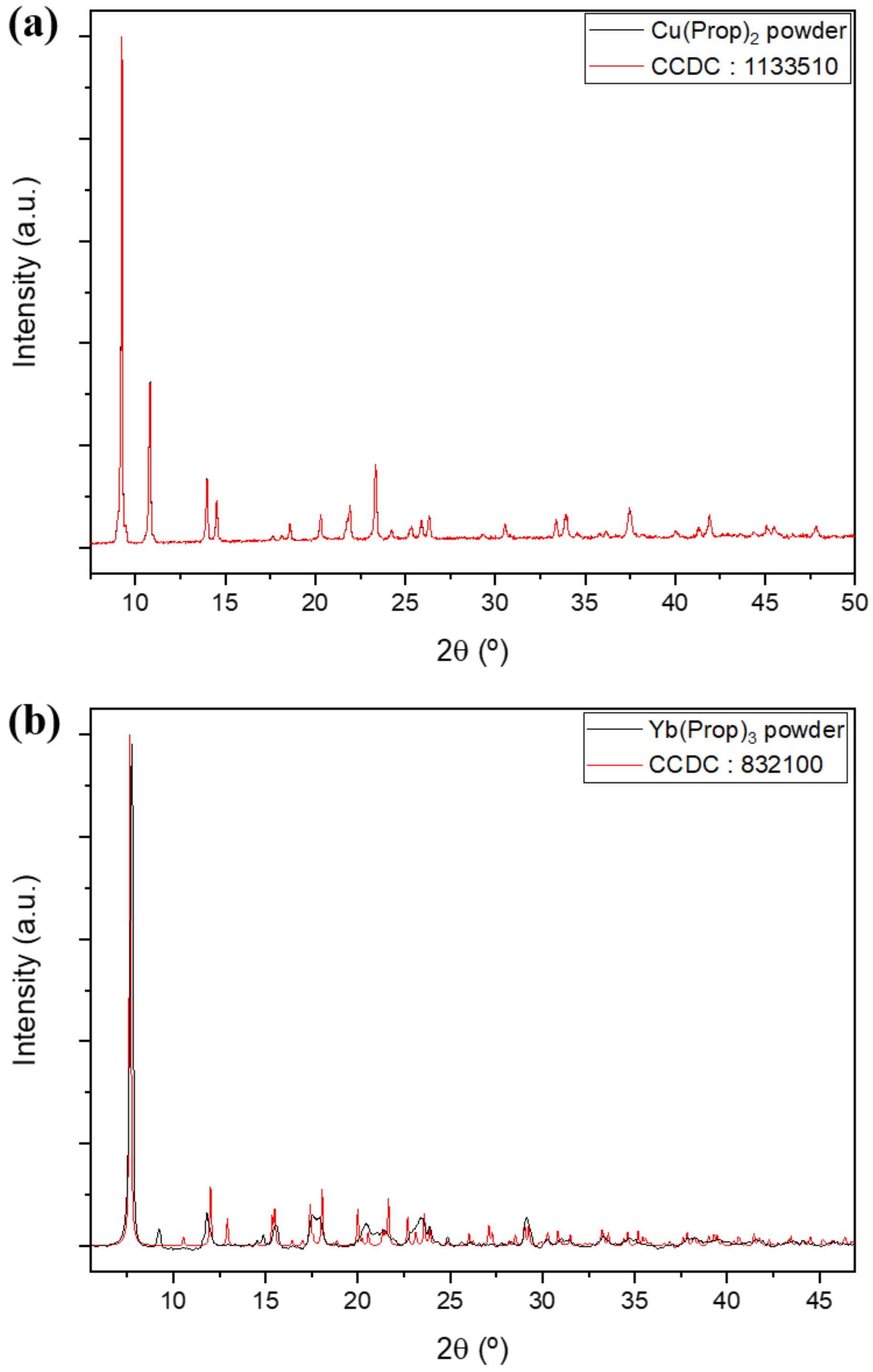

Figure S5. (a) Comparison between XRD of $Cu(Prop)_2$ powder and the one from[2], CCDC: 1133510. (b) Comparison between XRD of $Yb(Prop)_3$ powder and the one from[3], CCDC: 832100.

Table S1. Crystal data and structure refinement for $[Ba_7(Prop)_{14}(OH_2)_8]_n$, $[Sm_2(Prop)_6(OH_2)_2]_n$, $[Gd_2(Prop)_6 \cdot 2HProp]_n$, and $[Y(Prop)_3(OH_2)_2]_n$.

	**$[Ba(Prop)_2] \cdot HProp$**	**$[Sm(Prop)_3(OH_2)]_n$**	**$[Gd(Prop)_3] \cdot HProp$**	**$[Y(Prop)_3(OH_2)_2]_n$**
Empirical formula	$C_{39}H_{79}Ba_7O_{33}$	$C_9H_{17}O_7Sm$	$C_{21}H_{36}Gd_2O_{14}$	$C_9H_{19}O_8Y$
Formula weight	2037.40	387.57	413.50	344.15
Temperature (K)	294(2)	295(2)	295(2)	298(2)
Wavelength (Å)	0.71073	0.71073	0.71073	0.71073
Crystal system	Monoclinic	Triclinic	Triclinic	Monoclinic
Space group	P 21/n	P -1	P -1	P 21/n
Unit cell dimensions				
a (Å)	14.1996(18)	9.620(7) Å	12.794(8)	12.231(3)
b (Å)	16.142(2)	11.594(8)	13.055(9)	14.087(3)
c (Å)	30.554(4)	11.898(9)	20.780(13)	8.8837(18)
α (deg)	90	97.052(14)	75.855(12)	90
β (deg)	94.615(3)	92.512(14)	75.172(14)	97.219(4)
γ (deg)	90	102.805(13)	69.192(10)	90
V (Å^3)	6980.5(16)	1280.9(16)	3091(3)	1518.2(5)
Z	4	4	8	4
D_{calc} (Mg/m^3)	1.939	2.010	1.777	1.506
Absorption coefficient (mm^{-1})	3.961	4.604	4.312	3.866
F(000)	3876	756	1608	704
Crystal size (mm^3)	0.120x0.100x0.060	0.280x0.110x0.09	0.31x0.14x0.12	0.17x0.14x0.09
θ range for data collection (deg)	1.337 to 28.439	1.729 to 28.261	1.029 to 28.414	1.679 to 28.273
hkl ranges	-18<=h<=19<br>-21<=k<=21<br>-40<=l<=40	-12<=h<=12<br>-15<=k<=15<br>-15<=l<=15	-17<=h<=16<br>-17<=k<=17<br>-27<=l<=27	-16<=h<=16<br>-18<=k<=18<br>-11<=l<=11
Reflections collected	140781	34488	85834	32580
Independent reflections	17527	6292	15326	3758
Completeness to θ (%)	99.9	100.0	100.0	100.0

Absorption correction	Semi-empirical from equivalents	Semi-empirical from equivalents	Semi-empirical from equivalents	Semi-empirical from equivalents
Max. and min. transmission	1 and 0.707	1 and 0.31	1 and 0.163	1 and 0.485
Refinement method	Full-matrix least-squares on $\|F\|^2$	Full-matrix least-squares on $\|F\|^2$	Full-matrix least-squares on $\|F\|^2$	Full-matrix least-squares on $\|F\|^2$
Data / restraints/ parameters	17527 / 653 / 767	6292 / 10 / 326	15326 / 86 / 683	3758 / 32 / 182
Goodness-of-fit on $\|F\|^2$	1.164	1.012	0.947	0.945
Final R indices [I>2sigma(I)]	R_1 = 0.1130<br>wR_2 = 0.2679	R_1 = 0.0815<br>wR_2 = 0.1996	R_1 = 0.0711<br>wR_2 = 0.1551	R_1 = 0.0580<br>wR_2 = 0.1661
R indices (all data)	R_1 = 0.1933<br>wR_2 = 0.2986	R_1 = 0.1454<br>wR_2 = 0.2470	R_1 = 0.1775<br>wR_2 = 0.2006	R_1 = 0.0898<br>wR_2 = 0.1799
Extinction coefficient	n/a	0.0042(9)	n/a	n/a
Largest diff. peak and hole (e·Å^{-3})	4.118 and -4.224	8.858 and -3.400	3.049 and -2.008	1.465 and -0.633

Table S2. Bond lengths and torsion angles of Gd(Prop)$_3$.

**Bond lengths (Å)**			
Gd(1)			
Gd(1)-O(7)	2.293(10)	Gd(1)-O(2)	2.436(10)
Gd(1)-O(3)	2.306(9)	Gd(1)-O(26)#1	2.445(11)
Gd(1)-O(9)	2.358(9)	Gd(1)-O(1)	2.494(8)
Gd(1)-O(5)	2.392(10)	Gd(1)-O(25)#1	2.496(8)
Gd(2)			
Gd(2)-O(4)	2.300(10)	Gd(2)-O(17)	2.447(9)
Gd(2)-O(13)	2.379(8)	Gd(2)-O(6)	2.456(11)
Gd(2)-O(11)	2.390(8)	Gd(2)-O(16)	2.525(9)
Gd(2)-O(1)	2.398(8)	Gd(2)-O(5)	2.609(10)
Gd(2)-O(15)	2.437(9)		
Gd(3)			
Gd(3)-O(21)	2.303(9)	Gd(3)-O(14)	2.402(10)
Gd(3)-O(18)	2.321(10)	Gd(3)-O(19)	2.424(11)
Gd(3)-O(16)	2.350(9)	Gd(3)-O(20)	2.483(8)

Gd(3)-O(23)	2.371(10)	Gd(3)-O(13)	2.500(8)

Gd(4)

Gd(4)-O(22)	2.329(11)	Gd(4)-O(24)	2.456(11)
Gd(4)-O(27)	2.384(9)	Gd(4)-O(9)#2	2.539(9)
Gd(4)-O(20)	2.392(8)	Gd(4)-O(23)	2.648(11)
Gd(4)-O(25)	2.400(8)		
Gd(4)-O(10)#2	2.429(10)		
Gd(4)-O(8)#2	2.449(8)		

Bond angles (deg)

Gd(1)

O(7)-Gd(1)-O(3)	87.6(4)	O(2)-Gd(1)-O(26)#1	79.2(4)
O(7)-Gd(1)-O(9)	79.3(4)	O(7)-Gd(1)-O(1)	147.2(3)
O(3)-Gd(1)-O(9)	85.5(3)	O(3)-Gd(1)-O(1)	77.9(3)
O(7)-Gd(1)-O(5)	82.5(4)	O(9)-Gd(1)-O(1)	127.7(3)
O(3)-Gd(1)-O(5)	86.0(3)	O(5)-Gd(1)-O(1)	67.4(3)
O(9)-Gd(1)-O(5)	160.2(3)	O(2)-Gd(1)-O(1)	53.0(3)
O(7)-Gd(1)-O(2)	158.6(4)	O(26)#1-Gd(1)-O(1)	79.7(3)
O(3)-Gd(1)-O(2)	93.4(4)	O(7)-Gd(1)-O(25)#1	75.0(3)
O(9)-Gd(1)-O(2)	79.5(3)	O(3)-Gd(1)-O(25)#1	152.4(3)
O(5)-Gd(1)-O(2)	118.9(3)	O(9)-Gd(1)-O(25)#1	70.4(3)
O(7)-Gd(1)-O(26)#1	107.5(4)	O(5)-Gd(1)-O(25)#1	112.2(3)
O(3)-Gd(1)-O(26)#1	156.3(3)	O(2)-Gd(1)-O(25)#1	95.3(3)
O(9)-Gd(1)-O(26)#1	114.8(4)	O(26)#1-Gd(1)-O(25)#1	51.4(3)
O(5)-Gd(1)-O(26)#1	78.2(4)	O(1)-Gd(1)-O(25)#1	127.6(3)

Gd(2)

O(4)-Gd(2)-O(13)	74.0(3)	O(1)-Gd(2)-O(6)	98.3(3)
O(4)-Gd(2)-O(11)	144.5(3)	O(15)-Gd(2)-O(6)	149.7(3)
O(13)-Gd(2)-O(11)	141.0(3)	O(17)-Gd(2)-O(6)	72.8(3)
O(4)-Gd(2)-O(1)	73.0(3)	O(4)-Gd(2)-O(16)	73.9(3)
O(13)-Gd(2)-O(1)	138.3(3)	O(13)-Gd(2)-O(16)	69.3(3)
O(11)-Gd(2)-O(1)	73.1(3)	O(11)-Gd(2)-O(16)	117.8(3)
O(4)-Gd(2)-O(15)	83.8(4)	O(1)-Gd(2)-O(16)	123.4(3)
O(13)-Gd(2)-O(15)	120.9(3)	O(15)-Gd(2)-O(16)	51.9(3)
O(11)-Gd(2)-O(15)	80.2(3)	O(17)-Gd(2)-O(16)	72.5(3)
O(1)-Gd(2)-O(15)	79.9(3)	O(6)-Gd(2)-O(16)	138.3(3)
O(4)-Gd(2)-O(17)	141.0(3)	O(4)-Gd(2)-O(5)	78.7(3)
O(13)-Gd(2)-O(17)	76.0(3)	O(13)-Gd(2)-O(5)	83.7(3)
O(11)-Gd(2)-O(17)	71.0(3)	O(11)-Gd(2)-O(5)	96.5(3)
O(1)-Gd(2)-O(17)	143.9(3)	O(1)-Gd(2)-O(5)	65.4(3)
O(15)-Gd(2)-O(17)	90.8(3)	O(15)-Gd(2)-O(5)	144.4(3)
O(4)-Gd(2)-O(6)	124.9(4)	O(17)-Gd(2)-O(5)	122.0(3)

O(13)-Gd(2)-O(6)	80.5(3)	O(6)-Gd(2)-O(5)	50.3(3)
O(11)-Gd(2)-O(6)	70.5(4)	O(16)-Gd(2)-O(5)	145.7(3)

Gd(3)

O(21)-Gd(3)-O(18)	86.3(4)	O(14)-Gd(3)-O(19)	80.5(5)
O(21)-Gd(3)-O(16)	85.6(3)	O(21)-Gd(3)-O(20)	77.6(3)
O(18)-Gd(3)-O(16)	77.2(4)	O(18)-Gd(3)-O(20)	146.3(3)
O(21)-Gd(3)-O(23)	87.1(4)	O(16)-Gd(3)-O(20)	129.8(3)
O(18)-Gd(3)-O(23)	81.9(4)	O(23)-Gd(3)-O(20)	68.1(3)
O(16)-Gd(3)-O(23)	158.2(4)	O(14)-Gd(3)-O(20)	80.1(3)
O(21)-Gd(3)-O(14)	157.2(3)	O(19)-Gd(3)-O(20)	52.9(3)
O(18)-Gd(3)-O(14)	110.4(4)	O(21)-Gd(3)-O(13)	151.7(3)
O(16)-Gd(3)-O(14)	112.6(3)	O(18)-Gd(3)-O(13)	74.5(3)
O(23)-Gd(3)-O(14)	80.4(4)	O(16)-Gd(3)-O(13)	70.1(3)
O(21)-Gd(3)-O(19)	89.7(4)	O(23)-Gd(3)-O(13)	110.0(3)
O(18)-Gd(3)-O(19)	157.5(4)	O(14)-Gd(3)-O(13)	51.0(3)
O(16)-Gd(3)-O(19)	80.4(3)	O(19)-Gd(3)-O(13)	100.1(3)
O(23)-Gd(3)-O(19)	120.1(4)	O(20)-Gd(3)-O(13)	129.2(3)

Gd(4)

O(22)-Gd(4)-O(27)	144.5(4)	O(10)#2-Gd(4)-O(24)	147.7(4)
O(22)-Gd(4)-O(20)	72.2(3)	O(8)#2-Gd(4)-O(24)	73.6(3)
O(27)-Gd(4)-O(20)	73.6(3)	O(22)-Gd(4)-O(9)#2	73.1(3)
O(22)-Gd(4)-O(25)	74.2(4)	O(27)-Gd(4)-O(9)#2	119.2(3)
O(27)-Gd(4)-O(25)	140.7(4)	O(20)-Gd(4)-O(9)#2	122.2(3)
O(20)-Gd(4)-O(25)	138.1(3)	O(25)-Gd(4)-O(9)#2	69.1(3)
O(22)-Gd(4)-O(10)#2	87.0(4)	O(10)#2-Gd(4)-O(9)#2	52.1(3)
O(27)-Gd(4)-O(10)#2	78.6(4)	O(8)#2-Gd(4)-O(9)#2	73.0(3)
O(20)-Gd(4)-O(10)#2	81.4(3)	O(24)-Gd(4)-O(9)#2	138.7(3)
O(25)-Gd(4)-O(10)#2	121.2(3)	O(22)-Gd(4)-O(23)	79.3(4)
O(22)-Gd(4)-O(8)#2	140.8(3)	O(27)-Gd(4)-O(23)	94.9(3)
O(27)-Gd(4)-O(8)#2	71.4(3)	O(20)-Gd(4)-O(23)	65.1(3)
O(20)-Gd(4)-O(8)#2	144.6(3)	O(25)-Gd(4)-O(23)	84.7(3)
O(25)-Gd(4)-O(8)#2	75.8(3)	O(10)#2-Gd(4)-O(23)	146.2(3)
O(10)#2-Gd(4)-O(8)#2	87.4(3)	O(8)#2-Gd(4)-O(23)	122.3(3)
O(22)-Gd(4)-O(24)	124.1(4)	O(24)-Gd(4)-O(23)	49.5(3)
O(27)-Gd(4)-O(24)	70.8(4)	O(9)#2-Gd(4)-O(23)	145.9(3)
O(20)-Gd(4)-O(24)	99.1(3)		
O(25)-Gd(4)-O(24)	79.7(4)		

Symmetry transformations used to generate equivalent atoms:

#1 x-1,y+1,z #2 x+1,y-1,z

Table S3. Bond lengths and torsion angles of Ba(Prop)$_2$.

**Bond lengths (Å)**			
Ba(1)			
Ba(1)-O(3)	2.688(17)	Ba(1)-O(2)	2.844(19)
Ba(1)-O(1)	2.708(18)	Ba(1)-O(19)	2.937(17)
Ba(1)-O(16)#1	2.761(17)	Ba(1)-O(17)#1	2.939(19)
Ba(1)-O(20)	2.820(15)	Ba(1)-O(6)	3.029(18)
Ba(1)-O(7)	2.825(15)		
Ba(2)			
Ba(2)-O(21)	2.634(18)	Ba(2)-O(6)	2.830(18)
Ba(2)-O(7W)	2.779(15)	Ba(2)-O(7)	2.932(15)
Ba(2)-O(19)	2.791(16)	Ba(2)-O(8)	2.936(18)
Ba(2)-O(9)	2.799(18)	Ba(2)-O(10)	2.977(18)
Ba(2)-O(11)	2.802(17)	Ba(2)-O(5)	3.039(18)
Ba(3)			
Ba(3)-O(4)	2.702(19)	Ba(3)-O(6)	2.812(17)
Ba(3)-O(24)	2.710(18)	Ba(3)-O(3)	2.815(18)
Ba(3)-O(4)#2	2.760(18)	Ba(3)-O(10)	2.837(19)
Ba(3)-O(6W)	2.769(16)	Ba(3)-O(4W)	2.968(18)
Ba(3)-O(7)	2.795(15)		
Ba(4)			
Ba(4)-O(8)	2.65(2)	Ba(4)-O(12)	2.916(17)
Ba(4)-O(25)	2.697(18)	Ba(4)-O(13)	2.919(19)
Ba(4)-O(15)	2.720(19)	Ba(4)-O(10)	2.924(18)
Ba(4)-O(23)	2.856(19)	Ba(4)-O(24)	3.036(18)
Ba(4)-O(11)	2.897(18)		
Ba(5)			
Ba(5)-O(12)	2.513(18)	Ba(5)-O(14)	2.870(19)
Ba(5)-O(20)#3	2.618(15)	Ba(5)-O(16)	2.889(19)
Ba(5)-O(5W)	2.798(15)	Ba(5)-O(17)	2.907(19)
Ba(5)-O(18)	2.84(2)	Ba(5)-O(15)	3.138(18)
Ba(5)-O(13)	2.846(19)		
Ba(6)			
Ba(6)-O(26)	2.627(19)	Ba(6)-O(1W)	2.880(18)
Ba(6)-O(13)	2.750(18)	Ba(6)-O(26)#4	2.886(19)
Ba(6)-O(3W)	2.788(16)	Ba(6)-O(15)	3.029(18)
Ba(6)-O(2)#3	2.812(19)	Ba(6)-O(25)	3.029(18)
Ba(6)-O(17)	2.843(19)		

Ba(7)

Ba(7)-O(5)	2.664(19)	Ba(7)-O(2W)	2.816(19)
Ba(7)-O(14)#5	2.67(2)	Ba(7)-O(22)	2.82(2)
Ba(7)-O(9)	2.675(18)	Ba(7)-O(21)	2.88(2)
Ba(7)-O(18)#5	2.80(2)	Ba(7)-O(5W)#5	2.928(19)

Bond angles (deg)

Ba(1)

O(3)-Ba(1)-O(1)	73.7(6)	O(20)-Ba(1)-O(19)	43.9(4)
O(3)-Ba(1)-O(16)#1	94.9(5)	O(7)-Ba(1)-O(19)	66.8(4)
O(1)-Ba(1)-O(16)#1	115.0(6)	O(2)-Ba(1)-O(19)	136.2(6)
O(3)-Ba(1)-O(20)	133.0(5)	O(3)-Ba(1)-O(17)#1	157.3(6)
O(1)-Ba(1)-O(20)	153.0(6)	O(1)-Ba(1)-O(17)#1	93.8(6)
O(16)#1-Ba(1)-O(20)	65.6(5)	O(16)#1-Ba(1)-O(17)#1	72.9(5)
O(3)-Ba(1)-O(7)	78.6(5)	O(20)-Ba(1)-O(17)#1	59.9(5)
O(1)-Ba(1)-O(7)	76.3(5)	O(7)-Ba(1)-O(17)#1	117.5(5)
O(16)#1-Ba(1)-O(7)	165.1(5)	O(2)-Ba(1)-O(17)#1	67.6(5)
O(20)-Ba(1)-O(7)	109.2(4)	O(19)-Ba(1)-O(17)#1	72.5(5)
O(3)-Ba(1)-O(2)	91.0(6)	O(3)-Ba(1)-O(6)	64.9(5)
O(1)-Ba(1)-O(2)	42.3(5)	O(1)-Ba(1)-O(6)	120.9(5)
O(16)#1-Ba(1)-O(2)	75.5(6)	O(16)#1-Ba(1)-O(6)	108.9(5)
O(20)-Ba(1)-O(2)	121.1(5)	O(20)-Ba(1)-O(6)	81.1(5)
O(7)-Ba(1)-O(2)	117.6(5)	O(7)-Ba(1)-O(6)	56.2(4)
O(3)-Ba(1)-O(19)	130.2(5)	O(2)-Ba(1)-O(6)	155.6(5)
O(1)-Ba(1)-O(19)	126.7(5)	O(19)-Ba(1)-O(6)	66.3(5)
O(16)#1-Ba(1)-O(19)	109.5(5)	O(17)#1-Ba(1)-O(6)	136.8(5)

Ba(2)

O(21)-Ba(2)-O(7W)	85.3(6)	O(19)-Ba(2)-O(8)	92.8(5)
O(21)-Ba(2)-O(19)	101.3(6)	O(9)-Ba(2)-O(8)	112.0(5)
O(7W)-Ba(2)-O(19)	74.5(5)	O(11)-Ba(2)-O(8)	61.3(5)
O(21)-Ba(2)-O(9)	68.5(6)	O(6)-Ba(2)-O(8)	99.9(5)
O(7W)-Ba(2)-O(9)	133.6(6)	O(7)-Ba(2)-O(8)	44.8(5)
O(19)-Ba(2)-O(9)	145.9(5)	O(21)-Ba(2)-O(10)	113.2(6)
O(21)-Ba(2)-O(11)	90.9(6)	O(7W)-Ba(2)-O(10)	129.8(5)
O(7W)-Ba(2)-O(11)	62.0(5)	O(19)-Ba(2)-O(10)	137.8(5)
O(19)-Ba(2)-O(11)	133.6(5)	O(9)-Ba(2)-O(10)	45.6(5)
O(9)-Ba(2)-O(11)	80.1(5)	O(11)-Ba(2)-O(10)	71.1(5)
O(21)-Ba(2)-O(6)	109.1(6)	O(6)-Ba(2)-O(10)	75.3(5)
O(7W)-Ba(2)-O(6)	144.5(5)	O(7)-Ba(2)-O(10)	73.6(4)
O(19)-Ba(2)-O(6)	71.0(5)	O(8)-Ba(2)-O(10)	68.8(5)
O(9)-Ba(2)-O(6)	81.6(6)	O(21)-Ba(2)-O(5)	66.4(6)
O(11)-Ba(2)-O(6)	145.6(5)	O(7W)-Ba(2)-O(5)	136.7(5)

O(21)-Ba(2)-O(7)	163.9(5)	O(19)-Ba(2)-O(5)	79.5(5)
O(7W)-Ba(2)-O(7)	101.6(5)	O(9)-Ba(2)-O(5)	66.5(5)
O(19)-Ba(2)-O(7)	67.3(5)	O(11)-Ba(2)-O(5)	144.5(5)
O(9)-Ba(2)-O(7)	114.2(5)	O(6)-Ba(2)-O(5)	42.7(5)
O(11)-Ba(2)-O(7)	105.2(5)	O(7)-Ba(2)-O(5)	99.5(5)
O(6)-Ba(2)-O(7)	57.3(5)	O(8)-Ba(2)-O(5)	142.3(5)
O(21)-Ba(2)-O(8)	150.6(6)	O(10)-Ba(2)-O(5)	92.4(5)
O(7W)-Ba(2)-O(8)	73.6(5)		

Ba(3)

O(4)-Ba(3)-O(24)	124.5(6)	O(6W)-Ba(3)-O(3)	102.0(5)
O(4)-Ba(3)-O(4)#2	65.9(7)	O(7)-Ba(3)-O(3)	77.0(5)
O(24)-Ba(3)-O(4)#2	79.6(6)	O(6)-Ba(3)-O(3)	66.4(5)
O(4)-Ba(3)-O(6W)	77.8(6)	O(4)-Ba(3)-O(10)	155.3(6)
O(24)-Ba(3)-O(6W)	131.3(5)	O(24)-Ba(3)-O(10)	72.9(5)
O(4)#2-Ba(3)-O(6W)	71.9(6)	O(4)#2-Ba(3)-O(10)	105.3(6)
O(4)-Ba(3)-O(7)	121.2(5)	O(6W)-Ba(3)-O(10)	77.6(5)
O(24)-Ba(3)-O(7)	75.2(5)	O(7)-Ba(3)-O(10)	77.8(5)
O(4)#2-Ba(3)-O(7)	152.4(5)	O(6)-Ba(3)-O(10)	77.8(5)
O(6W)-Ba(3)-O(7)	134.3(5)	O(3)-Ba(3)-O(10)	143.3(5)
O(4)-Ba(3)-O(6)	98.0(5)	O(4)-Ba(3)-O(4W)	71.7(6)
O(24)-Ba(3)-O(6)	129.8(5)	O(24)-Ba(3)-O(4W)	62.4(5)
O(4)#2-Ba(3)-O(6)	148.4(6)	O(4)#2-Ba(3)-O(4W)	83.8(6)
O(6W)-Ba(3)-O(6)	78.4(5)	O(6W)-Ba(3)-O(4W)	146.8(5)
O(7)-Ba(3)-O(6)	59.1(5)	O(7)-Ba(3)-O(4W)	74.8(5)
O(4)-Ba(3)-O(3)	45.2(5)	O(6)-Ba(3)-O(4W)	118.0(5)
O(24)-Ba(3)-O(3)	124.7(6)	O(3)-Ba(3)-O(4W)	64.7(5)
O(4)#2-Ba(3)-O(3)	109.4(6)	O(10)-Ba(3)-O(4W)	132.0(5)

Ba(4)

O(8)-Ba(4)-O(25)	88.8(6)	O(23)-Ba(4)-O(13)	117.0(5)
O(8)-Ba(4)-O(15)	171.6(5)	O(11)-Ba(4)-O(13)	74.8(5)
O(25)-Ba(4)-O(15)	86.7(6)	O(12)-Ba(4)-O(13)	57.8(5)
O(8)-Ba(4)-O(23)	115.5(6)	O(8)-Ba(4)-O(10)	73.4(5)
O(25)-Ba(4)-O(23)	73.1(6)	O(25)-Ba(4)-O(10)	152.7(5)
O(15)-Ba(4)-O(23)	69.9(6)	O(15)-Ba(4)-O(10)	113.2(5)
O(8)-Ba(4)-O(11)	63.5(5)	O(23)-Ba(4)-O(10)	95.6(6)
O(25)-Ba(4)-O(11)	120.2(6)	O(11)-Ba(4)-O(10)	70.6(5)
O(15)-Ba(4)-O(11)	113.1(5)	O(12)-Ba(4)-O(10)	82.0(5)
O(23)-Ba(4)-O(11)	166.0(6)	O(13)-Ba(4)-O(10)	139.0(5)
O(8)-Ba(4)-O(12)	108.9(5)	O(8)-Ba(4)-O(24)	70.4(5)
O(25)-Ba(4)-O(12)	124.1(6)	O(25)-Ba(4)-O(24)	87.6(5)
O(15)-Ba(4)-O(12)	68.2(5)	O(15)-Ba(4)-O(24)	116.4(5)
O(23)-Ba(4)-O(12)	132.8(6)	O(23)-Ba(4)-O(24)	48.1(5)
O(11)-Ba(4)-O(12)	45.5(5)	O(11)-Ba(4)-O(24)	124.0(5)

O(8)-Ba(4)-O(13)	109.6(5)	O(12)-Ba(4)-O(24)	148.2(5)
O(25)-Ba(4)-O(13)	66.3(5)	O(13)-Ba(4)-O(24)	153.7(5)
O(15)-Ba(4)-O(13)	62.0(5)	O(10)-Ba(4)-O(24)	67.2(5)

Ba(5)

O(12)-Ba(5)-O(20)#3	152.1(5)	O(18)-Ba(5)-O(16)	112.5(6)
O(12)-Ba(5)-O(5W)	98.3(5)	O(13)-Ba(5)-O(16)	98.0(5)
O(20)#3-Ba(5)-O(5W)	87.6(5)	O(14)-Ba(5)-O(16)	145.0(6)
O(12)-Ba(5)-O(18)	139.2(6)	O(12)-Ba(5)-O(17)	135.8(5)
O(20)#3-Ba(5)-O(18)	68.5(6)	O(20)#3-Ba(5)-O(17)	62.5(5)
O(5W)-Ba(5)-O(18)	71.5(5)	O(5W)-Ba(5)-O(17)	114.0(5)
O(12)-Ba(5)-O(13)	63.2(5)	O(18)-Ba(5)-O(17)	43.6(6)
O(20)#3-Ba(5)-O(13)	138.3(5)	O(13)-Ba(5)-O(17)	76.0(5)
O(5W)-Ba(5)-O(13)	114.2(5)	O(14)-Ba(5)-O(17)	94.9(6)
O(18)-Ba(5)-O(13)	84.7(6)	O(16)-Ba(5)-O(17)	71.6(5)
O(12)-Ba(5)-O(14)	69.8(6)	O(12)-Ba(5)-O(15)	67.0(5)
O(20)#3-Ba(5)-O(14)	136.4(6)	O(20)#3-Ba(5)-O(15)	106.6(5)
O(5W)-Ba(5)-O(14)	67.3(5)	O(5W)-Ba(5)-O(15)	165.1(5)
O(18)-Ba(5)-O(14)	69.9(6)	O(18)-Ba(5)-O(15)	117.7(5)
O(13)-Ba(5)-O(14)	47.0(5)	O(13)-Ba(5)-O(15)	57.9(5)
O(12)-Ba(5)-O(16)	97.1(5)	O(14)-Ba(5)-O(15)	103.7(5)
O(20)#3-Ba(5)-O(16)	66.4(5)	O(16)-Ba(5)-O(15)	42.7(5)
O(5W)-Ba(5)-O(16)	147.7(5)	O(17)-Ba(5)-O(15)	77.7(5)

Ba(6)

O(26)-Ba(6)-O(13)	149.7(6)	O(2)#3-Ba(6)-O(26)#4	125.9(6)
O(26)-Ba(6)-O(3W)	69.6(6)	O(17)-Ba(6)-O(26)#4	156.7(6)
O(13)-Ba(6)-O(3W)	81.3(6)	O(1W)-Ba(6)-O(26)#4	72.7(6)
O(26)-Ba(6)-O(2)#3	77.2(6)	O(26)-Ba(6)-O(15)	150.1(6)
O(13)-Ba(6)-O(2)#3	129.3(6)	O(13)-Ba(6)-O(15)	60.2(5)
O(3W)-Ba(6)-O(2)#3	126.2(6)	O(3W)-Ba(6)-O(15)	138.9(5)
O(26)-Ba(6)-O(17)	102.5(6)	O(2)#3-Ba(6)-O(15)	76.1(6)
O(13)-Ba(6)-O(17)	78.6(5)	O(17)-Ba(6)-O(15)	80.5(5)
O(3W)-Ba(6)-O(17)	77.9(6)	O(1W)-Ba(6)-O(15)	67.9(5)
O(2)#3-Ba(6)-O(17)	69.4(5)	O(26)#4-Ba(6)-O(15)	118.4(5)
O(26)-Ba(6)-O(1W)	88.8(6)	O(26)-Ba(6)-O(25)	112.8(5)
O(13)-Ba(6)-O(1W)	114.1(5)	O(13)-Ba(6)-O(25)	64.1(5)
O(3W)-Ba(6)-O(1W)	149.2(6)	O(3W)-Ba(6)-O(25)	101.5(6)
O(2)#3-Ba(6)-O(1W)	65.8(5)	O(2)#3-Ba(6)-O(25)	130.4(6)
O(17)-Ba(6)-O(1W)	129.8(5)	O(17)-Ba(6)-O(25)	142.2(5)
O(26)-Ba(6)-O(26)#4	68.5(6)	O(1W)-Ba(6)-O(25)	66.0(5)
O(13)-Ba(6)-O(26)#4	98.4(6)	O(26)#4-Ba(6)-O(25)	45.1(5)
O(3W)-Ba(6)-O(26)#4	78.8(6)	O(15)-Ba(6)-O(25)	75.7(5)

Ba(7)

O(5)-Ba(7)-O(14)#5	176.3(7)	O(2W)-Ba(7)-O(22)	176.8(7)
O(5)-Ba(7)-O(9)	73.9(5)	O(5)-Ba(7)-O(21)	68.5(6)
O(14)#5-Ba(7)-O(9)	107.9(6)	O(14)#5-Ba(7)-O(21)	115.2(6)
O(5)-Ba(7)-O(18)#5	105.0(6)	O(9)-Ba(7)-O(21)	66.7(5)
O(14)#5-Ba(7)-O(18)#5	73.4(6)	O(18)#5-Ba(7)-O(21)	111.8(6)
O(9)-Ba(7)-O(18)#5	178.3(7)	O(2W)-Ba(7)-O(21)	136.6(6)
O(5)-Ba(7)-O(2W)	77.5(6)	O(22)-Ba(7)-O(21)	46.5(6)
O(14)#5-Ba(7)-O(2W)	99.6(6)	O(5)-Ba(7)-O(5W)#5	108.4(5)
O(9)-Ba(7)-O(2W)	78.8(6)	O(14)#5-Ba(7)-O(5W)#5	68.0(5)
O(18)#5-Ba(7)-O(2W)	102.3(6)	O(9)-Ba(7)-O(5W)#5	111.3(5)
O(5)-Ba(7)-O(22)	104.4(7)	O(18)#5-Ba(7)-O(5W)#5	70.2(5)
O(14)#5-Ba(7)-O(22)	78.5(7)	O(2W)-Ba(7)-O(5W)#5	40.1(5)
O(9)-Ba(7)-O(22)	104.2(7)	O(22)-Ba(7)-O(5W)#5	136.8(5)
O(18)#5-Ba(7)-O(22)	74.8(7)	O(21)-Ba(7)-O(5W)#5	176.5(5)

Symmetry transformations used to generate equivalent atoms:

#1 x+1,y,z #2 -x+1,-y,-z #3 x-1,y,z #4 -x,-y+1,-z

#5 -x+1/2,y-1/2,-z+1/2 #6 -x+1/2,y+1/2,-z+1/2

Table S4. Bond lengths and torsion angles of Y(Prop)$_3$.

**Bond lengths (Å)**			
Y(1)			
Y(1)-O(8)	2.319(4)	Y(1)-O(3)	2.418(4)
Y(1)-O(1)	2.342(3)	Y(1)-O(5)	2.459(4)
Y(1)-O(7)	2.351(4)	Y(1)-O(2)#1	2.489(4)
Y(1)-O(6)	2.392(4)	Y(1)-O(1)#1	2.502(4)
Y(1)-O(4)	2.394(5)		

**Bond angles (deg)**			
Y(1)			
O(8)-Y(1)-O(1)	154.06(12)	O(6)-Y(1)-O(5)	53.45(12)
O(8)-Y(1)-O(7)	82.38(15)	O(4)-Y(1)-O(5)	77.53(16)
O(1)-Y(1)-O(7)	79.22(13)	O(3)-Y(1)-O(5)	126.73(16)
O(8)-Y(1)-O(6)	129.47(14)	O(8)-Y(1)-O(2)#1	75.78(15)
O(1)-Y(1)-O(6)	73.58(12)	O(1)-Y(1)-O(2)#1	116.24(13)
O(7)-Y(1)-O(6)	145.80(13)	O(7)-Y(1)-O(2)#1	74.99(15)
O(8)-Y(1)-O(4)	81.72(17)	O(6)-Y(1)-O(2)#1	98.78(14)
O(1)-Y(1)-O(4)	95.24(15)	O(4)-Y(1)-O(2)#1	145.48(15)
O(7)-Y(1)-O(4)	127.70(15)	O(3)-Y(1)-O(2)#	140.43(14)
O(6)-Y(1)-O(4)	75.83(15)	O(5)-Y(1)-O(2)#1	72.42(16)

O(8)-Y(1)-O(3)	75.87(14)	O(8)-Y(1)-O(1)#1	126.05(15)
O(1)-Y(1)-O(3)	81.65(12)	O(1)-Y(1)-O(1)#1	65.29(13)
O(7)-Y(1)-O(3)	74.38(15)	O(7)-Y(1)-O(1)#1	73.57(13)
O(6)-Y(1)-O(3)	120.57(14)	O(6)-Y(1)-O(1)#1	76.40(12)
O(4)-Y(1)-O(3)	53.46(15)	O(4)-Y(1)-O(1)#1	149.72(13)
O(8)-Y(1)-O(5)	77.89(13)	O(3)-Y(1)-O(1)#1	137.32(13)
O(1)-Y(1)-O(5)	126.83(12)	O(5)-Y(1)-O(1)#1	95.35(14)
O(7)-Y(1)-O(5)	145.11(15)	O(2)#1-Y(1)-O(1)#1	51.78(11)

Symmetry transformations used to generate equivalent atoms:

#1 -x+1,-y+1,-z+1

Table S5. Bond lengths and torsion angles of $Sm(Prop)_3$.

**Bond lengths (Å)**			
Sm(1)			
Sm(1)-O(5)	2.338(10)	Sm(1)-O(4)#2	2.475(11)
Sm(1)-O(3)	2.379(10)	Sm(1)-O(3)#2	2.485(10)
Sm(1)-O(1)	2.400(10)	Sm(1)-O(13)	2.501(10)
Sm(1)-O(14)	2.437(9)	Sm(1)-O(11)#1	2.573(10)
Sm(1)-O(12)#1	2.448(12)		
Sm(2)			
Sm(2)-O(9)	2.369(9)	Sm(2)-O(2)#1	2.471(11)
Sm(2)-O(11)	2.369(10)	Sm(2)-O(1)#1	2.516(10)
Sm(2)-O(8)	2.401(10)	Sm(2)-O(7)	2.537(10)
Sm(2)-O(6)	2.416(9)	Sm(2)-O(9)#3	2.618(10)
Sm(2)-O(10)#3	2.417(11)		

**Bond angles (deg)**			
Sm(1)			
O(5)-Sm(1)-O(3)	75.6(4)	O(14)-Sm(1)-O(3)#2	74.8(3)
O(5)-Sm(1)-O(1)	79.8(3)	O(12)#1-Sm(1)-O(3)#2	116.1(4)
O(3)-Sm(1)-O(1)	148.7(3)	O(4)#2-Sm(1)-O(3)#2	51.2(3)
O(5)-Sm(1)-O(14)	145.8(4)	O(5)-Sm(1)-O(13)	72.6(3)
O(3)-Sm(1)-O(14)	110.3(4)	O(3)-Sm(1)-O(13)	79.0(3)
O(1)-Sm(1)-O(14)	101.0(4)	O(1)-Sm(1)-O(13)	75.3(3)
O(5)-Sm(1)-O(12)#1	138.8(4)	O(14)-Sm(1)-O(13)	141.2(3)
O(3)-Sm(1)-O(12)#1	75.4(3)	O(12)#1-Sm(1)-O(13)	73.7(4)
O(1)-Sm(1)-O(12)#1	113.4(3)	O(4)#2-Sm(1)-O(13)	140.0(4)

O(14)-Sm(1)-O(12)#1	72.7(4)	O(3)#2-Sm(1)-O(13)	139.0(3)
O(5)-Sm(1)-O(4)#2	75.5(4)	O(5)-Sm(1)-O(11)#1	134.9(3)
O(3)-Sm(1)-O(4)#2	115.5(3)	O(3)-Sm(1)-O(11)#1	124.0(3)
O(1)-Sm(1)-O(4)#2	75.8(3)	O(1)-Sm(1)-O(11)#1	64.2(3)
O(14)-Sm(1)-O(4)#2	71.7(4)	O(14)-Sm(1)-O(11)#1	71.1(3)
O(12)#1-Sm(1)-O(4)#2	144.3(4)	O(12)#1-Sm(1)-O(11)#1	50.8(3)
O(5)-Sm(1)-O(3)#2	77.4(3)	O(4)#2-Sm(1)-O(11)#1	117.3(3)
O(3)-Sm(1)-O(3)#2	66.8(4)	O(3)#2-Sm(1)-O(11)#1	145.8(3)
O(1)-Sm(1)-O(3)#2	125.9(3)	O(13)-Sm(1)-O(11)#1	72.8(3)

Sm(2)

O(9)-Sm(2)-O(11)	82.9(3)	O(2)#1-Sm(2)-O(1)#1	51.1(3)
O(9)-Sm(2)-O(8)	78.9(3)	O(9)-Sm(2)-O(7)	128.5(4)
O(11)-Sm(2)-O(8)	156.6(3)	O(11)-Sm(2)-O(7)	148.6(3)
O(9)-Sm(2)-O(6)	152.5(3)	O(8)-Sm(2)-O(7)	52.0(3)
O(11)-Sm(2)-O(6)	78.4(3)	O(6)-Sm(2)-O(7)	71.5(3)
O(8)-Sm(2)-O(6)	123.5(3)	O(10)#3-Sm(2)-O(7)	82.1(4)
O(9)-Sm(2)-O(10)#3	115.5(3)	O(2)#1-Sm(2)-O(7)	71.3(3)
O(11)-Sm(2)-O(10)#3	85.3(4)	O(1)#1-Sm(2)-O(7)	113.9(3)
O(8)-Sm(2)-O(10)#3	89.3(4)	O(9)-Sm(2)-O(9)#3	65.0(4)
O(6)-Sm(2)-O(10)#3	83.0(3)	O(11)-Sm(2)-O(9)#3	81.6(3)
O(9)-Sm(2)-O(2)#1	83.8(4)	O(8)-Sm(2)-O(9)#3	77.4(3)
O(11)-Sm(2)-O(2)#1	116.6(3)	O(6)-Sm(2)-O(9)#3	130.6(3)
O(8)-Sm(2)-O(2)#1	76.0(3)	O(10)#3-Sm(2)-O(9)#3	50.6(3)
O(6)-Sm(2)-O(2)#1	86.8(4)	O(2)#1-Sm(2)-O(9)#3	142.2(3)
O(10)#3-Sm(2)-O(2)#1	153.3(3)	O(1)#1-Sm(2)-O(9)#3	133.6(3)
O(9)-Sm(2)-O(1)#1	78.7(3)	O(7)-Sm(2)-O(9)#3	111.2(3)
O(11)-Sm(2)-O(1)#1	65.5(3)		
O(8)-Sm(2)-O(1)#1	124.1(4)		
O(6)-Sm(2)-O(1)#1	75.3(3)		
O(10)#3-Sm(2)-O(1)#1	146.4(4)		

Symmetry transformations used to generate equivalent atoms:

#1 -x+1,-y+1,-z+1 #2 -x,-y+1,-z+1 #3 -x+1,-y,-z+1

The analysis of the geometry of each Gd(III), Sm(III), Ba(II) and Y(III) center for the six crystal structures has been carried out using the SHAPE software, version 2.1[4].

Gd(1) and Gd(3) display Gd-O bonds relative to two bidentate chelating propionate groups, two bridging propionate groups (to Gd(2) and Gd(4)), and two oxygen atoms shared between a propionate group which is chelating a neighboring Gd center. Similarly, Gd(2) and Gd(4) centers show coordination to eight propionate ligands with similar coordination as in the Gd(1) and Gd(3) centers, and additionally to the oxygen atom deriving from a HProp each (O11 and O27, respectively). The displayed geometries are distorted triangular dodecahedrons for Gd(1) and Gd(3), and muffin geometries for both Gd(2) and Gd(4) (S values reported in Table S6). These geometries result in a connectivity of polymeric 1D-chains, as displayed in Figure 1b. The geometries displayed by the Gd(III) centers in the crystal structure of $Gd(Prop)_3$ reported in the main text result in a connectivity of polymeric 1D-chains through a combination of μ2:η2:η1 (mixed bidentate chelating and bridging) and μ2:η1:η1 (bridging) coordination modes (as shown in Figure 1(b) in the main text). The mixture between bridging and bidentate chelating arrangement of the ligands is supported by the calculated value of Δν from FTIR-ATR reported in the synthetic details at the beginning of this section.

Table S6. Geometry distortion analysis for $Gd(Prop)_3$ using S-parameter[5] calculated with SHAPE[4].

Compound	*Geometry*[a]	*S-value*
Gd(Prop)₃ *Gd(1)*	HBPY-8	13.364
	CU-8	8.846
	SAPR-8	3.859
	**TDD-8**	**3.067**
	JGBF-8	12.797
	JETBPY-8	25.967
	JBTP-8	3.896
	BTPR-8	3.389
	JSD-8	5.822
	ETBPY-8	22.140

	TT-8	9.598
Gd(Prop)₃ *Gd(3)*	HBPY-8	13.367
	CU-8	9.903
	SAPR-8	4.602
	**TDD-8**	**2.966**
	JGBF-8	12.949
	JETBPY-8	25.628
	JBTP-8	4.182
	BTPR-8	3.640
	JSD-8	6.247
	ETBPY-8	21.996
	TT-8	9.828
Gd(Prop)$_3$ *Gd(2)*	EP-9	34.216
	HH-9	8.255
	**MFF-9**	**1.497**
Gd(Prop)$_3$ *Gd(4)*	EP-9	34.459
	HH-9	8.671
	**MFF-9**	**1.508**

Closer values for the geometry of each metal center are reported in bold. [a]HBPY-8 = hexagonal bipyramid; CU-8 = cube, SAPR-8 = square antiprism; TDD-8 = triangular dodecahedron; JGBF-8 = Johnson – Gyrobifastigium; JETBPY-8 = Johnson - Elongated triangular bipyramid; JBTP-8 = biaugmented trigonal prism; BTPR-8 = biaugmented trigonal prism; JSD-8 = Snub disphenoid; ETBPY-8 = elongated trigonal bipyramid; TT-8 = triakis tetrahedron, EP-9 = enneagon; HH-9 = hula-hoop; MFF-9 = muffin.

In the complex Ba(Prop)$_2$ crystal structure reported in the main text, the Ba(3) and Ba(6) centers are coordinated to oxygen atoms deriving from two H_2O molecules (O4 and O6 for Ba(3) and O1 and O3 for Ba(6)) and various combinations of coordination modes of the propionate ligands with the neighboring Ba(II) centers, comprising μ2:η2:η1, μ4:η3:η2, μ3:η3:η2, μ3:η2:η2, and μ2:η2:η2 (tritopic and ditopic oxygen atoms). Differently, Ba(1) and Ba(4) are showing no coordination to any H_2O molecule, and the Ba-O bonds are deriving solely from propionate ligands through a

mixture of μ2:η2:η1, μ3:η2:η2, and μ4:η3:η2 connectivity with other Ba(II) centers (specifically, with Ba(1), Ba(2), Ba(3), Ba(4) and Ba(7) for Ba(3) and Ba(1), Ba(4), Ba(5), Ba(6), and Ba(7) for Ba(6)). Ba(5) displays a Ba-O bond to the oxygen atom of a H_2O molecule (O5) and coordination to Ba(1), Ba(2), Ba(4), Ba(6), and Ba(7) through combinations of μ4:η3:η2 and μ3:η2:η2 modes of the propionate groups. Ba(2) (coordination number: 10) follows an arrangement of the propionate ligands through μ2:η2:η1, μ2:η2:η2, μ3:η2:η2, and μ4:η3:η2 additionally to a bond to a H_2O molecule (O7). Ba(7) bears a distorted hexagonal bipyramid geometry from coordination number 8 and displays Ba-O bonds deriving from two H_2O molecules (O2 and O5) together with a combination of various coordination modes of the ligands ($\mu_2:\eta^2:\eta^1$, $\mu_2:\eta^2:\eta^2$, and $\mu_4:\eta^3:\eta^2$) with the neighboring Ba(1), Ba(2), Ba(3), Ba(4), Ba(5), and Ba(6). Ba(1), Ba(3), Ba(4), Ba(5), and Ba(6) are the centers with coordination number 9 and accommodate two types of geometries: Ba(1), Ba(4), and Ba(5) show a distorted hula-hoop geometry, whereas Ba(3) and Ba(6) exhibit a muffin geometry. Ba(2) shows, conversely, coordination number 10 and a distorted tetradecahedron geometry, whereas Ba(7) bears a distorted hexagonal bipyramid geometry from coordination number 8. S values for each Ba(II) center are reported in Table S7.

Table S7. Geometry distortion analysis for $Ba(Prop)_2$ using S-parameter calculated with SHAPE.

Compound	*Geometry*[a]	*S-value*
Ba(Prop)₂ *Ba(1)*	EP-9	21.609
	**HH-9**	**6.884**
	MFF-9	8.229
Ba(Prop)₂ *Ba(3)*	EP-9	35.273
	HH-9	10.485
	**MFF-9**	**2.404**
Ba(Prop)₂ *Ba(4)*	EP-9	22.666
	**HH-9**	**4.264**
	MFF-9	9.636
Ba(Prop)₂ *Ba(5)*	EP-9	23.872
	**HH-9**	**7.274**
	MFF-9	8.584

Ba(Prop)₂ *Ba(6)*	EP-9	36.979
	HH-9	9.55
	**MFF-9**	**2.120**
Ba(Prop)₂ *Ba(2)*	HD-10	10.795
	JATDI-10	16.233
	JBCSAPR-10	7.693
	JMBIC-10	7.595
	JSPC-10	6.042
	SDD-10	5.000
	**TD-10**	**4.334**
Ba(Prop)₂ *Ba(7)*	**HBPY-8**	**6.819**
	CU-8	7.764
	SAPR-8	17.301
	TDD-8	14.617
	JGBF-8	13.258
	JETBPY-8	19.496
	JBTP-8	15.575
	BTPR-8	15.295
	JSD-8	14.326
	ETBPY-8	16.862
	TT-8	8.575

Closer values for the geometry of each metal centre are reported in bold. [a]HBPY-8 = Hexagonal bipyramid; CU-8 = cube, SAPR-8 = square antiprism; TDD-8 = triangular dodecahedron; JGBF-8 = Johnson – Gyrobifastigium; JETBPY-8 = Johnson - elongated triangular bipyramid; JBTP-8 = biaugmented trigonal prism; BTPR-8 = biaugmented trigonal prism; JSD-8 = snub disphenoid; ETBPY-8 = elongated trigonal bipyramid; TT-8 = triakis tetrahedron, EP-9 = enneagon; HH-9 = Hula-hoop; MFF-9 = muffin; HD-10 = hexadecahedron; JADTI-10 = augmented tridiminished icosahedron; JBCSAPR-10 = bicapped square antiprism; JMBIC-10 = metabidiminished icosahedron; JSPC = sphenocorona; SDD-10 = staggered dodecahedron; TD-10 = tetradecahedron.

Table S8. Geometry distortion analysis for $Y(Prop)_3$ using S-parameter calculated with SHAPE.

Compound	*Geometry*[a]	*S-value*
Y(Prop)₃ *Y(1)*	EP-9	34.539
	HH-9	9.831
	**MFF-9**	**1.973**

Closer values for the geometry of each metal centre are reported in bold. [a]EP-9 = enneagon, HH-9 = hula-hoop, MFF-9 = muffin.

For each yttrium centre in the $Y(Prop)_3$ crystal structure, two propionate ligands are exhibiting a μ_1:η^1: η^1 (bidentate chelating) coordination mode, and two present a mixed μ_2:η^2: η^1 (bidentate chelate and bridged) coordination mode. The combination of coordination modes accords with the value of $\Delta\nu$ reported in the synthetic details reported at the beginning of this section, characteristic to a mixed bridging and bidentate chelating coordination of the ligands.

The propionate ligands in $Sm(Prop)_3$ display two types of coordination modes, mixing μ_2:η^2: η^1 and μ_2:η^1: η^1. The first is a mixture of bidentate chelating and bridging between Sm(1)-Sm(1) and Sm(2)-Sm(2) centers, which are responsible for the formation of 1D chains. The second derives from the bridging propionate groups which arranges the Sm(III) centers into distorted square lattice 2D-sheets along the *ab* plane, as shown in the main text. This finding is in good agreement with the value of $\Delta\nu$ reported in the synthetic details reported at the beginning of this section, corresponding to a mixed bridging and bidentate chelating coordination of the ligands. Additionally, the difference among coordination modes is displayed in Figure S7, clearly evidencing that the bridging coordination along the $[\bar{1}10]$ direction is occurring alternatively between Sm(1) and Sm(2) centers.

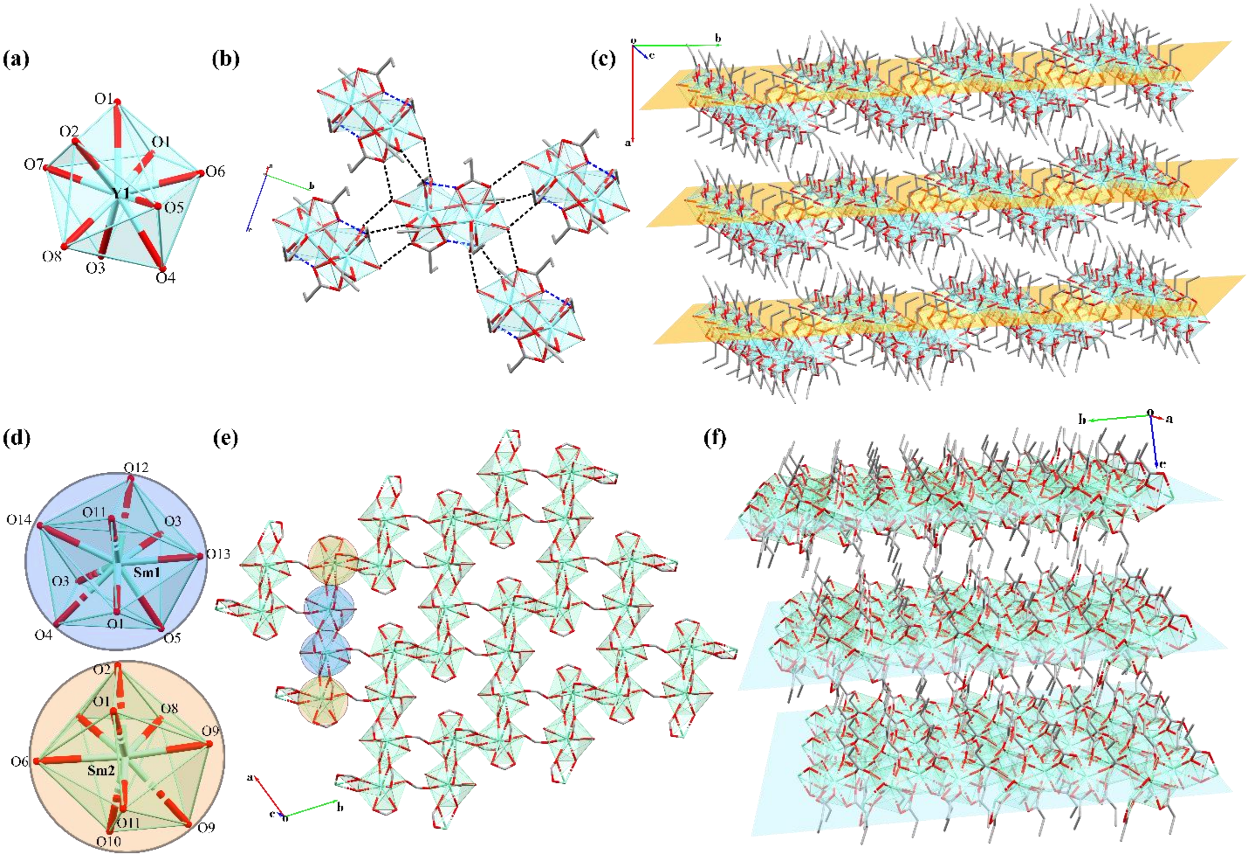

Figure S6. Representation of the (a) Y(III) core in the dimeric Y(Prop)$_3$ unit. (b) Intramolecular (blue dashed lines) and intermolecular H-bonds (black dashed lines) responsible for the association between dimers and formation of the zig-zag 2D-sheets of Y(Prop)$_3$ shown in (c). Representation of the (d) two different Sm(III) cores which are constituting the distorted square lattice 2D-sheets (e) and packing of the 2D-sheets of Sm(Prop)$_3$ in (f).

Table S9. Geometry distortion analysis for Sm(Prop)$_3$ using S-parameter calculated with SHAPE.

Compound	*Geometry*[a]	*S-value*
Sm(Prop)$_3$ *Sm(1)*	EP-9	31.488
	HH-9	7.099
	**MFF-9**	**2.772**
Sm(Prop)$_3$ *Sm(2)*	EP-9	31.554
	HH-9	10.261
	**MFF-9**	**3.388**

Closer values for the geometry of each metal centre are reported in bold. [a]EP-9 = enneagon, HH-9 = hula-hoop, MFF-9 = muffin.

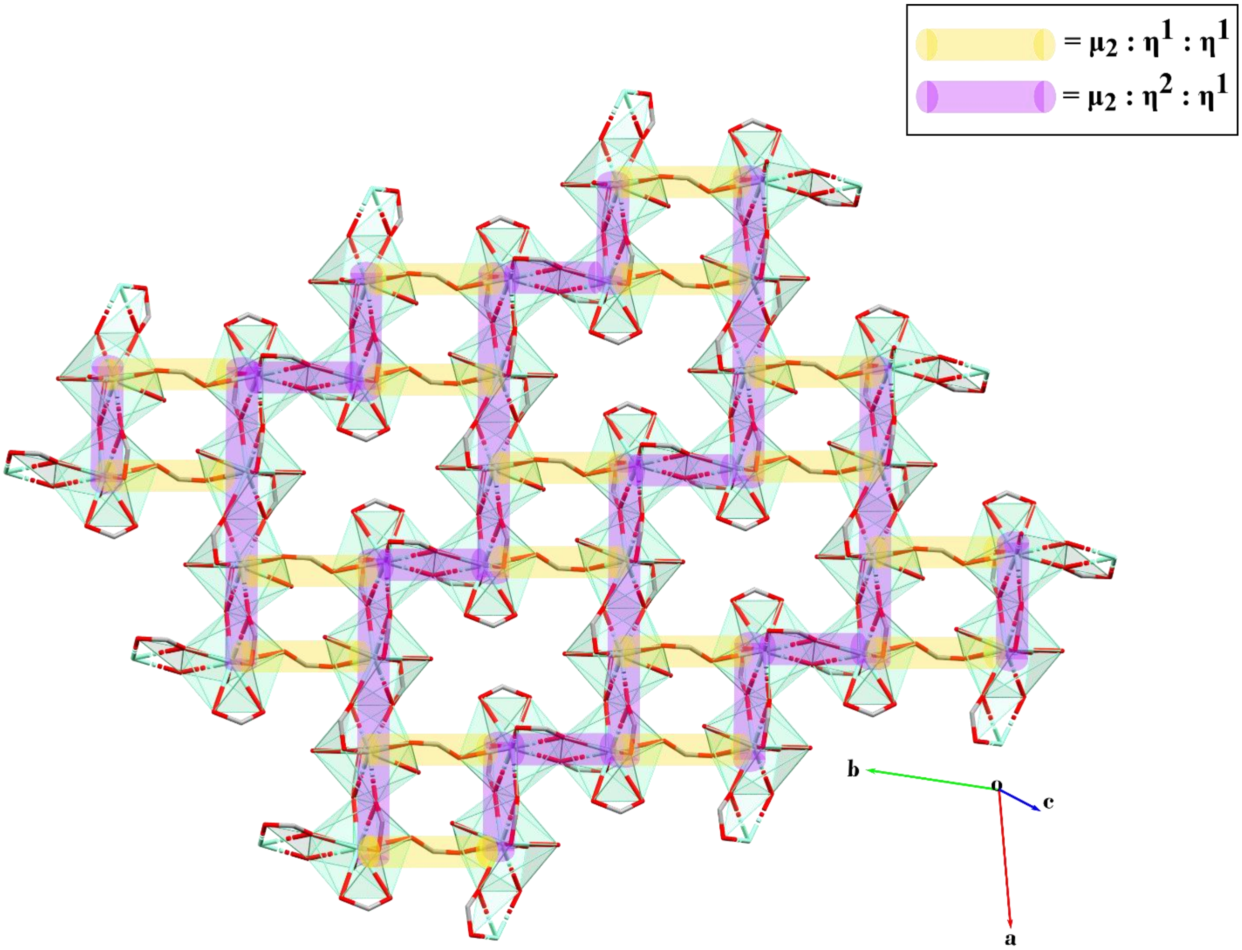

Figure S7. Different coordination modes of the propionate ligands forming the distorted square lattice 2D-sheets in the structure of $Sm(Prop)_3$.

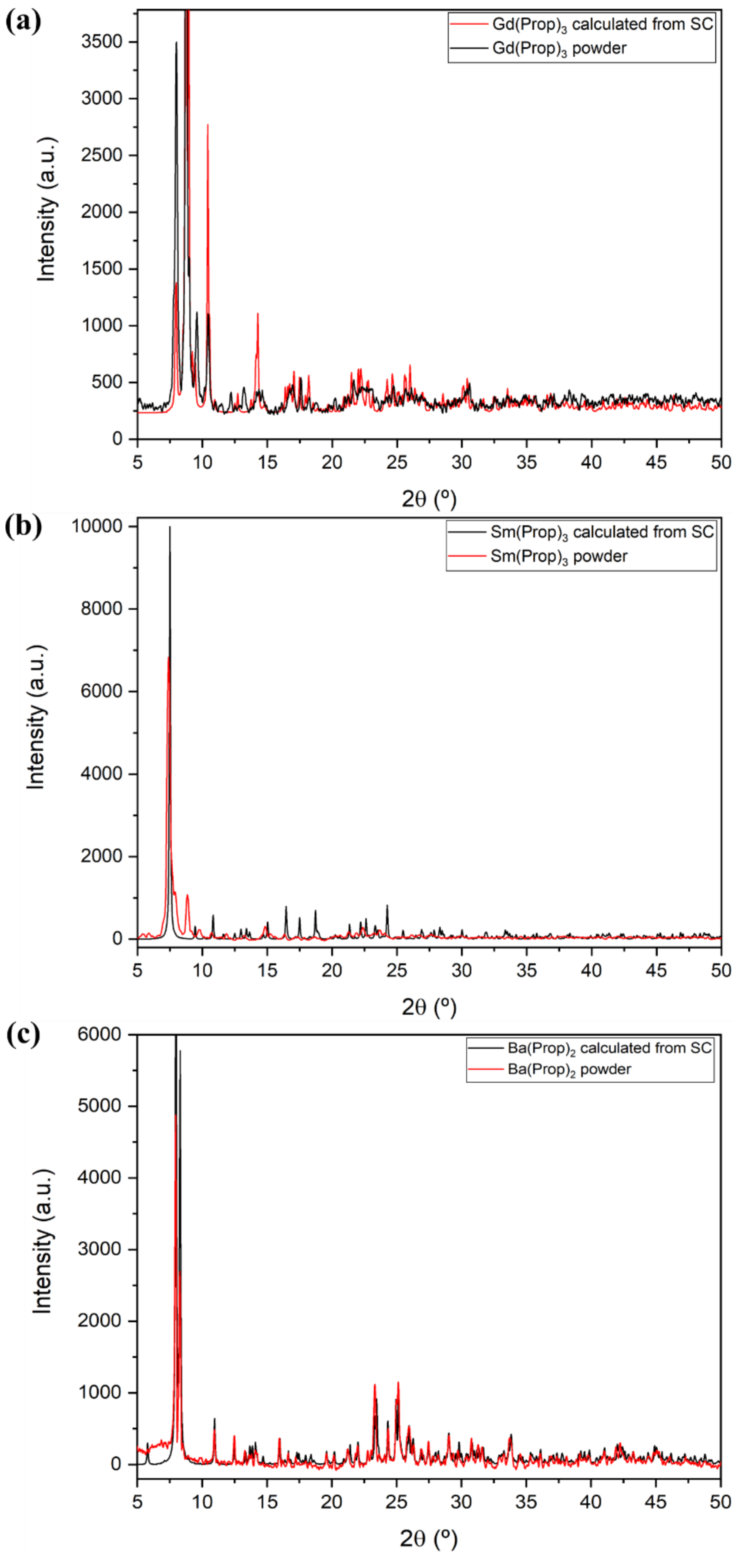

Figure S8. Comparison of the measured PXRD and the pattern calculated from the single crystal (SC) structure for (a) $Gd(Prop)_3$, (b) $Sm(Prop)_3$, and (c) $Ba(Prop)_2$.

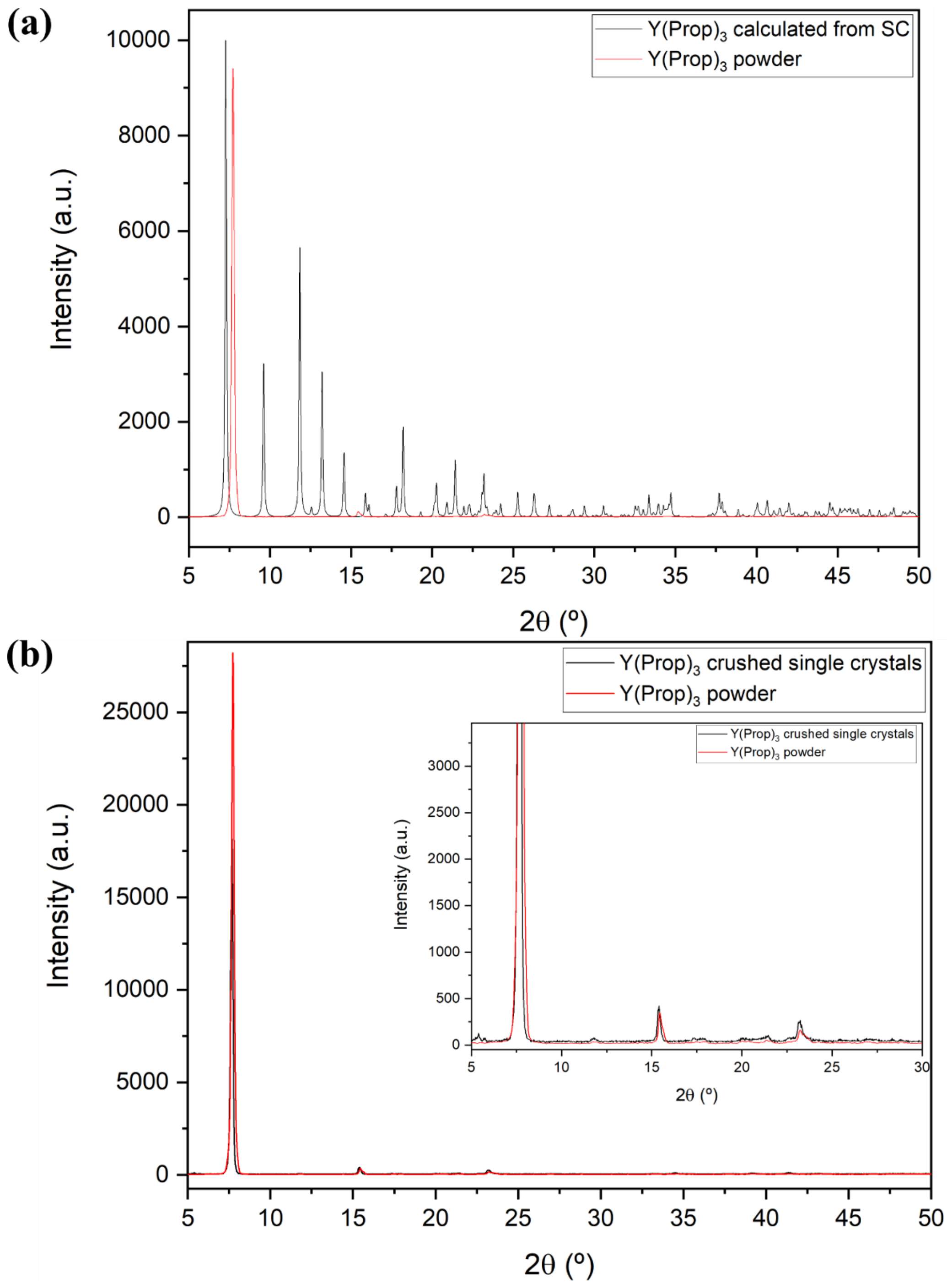

Figure S9. (a) Unmatching PXRD and the pattern calculated from the single crystal (SC) structure for $Y(Prop)_3$. (b) XRD comparison between a sample of the crushed SC from (a) and the synthesized $Y(Prop)_3$ powder. The matching between the two is suggesting the possibility of the existence of various types of SC in the same crystallization vessel, with a preferential orientation exhibited by the measured SC.

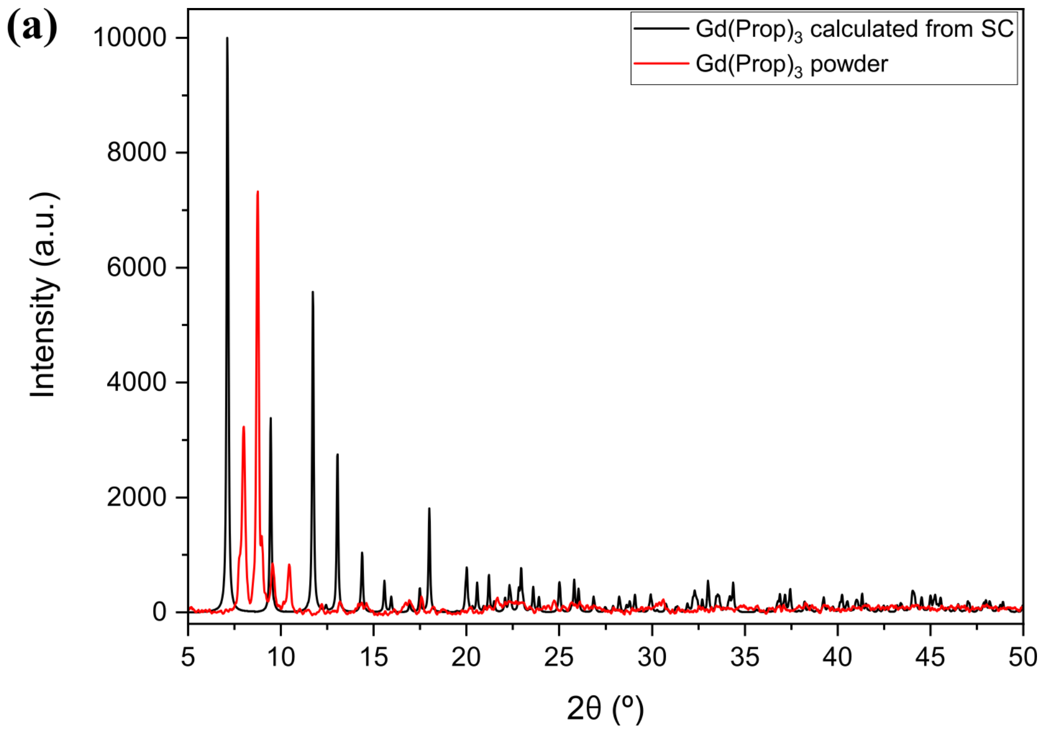

Figure S10. Comparison between PXRD and the patterns calculated from the single crystal (SC) structure for (a) $Gd(Prop)_3$ obtained through recrystallization in MeOH, exhibiting an unmatching pattern.

Gadolinium propionate results in a different, novel crystal structure when crystallization is carried out through slow evaporation in MeOH. It crystallizes in a monoclinic P21/c space group and displays dimeric units formed by two identical Gd(III) centers bearing $[GdO_9]$ cores with coordination number 9 and a distorted muffin geometry (S values reported in Table S9). For each gadolinium centre, two propionate ligands are exhibiting a $\mu_1:\eta^1:\eta^1$ (bidentate chelating) coordination mode, and two present a mixed $\mu_2:\eta^2:\eta^1$ (bidentate chelate and bridged) coordination mode. The two H_2O molecules in each Gd(III) center (O7 and O8) assemble the dimeric units in polymeric 1D-chains through inter- and intramolecular H-bonds. The combination of coordination modes is in accordance with the value of $\Delta\nu$ reported in Experimental Section, characteristic of a mixed bridging and bidentate chelating coordination of the ligands.

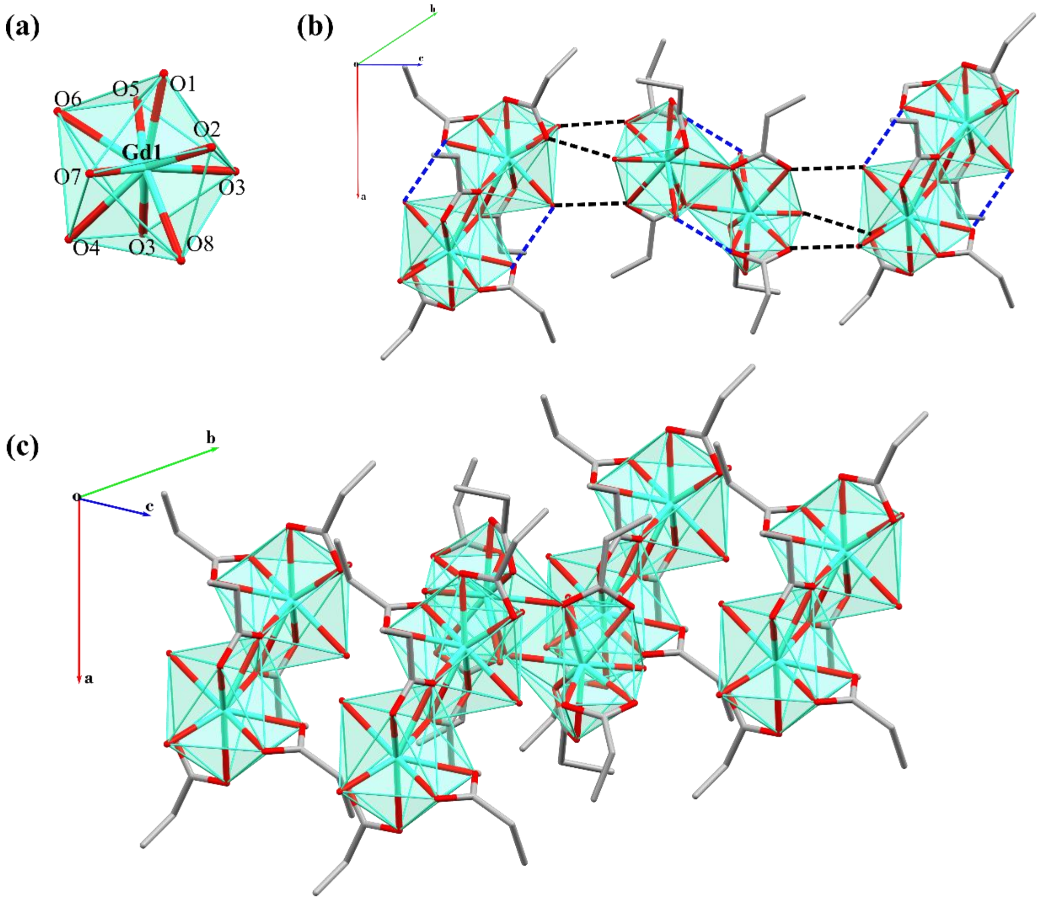

Figure S11. Representation of the (a) Gd (III) core in the dimeric Gd(Prop)$_3$ unit. (b) Intramolecular (blue dashed lines) and intermolecular H-bonds (black dashed lines) responsible for the formation of an array of polymeric 1D-chains (c).

Table S10. Crystal data and structure refinement for [Gd(Prop)$_3$(OH$_2$)$_2$]$_n$.

	**[Gd(Prop)$_3$(OH$_2$)$_2$]$_n$**
Empirical formula	$C_9H_{19}GdO_8$
Formula weight	412.49
Temperature (K)	294(2)
Wavelength (Å)	0.71073
Crystal system	Monoclinic
Space group	P 21/c
Unit cell dimensions	
a (Å)	12.498(5)
b (Å)	14.228(6)
c (Å)	8.966(4)
α (deg)	90
β (deg)	97.378(7)

γ (deg)	90
V (Å^3)	1581.1(11)
Z	4
D_{calc} (Mg/m^3)	1.733
Absorption coefficient (mm^{-1})	4.219
F(000)	804
Crystal size (mm^3)	0.16x0.15x0.07
θ range for data collection (deg)	1.643 to 28.444
hkl ranges	-12<=h<=11<br>0<=k<=19<br>0<=l<=16
Reflections collected	36190
Independent reflections	3963
Completeness to θ (%)	100.0
Absorption correction	Semi-empirical from equivalents
Max. and min. transmission	1 and 0.592
Refinement method	Full-matrix least-squares on $\|F\|^2$
Data / restraints/ parameters	3963 / 29 / 166
Goodness-of-fit on $\|F\|^2$	1.086
Final R indices [I>2sigma(I)]	R_1 = 0.0815<br>wR_2 = 0.2356
R indices (all data)	R_1 = 0.1196<br>wR_2 = 0.2521
Largest diff. peak and hole (e·Å^{-3})	7.060 and -3.035

Table S11. Bond lengths and torsion angles of $Gd(Prop)_3$ obtained by recrystallization in MeOH.

**Bond lengths (Å)**			
Gd(1)			
Gd(1)-O(7)	2.293(11)	Gd(1)-O(2)	2.476(9)
Gd(1)-O(3)	2.398(8)	Gd(1)-O(6)	2.493(10)
Gd(1)-O(8)	2.414(9)	Gd(1)-O(4)#1	2.522(10)
Gd(1)-O(1)	2.434(13)	Gd(1)-O(3)#1	2.556(9)
Gd(1)-O(5)	2.441(9)		

**Bond angles (deg)**
Gd(1)

O(7)-Gd(1)-O(3)	153.3(3)	O(1)-Gd(1)-O(6)	78.6(4)
O(7)-Gd(1)-O(8)	83.7(3)	O(5)-Gd(1)-O(6)	52.8(3)
O(3)-Gd(1)-O(8)	79.3(3)	O(2)-Gd(1)-O(6)	128.2(4)
O(7)-Gd(1)-O(1)	78.9(5)	O(7)-Gd(1)-O(4)#1	78.6(4)
O(3)-Gd(1)-O(1)	95.3(4)	O(3)-Gd(1)-O(4)#1	115.9(3)
O(8)-Gd(1)-O(1)	127.4(4)	O(8)-Gd(1)-O(4)#1	74.6(4)
O(7)-Gd(1)-O(5)	129.6(3)	O(1)-Gd(1)-O(4)#1	145.9(4)
O(3)-Gd(1)-O(5)	72.8(3)	O(5)-Gd(1)-O(4)#1	98.3(4)
O(8)-Gd(1)-O(5)	144.7(3)	O(2)-Gd(1)-O(4)#1	140.4(4)
O(1)-Gd(1)-O(5)	77.3(4)	O(6)-Gd(1)-O(4)#1	72.4(4)
O(7)-Gd(1)-O(2)	74.1(4)	O(7)-Gd(1)-O(3)#1	127.8(4)
O(3)-Gd(1)-O(2)	81.5(3)	O(3)-Gd(1)-O(3)#1	66.1(3)
O(8)-Gd(1)-O(2)	74.3(4)	O(8)-Gd(1)-O(3)#1	73.1(3)
O(1)-Gd(1)-O(2)	53.3(4)	O(1)-Gd(1)-O(3)#1	150.9(4)
O(5)-Gd(1)-O(2)	121.3(4)	O(5)-Gd(1)-O(3)#1	75.9(3)
O(7)-Gd(1)-O(6)	79.3(3)	O(2)-Gd(1)-O(3)#1	137.4(3)
O(3)-Gd(1)-O(6)	125.4(3)	O(6)-Gd(1)-O(3)#1	93.9(3)
O(8)-Gd(1)-O(6)	145.2(4)	O(4)#1-Gd(1)-O(3)#1	50.6(3)

Symmetry transformations used to generate equivalent atoms:
#1 -x+1,-y+1,-z+1

Table S12. Geometry distortion analysis for $Gd(Prop)_3$ obtained by recrystallization in MeOH using S-parameter calculated with SHAPE.

Compound	*Geometry*[a]	*S-value*
Gd(Prop)₃ *Gd(1)*	EP-9	33.989
	HH-9	10.049
	**MFF-9**	**2.007**

Closer values for the geometry of each metal centre are reported in bold. [a]EP-9 = enneagon, HH-9 = hula-hoop, MFF-9 = muffin.

Section II. Metal propionates powders decomposition

All powder products show a first, low temperature mass loss corresponding to dehydration and/or residual solvent elimination, with the only exception being the anhydrous Cu(Prop)$_2$.

From previous studies on the decomposition of calcium propionate[6], and the thermal analysis of various lanthanide propionates by Grivel et al.[7], the decomposition mechanism in an inert atmosphere commonly proposed for salts deriving from carboxylic acids generates mainly symmetrical ketones (with chain lengths varying according to the substituent groups present) and CO_2, following a radical pathway in which the recombination of the radical species gives rise to the formation of the aforementioned symmetrical ketones. However, for metals that can easily undergo redox reactions, the main volatile is the corresponding salt acid[8,9].

The propionates of Y and other RE (Sm, Gd, and Yb) follow a similar mechanism: the radical pathway is described in Scheme S1(a). Generally, during the first mass loss at high temperatures, corresponding to 376 ºC, 356 ºC, and 371 ºC, for Y(Prop)$_3$, Gd(Prop)$_3$, and Yb(Prop)$_3$, respectively, there is the release of the symmetrical ketone 3-pentanone, CO_2, and the oxycarbonate species of the respective rare earth element. The latter species are stable up to higher temperatures and are not present in the decomposition profile of the rest of the metal propionates precursors.

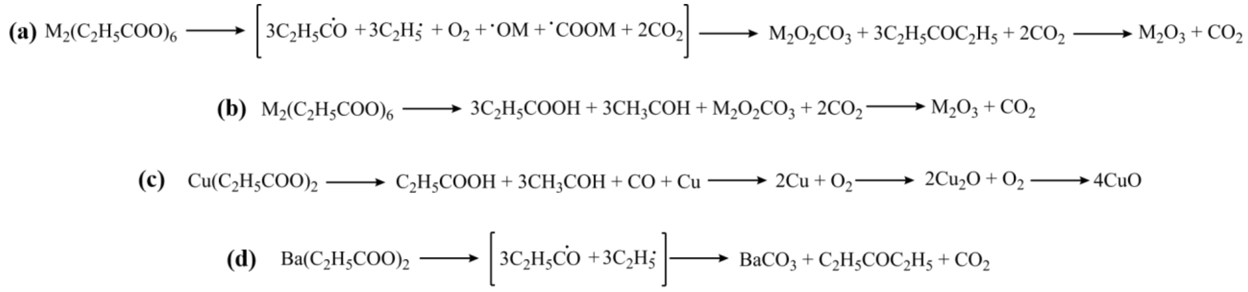

Scheme S1. Decomposition mechanisms for metal propionates: **(a)** radical path and **(b)** oxidation mechanism for Y(Prop)$_3$ and other RE metal propionates (M = Y, Sm, Gd, or Yb); **(c)** reductive mechanism for Cu(Prop)$_2$; **(d)** radical path for the case of Ba(Prop)$_2$

Ultimately, these decompose to form the final rare earth sesquioxide by releasing CO_2 at the temperatures of 552 ºC, 426 ºC, and 439 ºC, for Y(Prop)$_3$, Gd(Prop)$_3$, and Yb(Prop)$_3$, respectively (see Figures S12-S14). The formation of Gd_2O_3, Yb_2O_3, and Y_2O_3 is confirmed by the masses of

the final residues which have similar values if compared to the theoretical ones for complete conversions of the propionates to sesquioxides: 37.8% found for Y(Prop)$_3$ (expected 36.6%), 49.6% found for Gd(Prop)$_3$ (expected 47.8%), and 50.9% found for Yb(Prop)$_3$ (expected 50.2%). Moreover, XRD data of the final residue showing the formation of the sesquioxide can be found in Fig. S15(a-c).

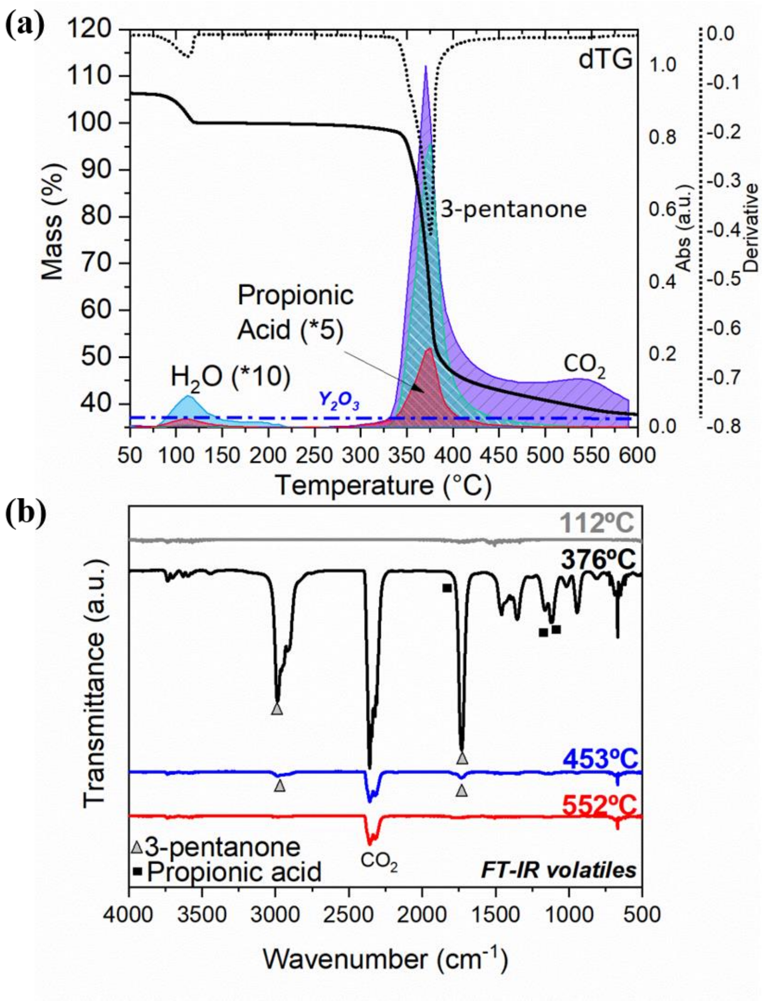

Figure S12. (a) TGA decomposition curve (black) of Y(Prop)$_3$ powder in inert conditions (N_2). The dotted line shows the TG derivative, whereas the gas evolution is shown as individual curves according to their thermal stability and elimination. The dashed and dotted blue line indicates the expected value for total decomposition to Y_2O_3. **(b)** FT-IR spectra corresponding to the main decomposition steps of Y(Prop)$_3$ powder from TGA in **(a)**.

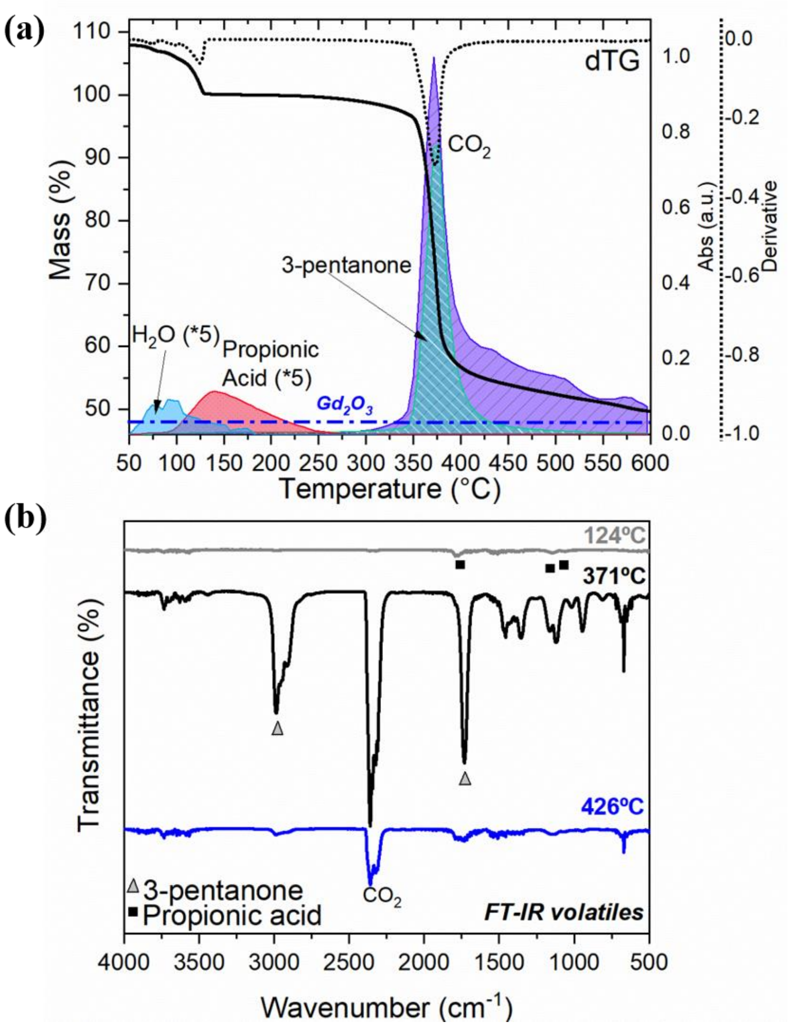

Figure S13. (a) TGA decomposition curve (black) of Gd(Prop)$_3$ powder in inert conditions (N_2). The dotted line shows the TG derivative, whereas the gas evolution is shown as individual curves according to their thermal stability and elimination. The dashed and dotted blue line indicates the expected value for total decomposition to Gd_2O_3. **(b)** FT-IR spectra corresponding to the main decomposition steps of Gd(Prop)$_3$ powder from TGA in **(a)**.

(a)

(b)

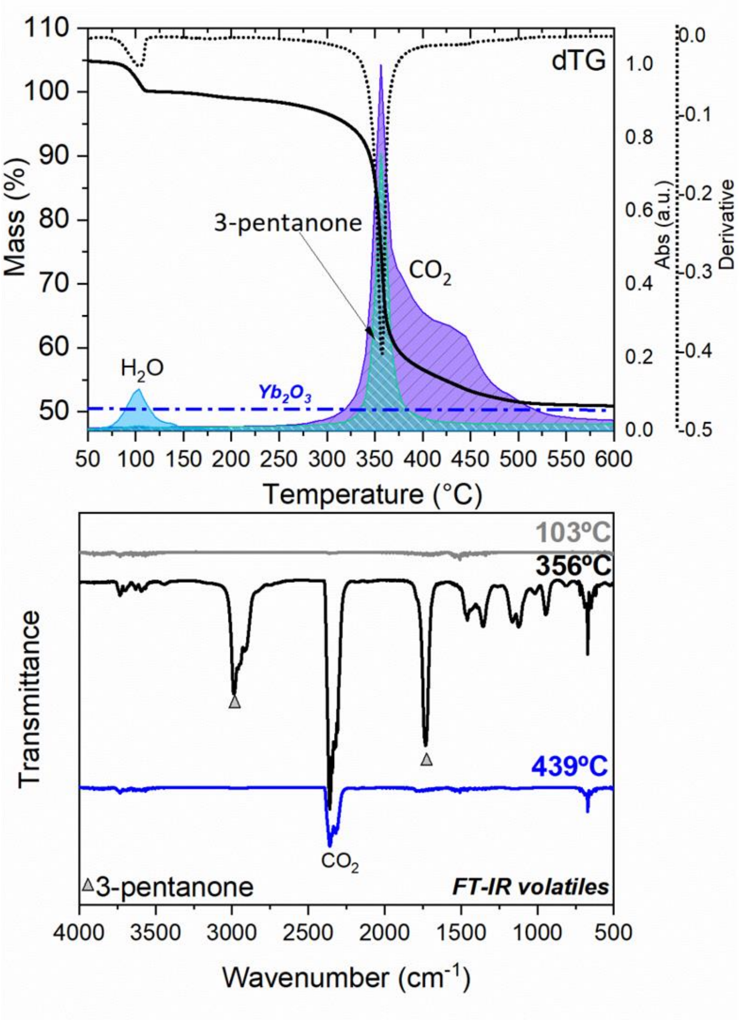

Figure S14. (a) TGA decomposition curve (black) of Yb(Prop)$_3$ powder in inert conditions (N_2). The dotted line shows the TG derivative, whereas the gas evolution is shown as individual curves according to their thermal stability and elimination. The dashed and dotted blue line indicates the expected value for total decomposition to Yb_2O_3. **(b)** FT-IR spectra corresponding to the main decomposition steps of Yb(Prop)$_3$ powder from TGA in **(a)**.

Nonetheless, Sm(Prop)$_3$ shows a mixed decomposition pathway (Figure S16), in which the products of the radical path are present together with the additional evolution of acetaldehyde and propionic acid. This suggests that it is also following an oxidative mechanism (Scheme S1b), in which the degradative oxidation of the propionate groups to acetaldehyde, CO_2 and propionic acid is initiated by the presence of O_2. Both mechanisms finally lead to the formation of the oxycarbonate species, which upon heating continues its decomposition to the sesquioxide, in this case Sm_2O_3, as suggested by the final residue mass percentage (49.7%, as compared to the

expected 47.2% theoretical value), and confirmed by XRD analysis (Fig. S15d). As this analysis was carried out under an N_2 gas flow, a possible explanation for this behavior could be the presence of a residual O_2 content in the TGA furnace, which may trigger the second mechanism to be followed.

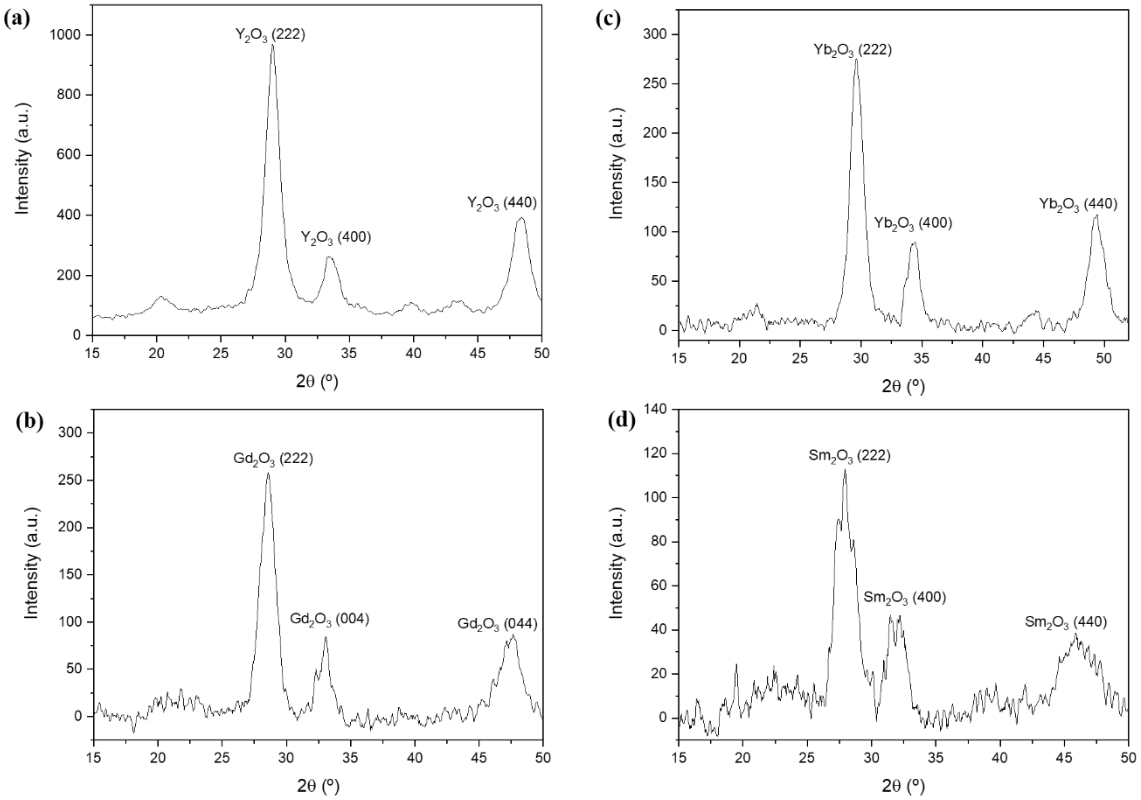

Figure S15. XRD of the final residue after the TG experiment for (a) $Y(Prop)_3$ showing the formation of Y_2O_3; (b) $Gd(Prop)_3$, showing the formation of Gd_2O_3; (c) $Yb(Prop)_3$, showing the formation of Yb_2O_3; (d) $Sm(Prop)_3$, showing the formation of Sm_2O_3.

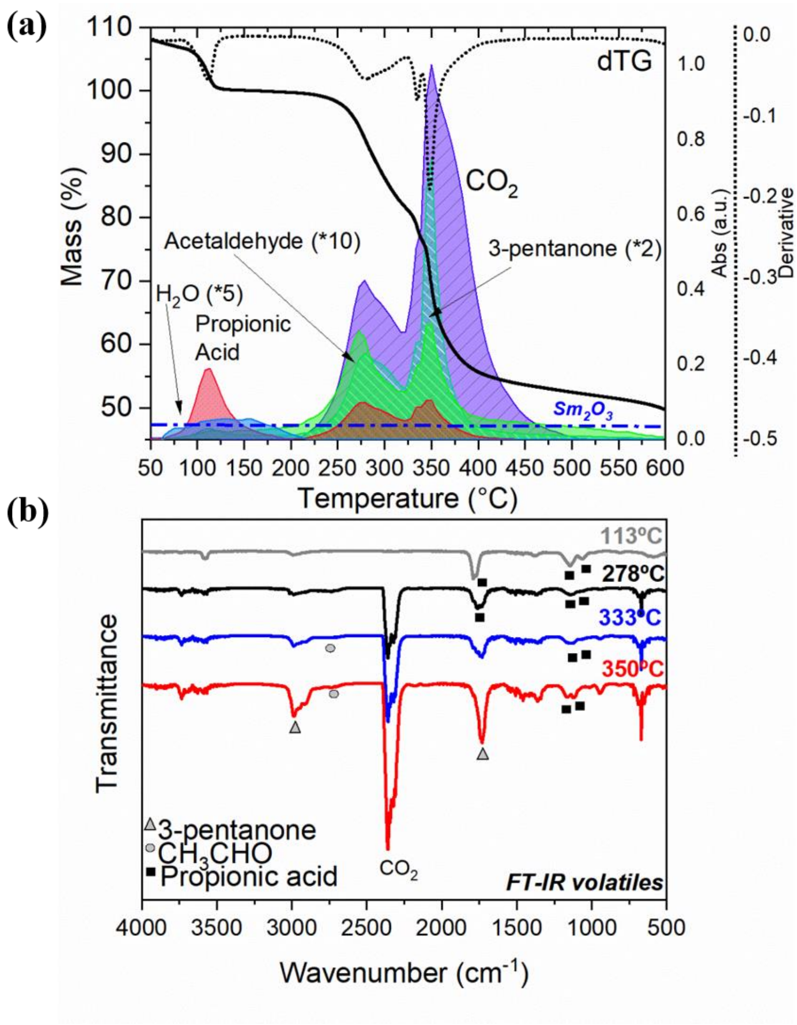

Figure S16. (a) TGA decomposition curve (black) of Sm(Prop)$_3$ powder in inert conditions (N_2). The dotted line shows the TG derivative, whereas the gas evolution is shown as individual curves according to their thermal stability and elimination. The dashed and dotted blue line indicates the expected value for total decomposition to Sm_2O_3. (b) FT-IR spectra corresponding to the main decomposition steps of Sm(Prop)$_3$ powder from TGA in (a).

Note the similarities and differences of the DSC curves, displayed in Figure S17a-d, of the couples of compounds Y(Prop)$_3$ and Yb(Prop)$_3$, Gd(Prop)$_3$ and Sm(Prop)$_3$: the latter both show an endothermic peak below 200 ºC, after the dehydration step. This endothermic process corresponds to the precursor melting, no correspondent peak in the dTG curve is present. Contrarily, there is no evidence of such a phenomenon in the DSC curves of Y(Prop)$_3$ and Yb(Prop)$_3$, in which the elimination of H_2O is the only mass loss before the propionate decomposition step.

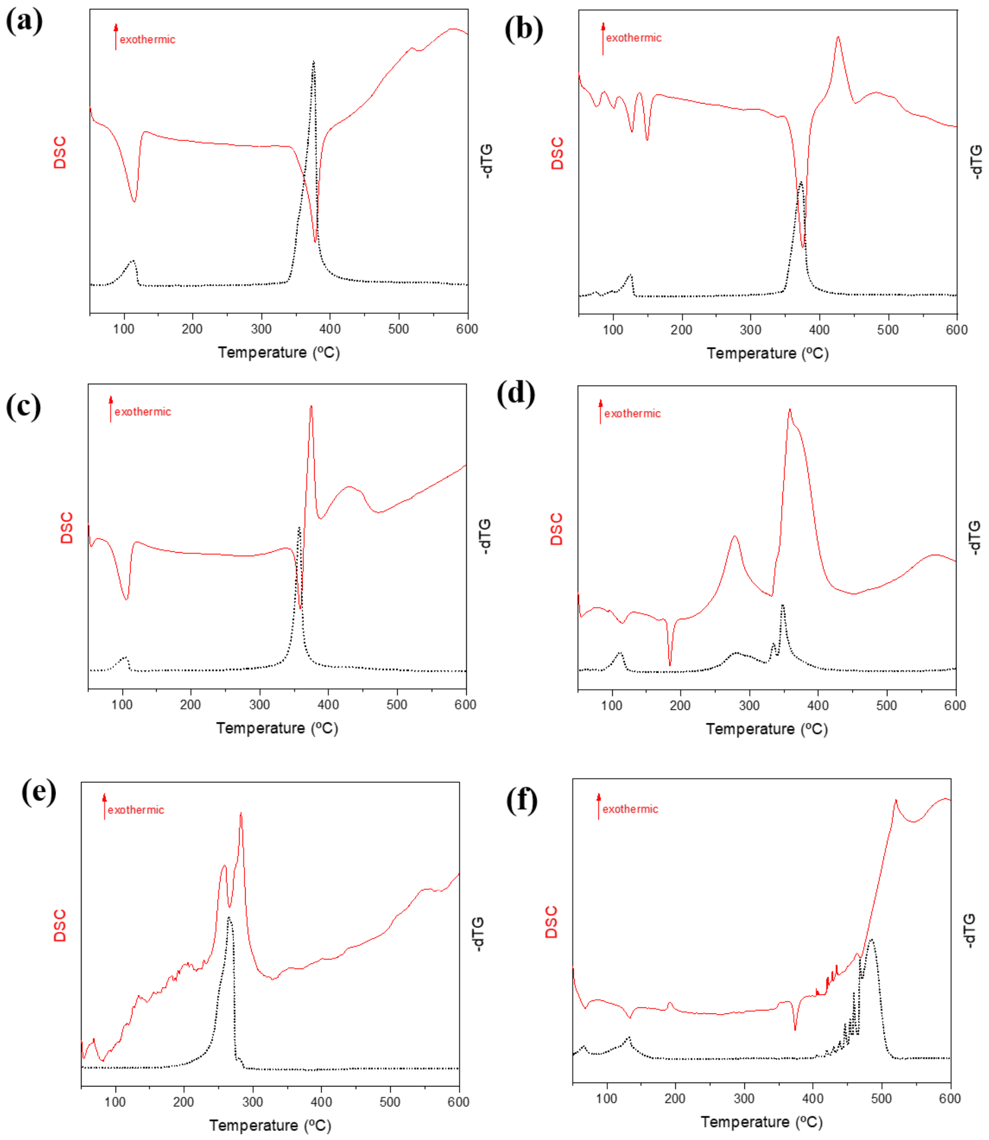

Figure S17. DSC and dTG curves for (a) $Y(Prop)_3$, (b) $Gd(Prop)_3$, (c) $Yb(Prop)_3$, (d) $Sm(Prop)_3$, (e) $Cu(Prop)_2$, (f) $Ba(Prop)_2$.

The decomposition of $Cu(Prop)_2$ differs from the abovementioned pathways (Figure S18): it is, instead, decomposing through a reductive mechanism Scheme S1c) to yield acetaldehyde, CO, CO_2, 3-pentanone, and propionic acid in a single step, highly exothermic reaction. The DSC curve in Figure S17e confirms the exothermic nature of the process in correspondence with the main decomposition step, at temperatures ranging between 235 ºC and 280 ºC. Thus, the full reduction to Cu (0) is promoted; however, the XRD of the final residue (Figure S19a) also shows partial oxidation to Cu_2O and CuO, corresponding to the slight mass increase at temperatures above 400

ºC, possibly due to presence of residual O_2. The discrepancy between the value of the mass of the final residue (34.8%) and the theoretical ones for Cu, Cu_2O, and CuO (30.3%, 37.9%, and 34.1%, respectively) is due to the formation of a mixture of the three compounds in unknown proportion.

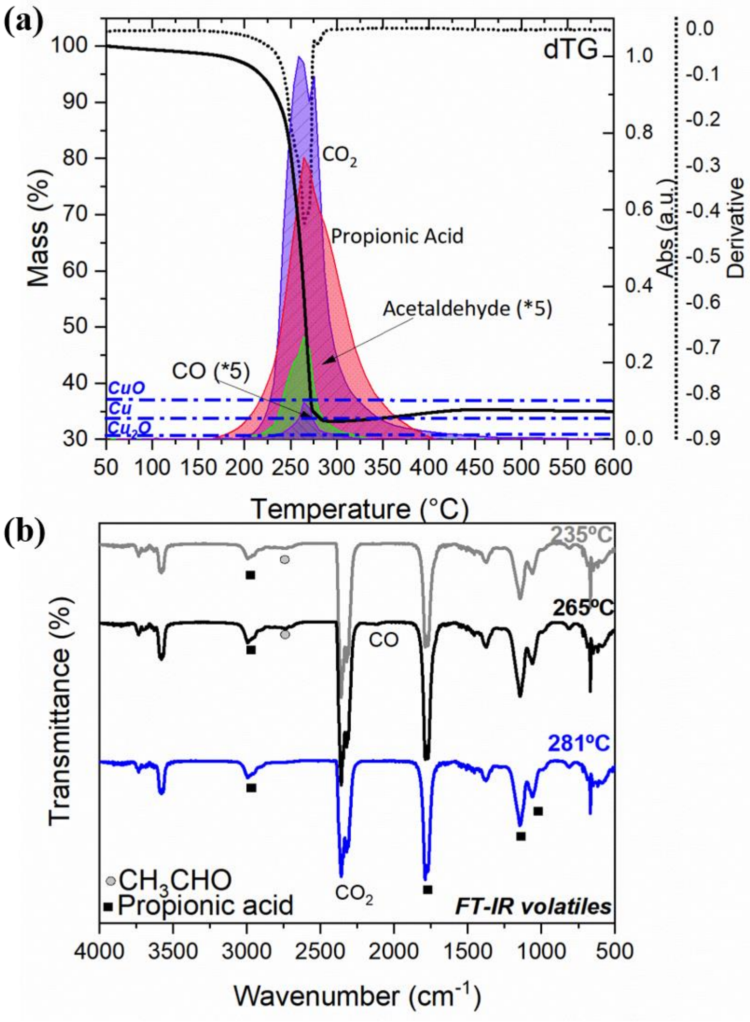

Figure S18. (a) TGA decomposition curve (black) of $Cu(Prop)_2$ powder in inert conditions (N_2). The dotted line shows the TG derivative, whereas the gas evolution is shown as individual curves according to their thermal stability and elimination. The dashed and dotted blue lines indicate the expected value for total decomposition to the oxides, CuO and Cu_2O, and metallic copper. (b) FT-IR spectra corresponding to the main decomposition steps of $Cu(Prop)_2$ powder from TGA in (a).

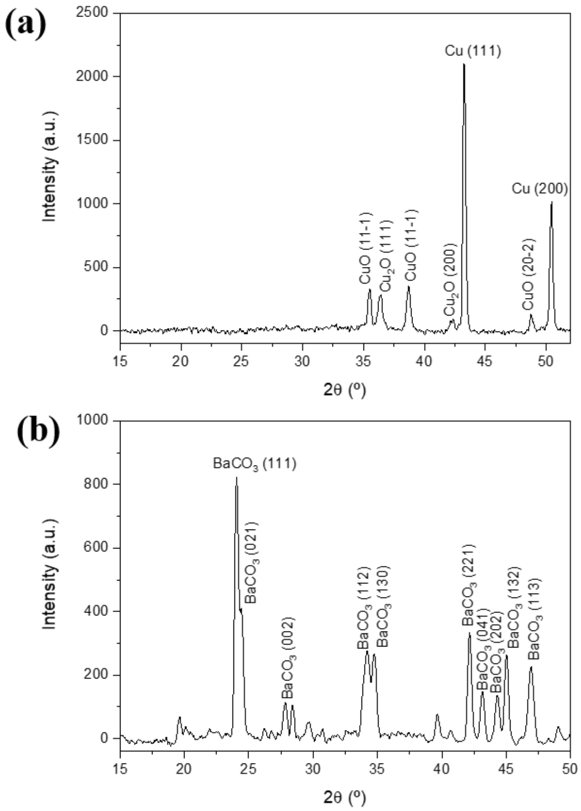

Figure S19. XRD of the final residue after the TG experiment for (a) Cu(Prop)$_2$, showing the formation of a mixture of Cu, Cu_2O, and CuO; (b) Ba(Prop)$_2$, showing the formation of $BaCO_3$ orthorhombic.

Ba(Prop)$_2$ decomposition in an inert atmosphere follows the radical path (Scheme S1(d)), however with some differences from the previous cases described above. The DSC curve in Figure S17f shows that following the initial dehydration and residual solvent elimination there is an exothermic peak corresponding to a crystallization of the product (192 ºC). Ba(Prop)$_2$ powder has a higher thermal stability compared to other precursors, showing the main decomposition step at 485 ºC. However, at 373 ºC, before decomposing, an endothermic peak is visible in the DSC curve with no corresponding peak in the dTG curve, related to the melting of the powder. This is also confirmed by the series of numerous sharp peaks due to the evolution of 3-pentanone gas bubbles through the melt, ending in the final decomposition.

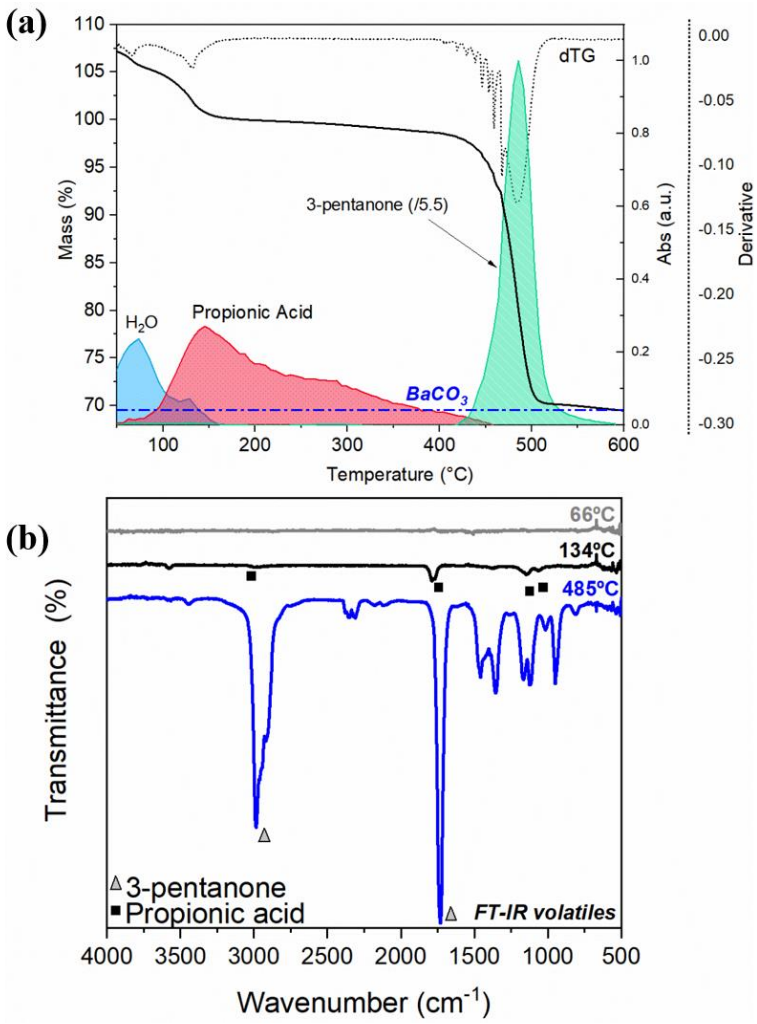

Figure S20. (a) TGA decomposition curve (black) of Ba(Prop)$_2$ powder in inert conditions (N_2). The dotted line shows the TG derivative, whereas the gas evolution is shown as individual curves according to their thermal stability and elimination. The dashed and dotted blue line indicates the expected value for total decomposition to the $BaCO_3$. **(b)** FT-IR spectra corresponding to the main decomposition steps of Ba(Prop)$_2$ powder from TGA in **(a)**.

As said, this precursor decomposes through the radical path, evolving 3-pentanone from the recombination of the intermediate radical species, to finally yield $BaCO_3$ with no CO_2 evolution due to reaction stoichiometry, in contrast to the previous metal propionates which followed this pathway (Figure S20). As oxides of barium at such low temperatures are not thermodynamically stable, the carbonate is the expected phase, as confirmed by the XRD of the final residue (Figure S19b) and its mass (69.5%, which is close to the expected theoretical value of 69.6% for $BaCO_3$ formation from Ba(Prop)$_2$).

Therefore, all of the synthesized metal propionates exhibit high purity and display a thermal behavior following established decomposition pathways to yield the expected oxides or carbonates, marking a promising starting point for their application in the CSD of REBCO precursor solutions for the preparation of superconducting films.

Section III. Metal propionates films decomposition

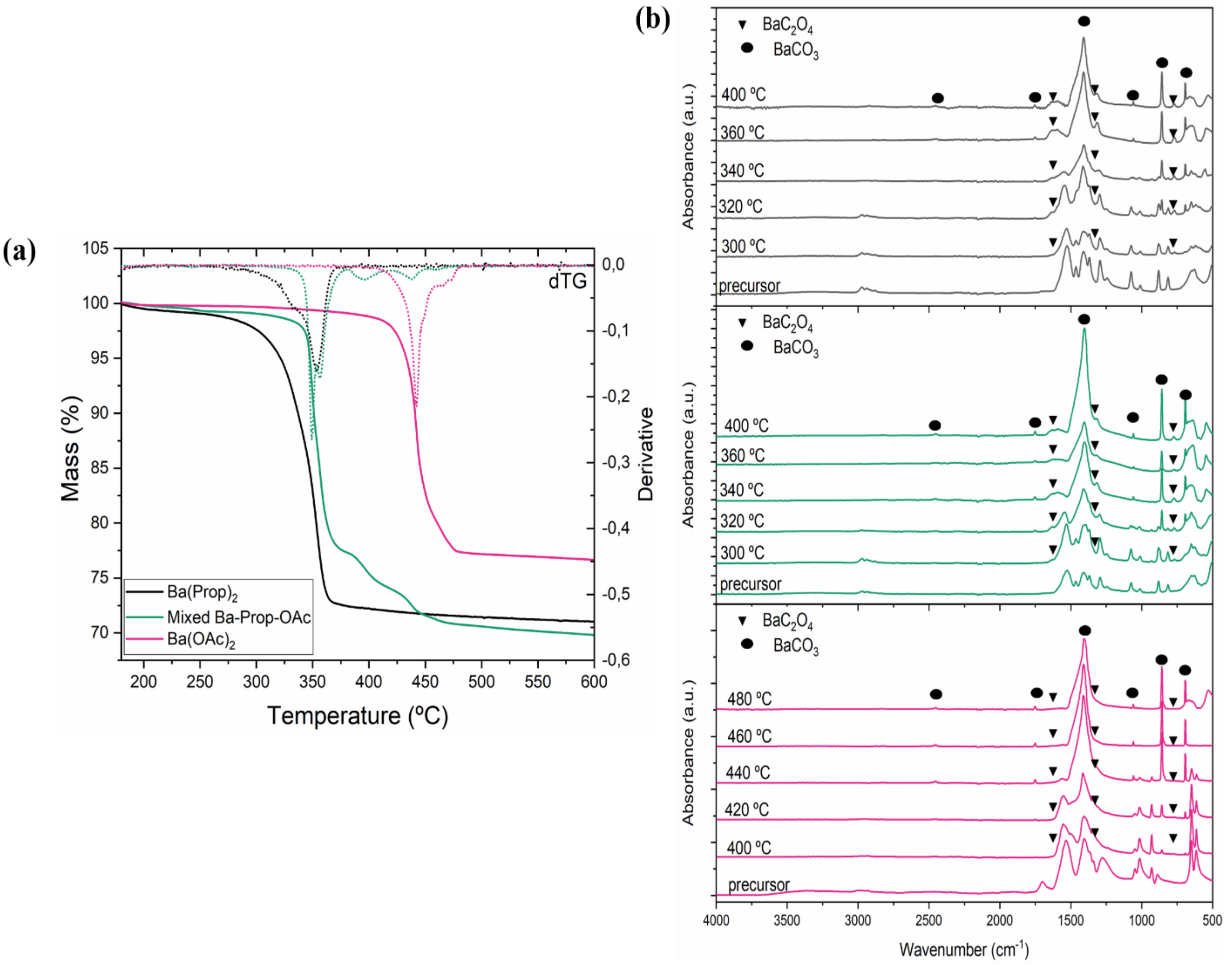

Figure S21. Initial formation of BaC_2O_4 can be observed already in the film quenched at 320 ºC, where the contribution of peaks deriving from the precursor is decreasing; at 340 ºC, only peaks relative to the oxalate and the final carbonate are identified, suggesting the partial formation of the final product coexisting with the oxalate species. Considering its formation temperature and the products present at the moment of the oxalate formation, we believe its origin may be found in the oxidation of the remaining propionate groups by means of the evolved acetaldehyde. (a)

Comparison of the thermal behavior of three solutions from different precursors of barium: a solution of $Ba(Prop)_2$ prepared using the precursor described in the manuscript (black), being thus the case of only propionate groups in solution; a solution of $Ba(OAc)_2$ dissolved in HProp and MeOH (1:1), thus yielding the mixed propionate-acetate complex of barium (dark green); a solution of $Ba(OAc)_2$ dissolved in HOAc and MeOH (1:1), consisting in a solution in which only acetate groups are present (dark pink). Important to notice the different decomposition temperatures for the three cases, being the acetate the most stable with the main mass loss at 450 ºC, 100 ºC higher than the other two cases. The case of the mixed propionate-acetate complex displays a main mass loss at similar temperature as the full-propionates case, presumably due to the decomposition of the propionate groups; at higher temperature, it presents two consecutive, small mass losses, which are given by the decomposition of the acetate groups (confirmed by evolution of formaldehyde, characteristic gas evolved from the decomposition of an acetate, shown in the following Figure S22). (b) Series of quenches for the same three samples in (a), which allowed us to determine the presence of the oxalate group in samples which contain propionate groups, confirming the formation of the oxalate to be characteristic to this functional group decomposition pathway. The absence of the oxalate peaks in the sample containing only acetates is a further confirmation of this hypothesis. Moreover, if comparing the two cases in which propionates are present in solution, it is clear that, in both, the oxalate formation occurs at a similar temperature, suggesting it is indeed deriving from the decomposition of the propionate groups as it is corresponding to the first mass loss in the sample of mixed propionate-acetate complex.

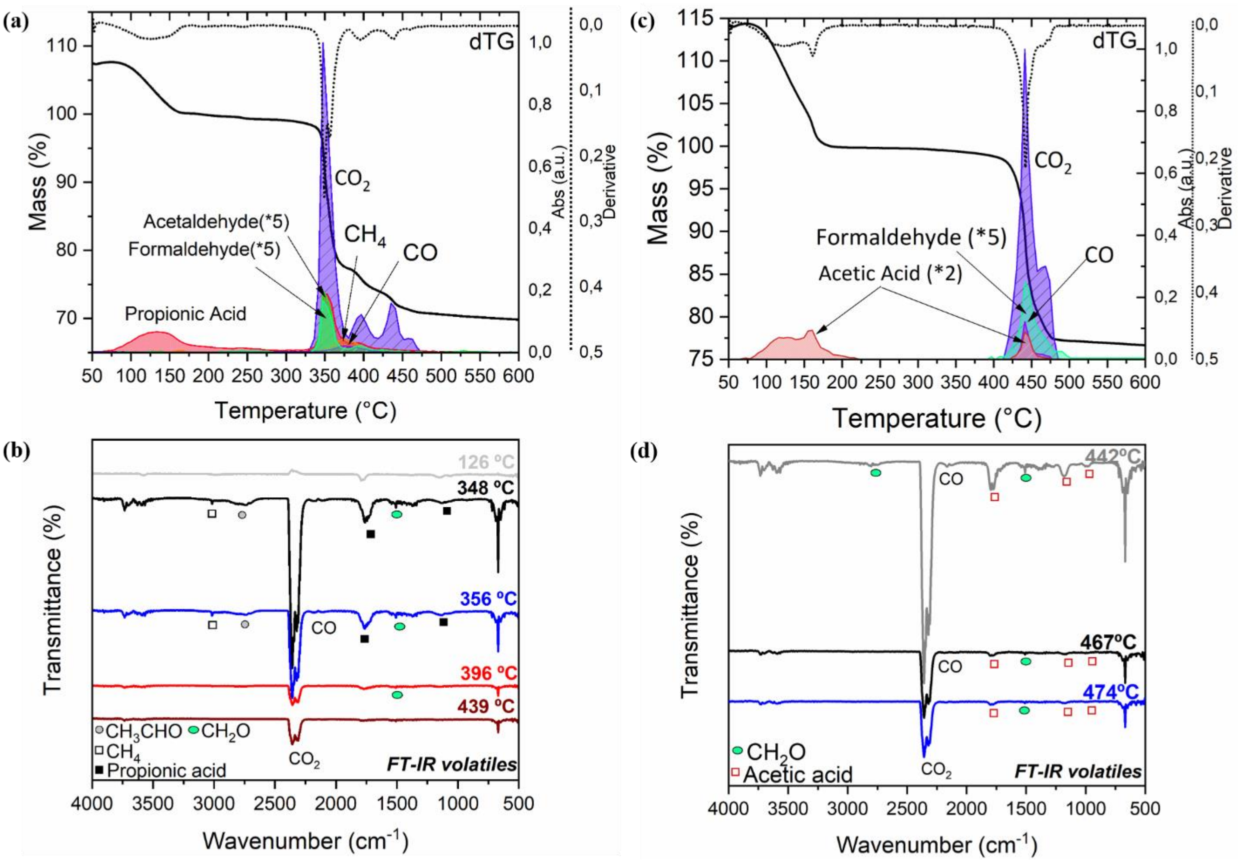

Figure S22. (a) and (b) TG profile (solid black line), time derivative dTG (dotted black line), and gas evolution of a solution of Ba(OAc)$_2$ in HProp:MeOH (1:1), yielding a solution in which both propionate and acetate groups are present. Important to notice the presence of formaldehyde, formed during the final mass losses (between 350 ºC and 450 ºC), due to the decomposition of the acetate groups, successive to the full decomposition of the propionate groups. (c) and (d), TG profile (solid black line), time derivative dTG (dotted black line) and gas evolution of a solution of Ba(OAc)$_2$ in HOAc:MeOH (1:1), thus consisting in a solution in which only acetate groups are present. In oxidative atmosphere, the proposed mechanism is the degradative oxidation of the acetate groups to form acetic acid, formaldehyde, and CO_2. The analysis of the evolved gases from this sample are fundamental to confirm the decomposition pathway of the case of the mixed propionate-acetate complex, and consequently the fact that the formation of barium oxalate is solely due to the decomposition of propionate groups, shedding light on Ba(Prop)$_2$ thermal behavior.

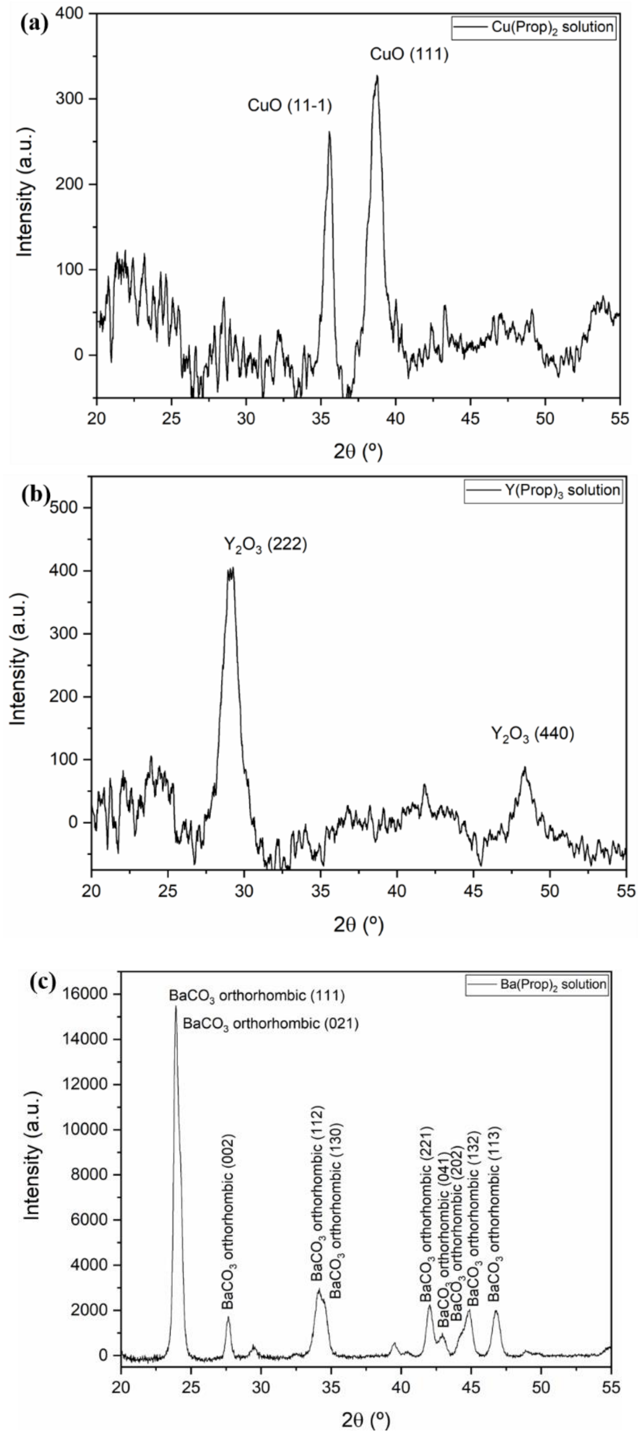

Figure S23. XRD data of the final residue from the decomposition of films of (a) a solution of $Cu(Prop)_2$, (b) a solution of $Y(Prop)_3$, (c) a solution of $Ba(Prop)_2$.

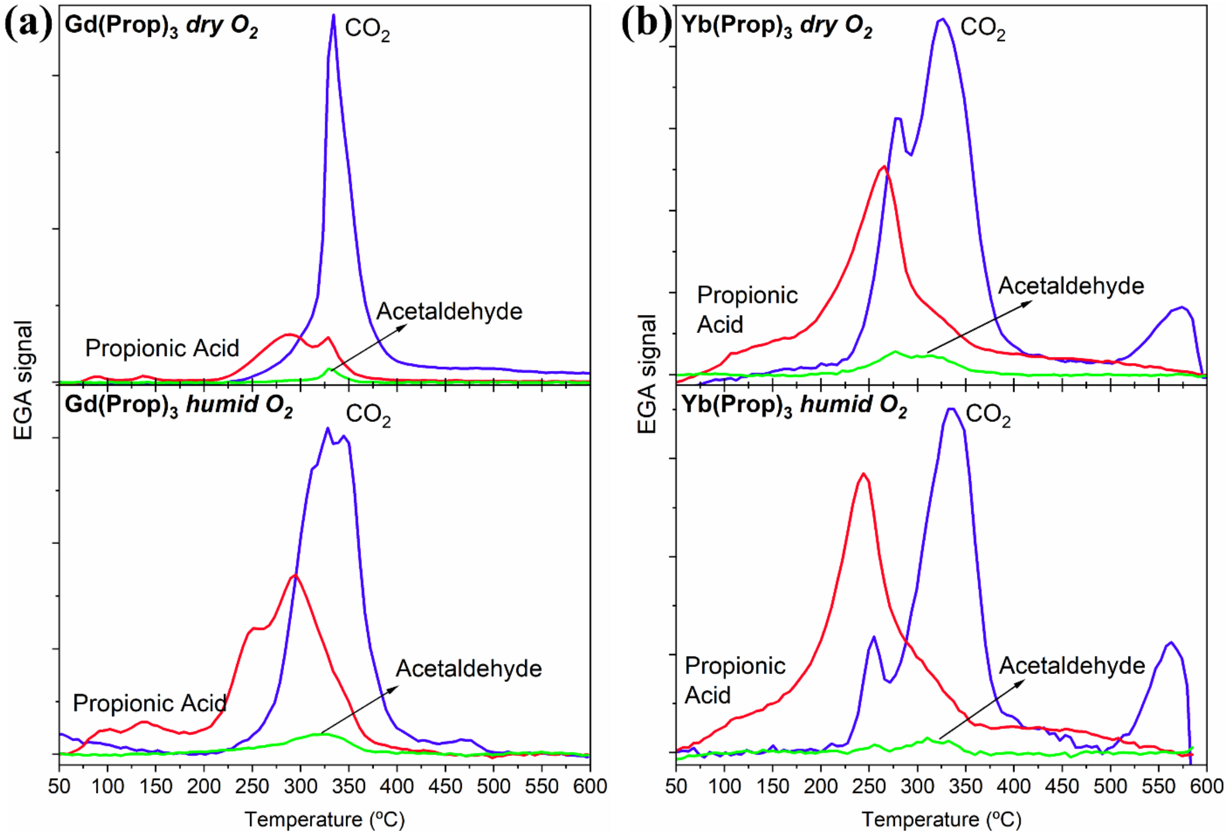

Figure S24. Analysis of the evolved gases comparing decomposition in dry (upper panels) and humid (lower panels) oxidative atmosphere during the thermal treatment for (a) Gd(Prop)$_3$ and (b) Yb(Prop)$_3$.

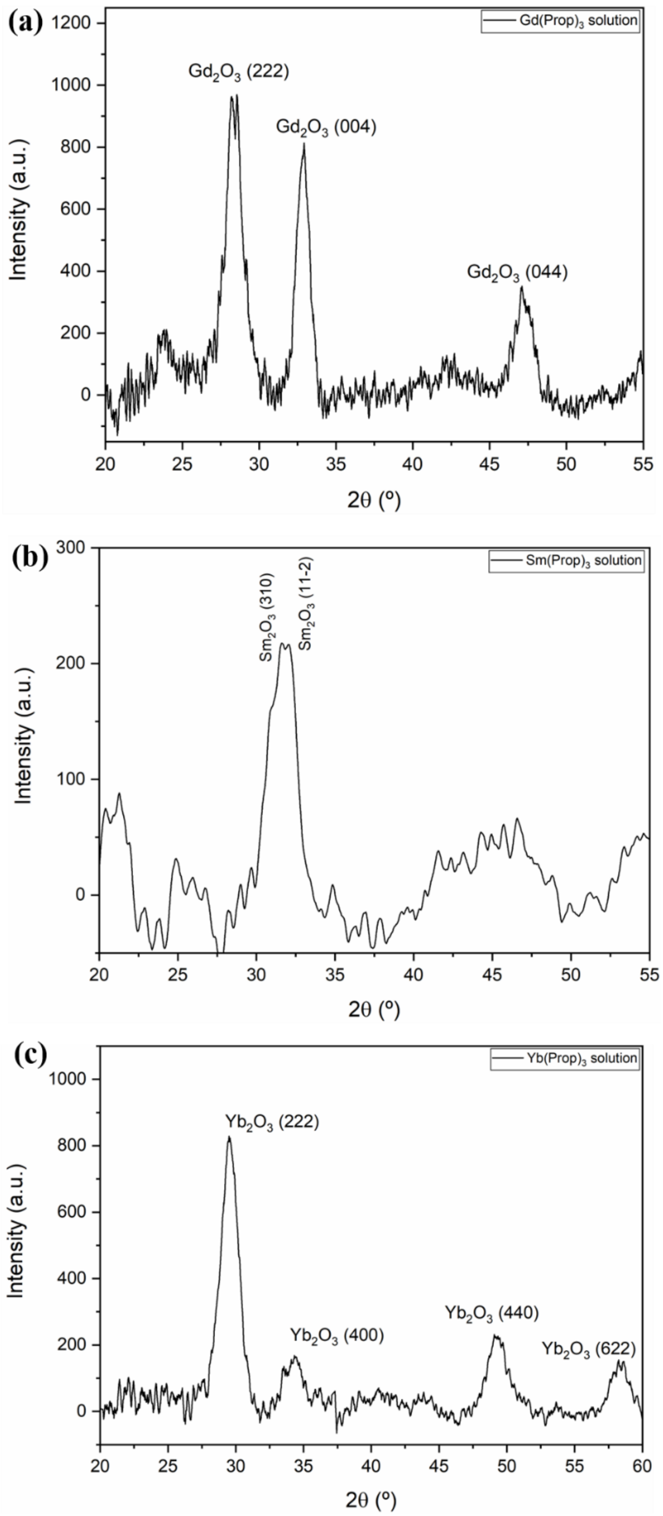

Figure S25. XRD data of the final residue from the decomposition of films of (a) a solution of Gd(Prop)$_3$, (b) a solution of Sm(Prop)$_3$, (c) a solution of Yb(Prop)$_3$.

Section IV. Rheological analysis of REBCO precursor solution

REBCO precursor solution	Concentration (M)	Water content on day of preparation (wt%)	Viscosity (mPa·s)	Contact Angle (º)
YBCO	1.5M + 3.4%$_{v/v}$ MEA	0.645 ± 0.06	8.680 ± 0.05	19.4 ± 0.1
SmBCO	1.5M + 3.4%$_{v/v}$ MEA	0.656 ± 0.06	8.965 ± 0.05	12.3 ± 0.1
GdBCO	1.5M + 3.4%$_{v/v}$ MEA	0.434 ± 0.06	8.943 ± 0.05	19.1 ± 0.1
YbBCO	1.5M + 3.4%$_{v/v}$ MEA	0.604 ± 0.06	8.664 ± 0.05	13.2 ± 0.1

Table S13. Rheological properties of REBCO precursor solutions compared to a YBCO precursor solution.

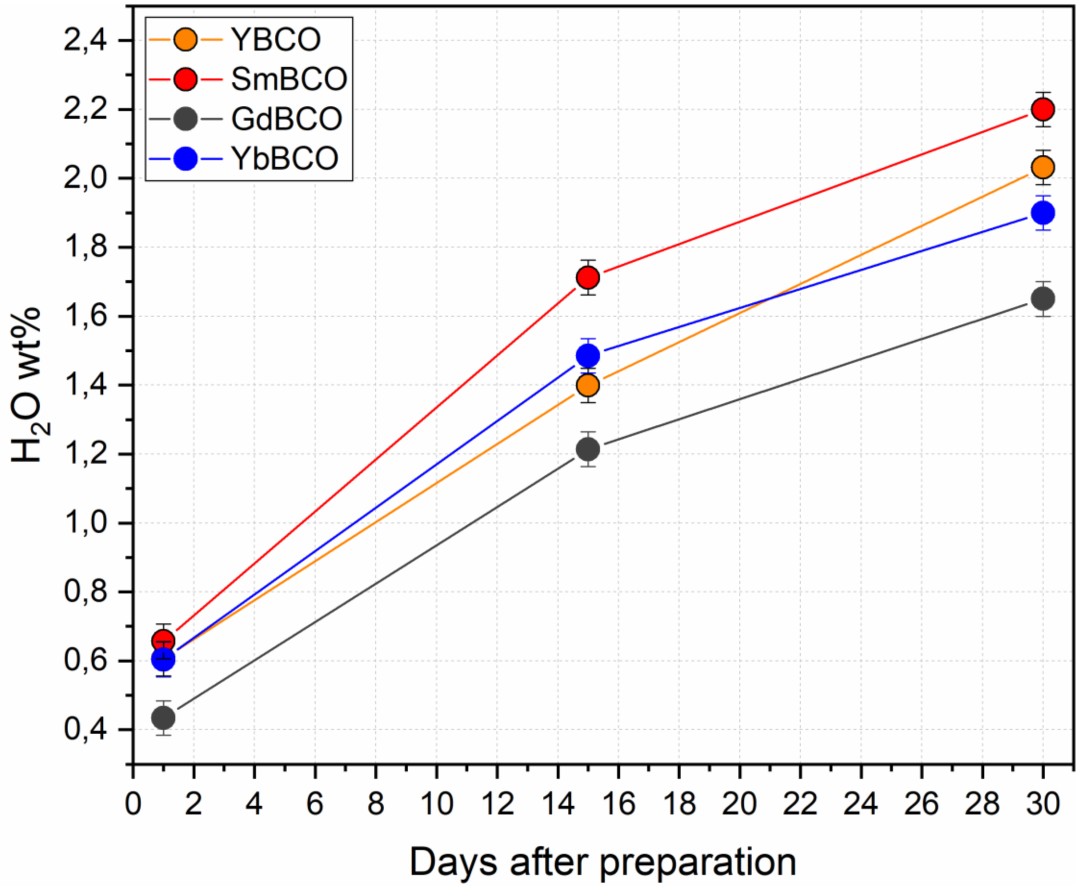

Figure S26. Water content (H_2O wt%) of REBCO precursor solutions compared to a YBCO precursor solution.

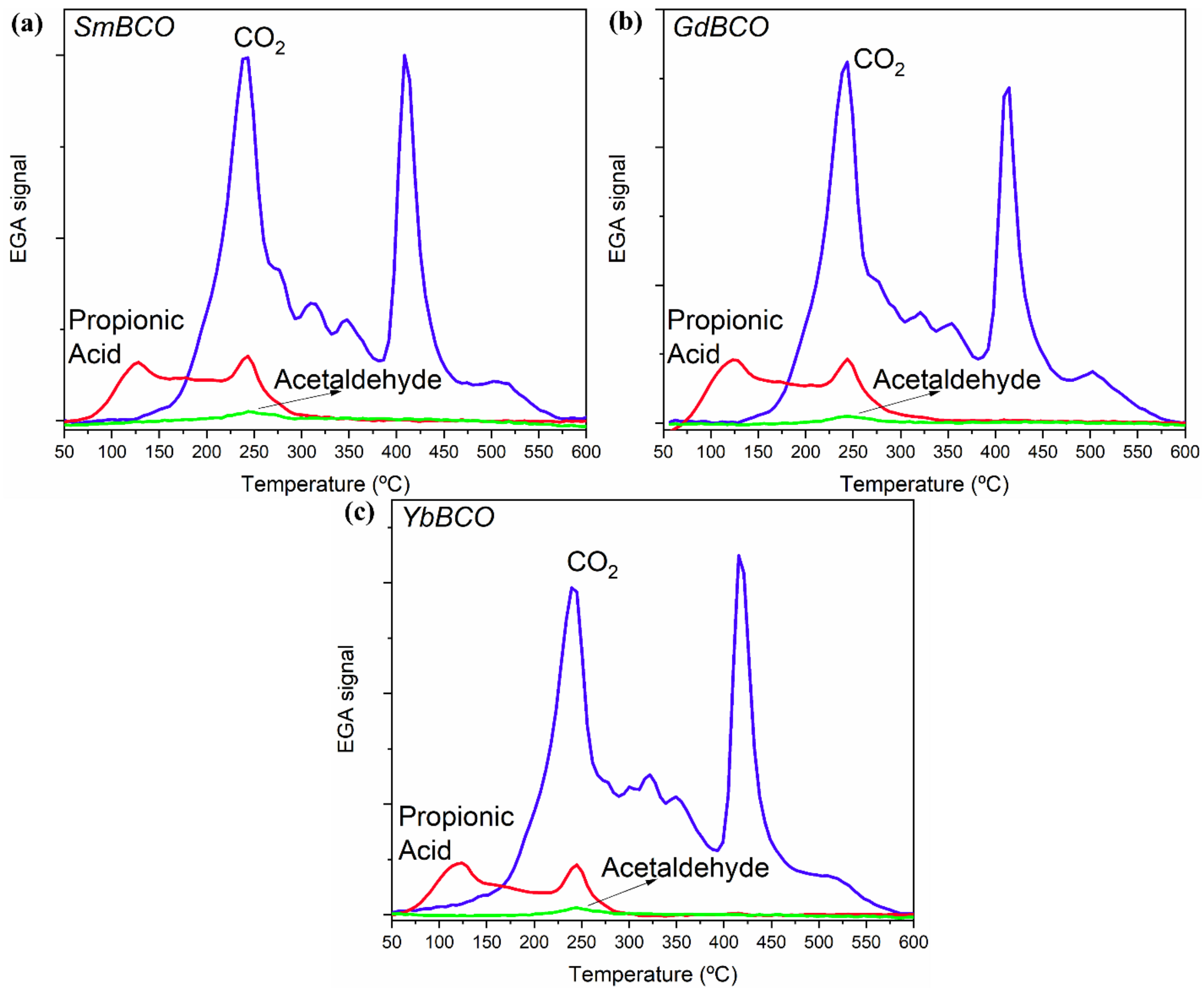

Figure S27. EGA-FTIR data of the gases released during decomposition for solutions of (a) SmBCO, (b) GdBCO, and (c) YbBCO.

Section V. REBCO nanocrystalline films

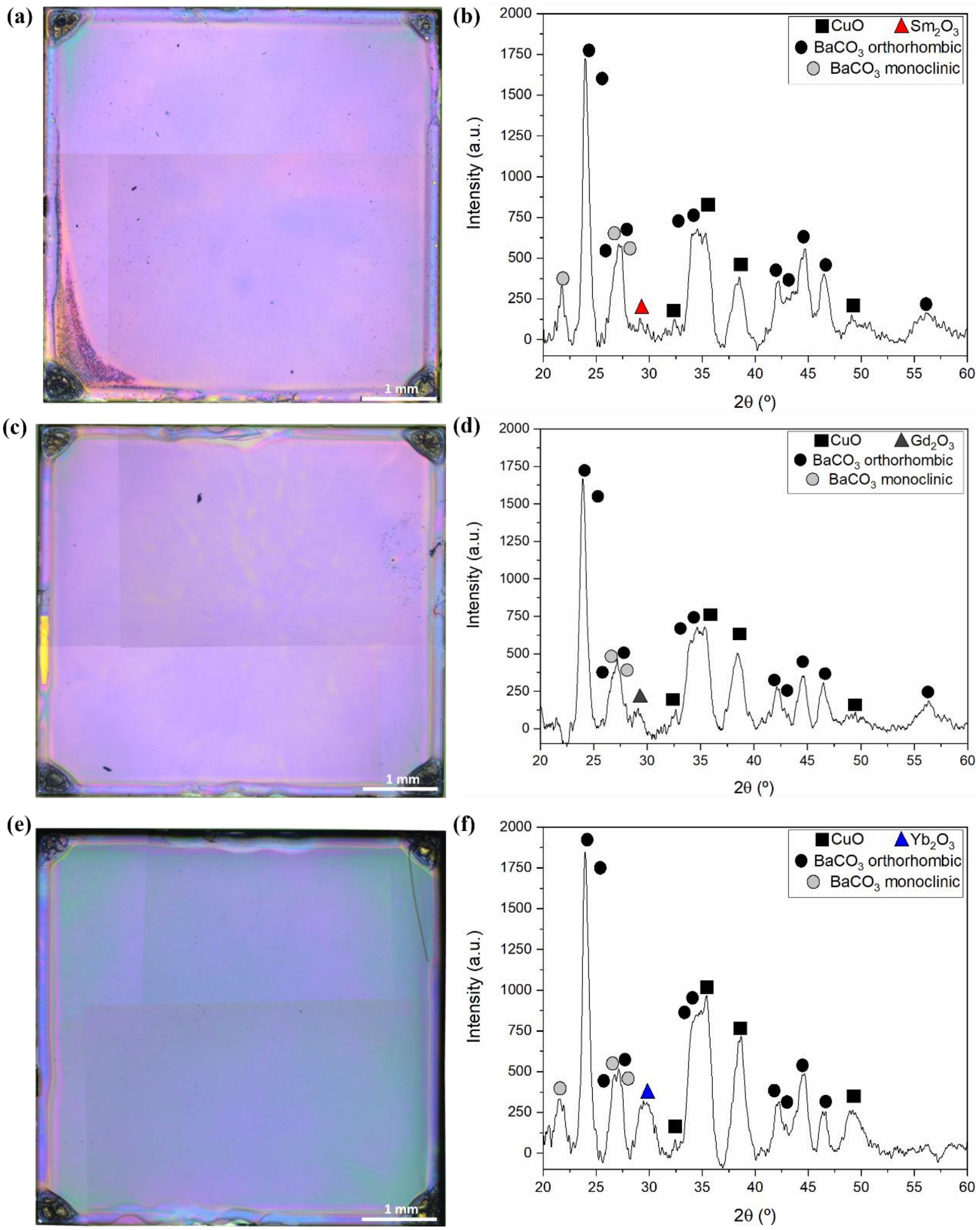

Figure S28. (a) OM image of SmBCO nanocrystalline precursor film with relative XRD data shown in (b); (c) OM image of GdBCO nanocrystalline precursor film with relative XRD data shown in (d); (e) OM image of YbBCO nanocrystalline precursor film with relative XRD data shown in (f).

Section VI. Superconducting properties of epitaxial REBCO films.

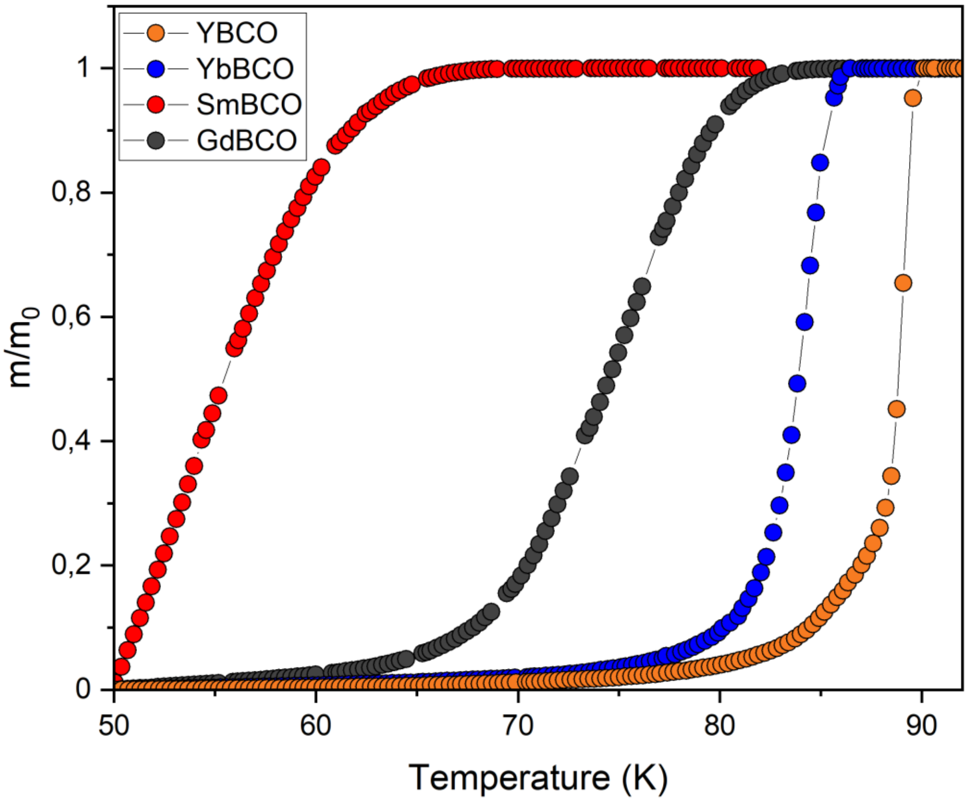

Figure S29. T_c curves measured through SQUID magnetometry of the four REBCO systems described in this manuscript. The wide transitions of the SmBCO, GdBCO, and YbBCO films compared to the case of YBCO confirm the need for a further optimization of the processing parameters.

References

(1) Rasi, S.; Silveri, F.; Ricart, S.; Obradors, X.; Puig, T.; Roura-Grabulosa, P.; Farjas, J. Thermal Decomposition of CuProp 2 : In-Situ Analysis of Film and Powder Pyrolysis. *J Anal Appl Pyrolysis* **2019**, *140*, 312–320. https://doi.org/10.1016/j.jaap.2019.04.008.

(2) Chung, Y. H.; Wei, H. H.; Liu, Y. H.; Lee, G. H.; Wang, Y. *Reinvestigation of the Crystal Structure and Cryomagnetic Behaviour of Copper(Ll) Propionates*; 1998; Vol. 17.

(3) Bierke, T. CCDC 832100: Experimental Crystal Structure Determination, 2013. https://doi.org/10.5517/ccwxvyk.

(4) Llunell, M. ; Casanova, D. ; Cirera, J. ; Bofill, J. M. ; Alemany, P. ; Alvarez, S. ; Pinsky, M. ;; Avnir, D. SHAPE. Universitat de Barcelona and Hebrew University of Jerusalem, 2013.

(5) Pinsky, M.; Avnir, D. Continuous Symmetry Measures. 5. The Classical Polyhedra. *Inorg Chem* **1998**, *37* (21), 5575–5582. https://doi.org/10.1021/ic9804925.

(6) Barnes, P. A.; Stephenson, G.; Warrington, S. B. *THE USE OF TA-GLC-MS AS A QUANTITATIVE SPECIFIC EGA TECHNIQUE FOR THE INVESTIGATION OF COMPLEX THERMAL DECOMPOSITION REACTIONS: THE THERMAL DECOMPOSITION OF CALCIUM PROPANOATE*; 1982; Vol. 25.

(7) Grivel, J. C. Thermal Decomposition of Ln(C 2H 5CO 2) 3•H 2O (Ln = Ho, Er, Tm and Yb). *J Therm Anal Calorim* **2012**, *109* (1), 81–88. https://doi.org/10.1007/s10973-011-1745-9.

(8) Sola Akanni, M.; Burrows, H. D.; Bay0 Begun, P. *PRODUCT ANALYSIS, REACTION MECHANISM AND KINETICS OF THE THERMAL DECOMPOSITION OF SOME EVEN CHAIN-LENGTH MERCURY(B) CARBOXYLATES*; Elsevier Science Publishers B.V, 1984; Vol. 81.

(9) Sola Akanni, M.; Okoh, E. K.; Burrows, H. D.; Ellis', H. A. *The Thermal Behaviour of Divalent and Higher Valent Metal Soaps: A Review*; 1992; Vol. 208.
